# Comparative Efficacy and Safety of Resmetirom and Efruxifermin for Metabolic Dysfunction‐Associated Steatohepatitis: A Network Meta‐Analysis of Randomized Controlled Trials

**DOI:** 10.1002/edm2.70218

**Published:** 2026-04-07

**Authors:** Doha Jaber, Inas Jaber, Ayah Abu Lehia, Thekrayat Asad, Hazem Ayesh

**Affiliations:** ^1^ Faculty of Medicine Al‐Quds University Jerusalem Palestine; ^2^ Department of Internal Medicine Unity Hospital, Rochester Regional Health Rochester New York USA; ^3^ Deaconess Health System Evansville Indiana USA

**Keywords:** Efruxifermin, Hepatology, Metabolic dysfunction–associated steatohepatitis, network meta‐analysis, Non‐alcoholic steatohepatitis, Resmetirom

## Abstract

**Background and Aim:**

Metabolic dysfunction‐associated steatohepatitis (MASH) is a progressive liver condition and a major cause of cirrhosis, hepatocellular carcinoma, and liver transplantation. Resmetirom, a thyroid hormone receptor β agonist, and Efruxifermin, a fibroblast growth factor 21 analogue, have shown promise in improving hepatic fat fraction (HFF) and liver enzyme levels. This study systematically compares the efficacy and safety of Resmetirom and Efruxifermin in treating MASH.

**Methods:**

A systematic search of PubMed, Cochrane, and Scopus identified 211 studies, of which eight randomized controlled trials (RCTs) were included. Primary outcomes included reductions in liver enzyme levels. Secondary outcomes assessed HFF measured by magnetic resonance imaging‐derived proton density fat fraction (MRI‐PDFF), as well as lipid profiles. Safety outcomes consist of serious adverse events and events leading to treatment discontinuation.

**Results:**

Efruxifermin was associated with significant improvement in MRI‐PDFF with an mean difference (MD) of −62.83% (95% CI: −72.30 to −53.36, *p* = 0.00), followed by Resmetirom with an MD of −37.15% (95% CI: −44.43 to −29.88, *p* = 0.00), additionally Efruxifermin was associated with significant reduction in aspartate transferase (AST) level, with an MD of −14.32 (95% CI: −23.92 to −4.72, *p* = 0.003), compared to Resmetirom (MD: −2.81; 95% CI: −12.40 to 6.79, *p* = 0.56). For lipid profiles, Efruxifermin showed a significant reduction in triglyceride levels with an MD of −36.95 (95% CI: −52.67 to −21.24, *p* = 0), while Resmetirom had an MD of −24.72 (95% CI: −33.31 to −16.14, *p* = 0.00).

**Conclusion:**

Efruxifermin demonstrated a slightly greater effect on MRI‐PDFF and AST, along with more favourable safety outcomes.

## Graphical abstract

This meta‐analysis compared Resmetirom and Efruxifermin in patients with MASH, focusing on improvements in key biochemical and non‐invasive markers. Efruxifermin is associated with favourable outcomes with a slightly greater effect on MRI‐PDFF and AST, along with more comparable safety outcomes.

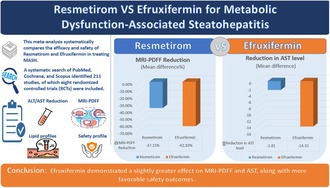

**Abbreviations**
ALT: Alanine aminotransferase
AST: Aspartate aminotransferase
BMI: Body mass index
CINeMA: Confidence in Network Meta‐Analysis
DM: Diabetes mellitus
FDA: the Food and Drug Administration
FGF21: Fibroblast growth factor 21
GLP‐1: Glucagon‐like peptide‐1
HCC: Hepatocellular carcinoma
HDL: High‐density lipoproteins
HFF: Hepatic fat fraction
HTN: Hypertension
LDL: Low‐density lipoproteins
MASH: Metabolic dysfunction‐associated steatohepatitis
MASLD: Metabolic dysfunction‐associated steatotic liver disease
MD: Mean difference
MRI‐PDFF: Magnetic resonance imaging‐derived proton density fat fraction
NASH: Non‐alcoholic steatohepatitis
OSF: Open Science Framework
PRISMA: Preferred Reporting Items for Systematic Reviews and Meta‐Analyses
RCT: Randomized controlled trial
RCTs: Randomized controlled trials
RR: Relative risks
SAEs: Serious adverse events
SE: Standard error
TEAEs: Treatment‐emergent adverse events
THR: Thyroid hormone receptor

## 1 Introduction

Metabolic dysfunction‐associated steatohepatitis (MASH) is a condition characterized by chronic liver injury and inflammation, representing the more severe form of metabolic dysfunction‐associated steatotic liver disease (MASLD) [1]. It features steatosis, hepatocellular ballooning, and lobular inflammation, and may include varying degrees of fibrosis, which can progress to cirrhosis and hepatocellular carcinoma (HCC) [1, 2]. The prevalence of MASH has risen alongside the increasing prevalence of obesity and metabolic syndrome worldwide; also, MASH‐associated HCC has increased 10‐fold in the past decades [3]. Currently, MASH HCC is considered a major indication for liver transplantation worldwide [3, 4, 5]. Therefore, understanding the global burden and progression of MASH and MASLD, along with their hepatic and extrahepatic complications, is essential for effective patient management [6, 7].

The mainstay of management of MASH is lifestyle intervention and physical activity [8]. Although various pharmacological agents have been studied, with variable efficacy, Resmetirom (MGL‐3196) is a liver‐targeted, thyroid hormone receptor (THR)‐β‐selective drug that has been approved as the first drug for treating MASH and associated fibrosis, offering a new pharmacological option for patients with moderate to advanced fibrosis without cirrhosis [9, 10, 11].

Efruxifermin, a bivalent Fc‐fibroblast growth factor 21 (FGF21) analogue, has diverse metabolic benefits in the treatment of MASH and dyslipidemia [12]. A recent systematic review and network meta‐analysis indicate that Efruxifermin has significant efficacy in treating MASH without worsening fibrosis, alongside its favourable tolerability profile and its role in reducing body weight and liver fat content [10]. These results provide evidence for Efruxifermin as a promising therapeutic agent for MASH‐related fibrosis. However, a major gap in the literature is the absence of a direct head‐to‐head comparison between therapeutic agents, which is considered essential to validate treatment results [10, 13, 14].

The recent approval of specific therapies for MASH underscores the progress in the field; however, the continued concentration in histologic and clinical endpoints across trials highlights our goal to present non‐invasive measurements of Hepatic fat fraction (HFF) measured by magnetic resonance imaging‐derived proton density fat fraction (MRI‐PDFF) and biochemical outcomes as key markers of metabolic activity and treatment response to refined strategies to optimize patient selection, safety monitoring, and efficacy assessment [15]. Given the complex pathophysiology of MASH and the diverse mechanisms of emerging agents, direct head‐to‐head RCTs between all available therapies are impractical. Therefore, a network meta‐analysis is essential to integrate evidence across trials and enable indirect comparisons to clarify the relative efficacy and safety profiles and guide therapeutic decision‐making [15, 16, 17].

In our study, we systematically compare the efficacy and safety of Resmetirom and Efruxifermin in treating MASH using the Network Meta‐analysis approach to synthesize clinical evidence. We focus on non‐invasive approaches—specifically HFF (MRI‐PDFF) and biochemical markers—to address a significant gap in the existing evidence for personalized management of MASH.

## 2 Materials and Methods

The study protocol is registered on the Open Science Framework (OSF) at (https://osf.io/98p6u, accessed on May 3, 2025). The Preferred Reporting Items for Systematic reviews and Meta‐Analyses (PRISMA) checklist was followed throughout the study in Supporting Information S11.

### 2.1 Search Strategy

A comprehensive literature search for randomized controlled trials was performed across PubMed, Cochrane, and Scopus to identify relevant studies from the inception of the databases to January 25, 2025. The search terms included: (((MASH OR (Metabolic dysfunction‐associated steatohepatitis) OR (NASH OR Nonalcoholic Steatohepatitis OR nonalcoholic fatty liver disease OR NAFLD OR Metabolic Dysfunction‐Associated Steatotic Liver Disease OR MASLD)) AND ((Resmetirom OR MGL‐3196) OR (thyroid hormone receptor‐β agonist)) OR (Efruxifermin OR AKR‐001)) AND (RCT OR trial OR (Randomized Controlled Trial))). The detailed search strategy is reported in Supporting Information S1.

### 2.2 Screening Process

Two authors (IJ and A‐AL) independently screened the titles and abstracts of retrieved studies. Full‐text articles were reviewed for eligibility. Discrepancies were resolved through discussion with a third author (DJ) if necessary.

### 2.3 Eligibility Criteria and Study Selection

We included RCTs evaluating **Resmetirom** or **Efruxifermin** in adults with **MASH**, with or without fibrosis. Eligible studies were required to compare these agents with placebo in adult patients with biopsy‐proven MASH, with a minimum follow‐up duration of 12 weeks. In line with the American Association for the Study of Liver Diseases (AASLD), our study emphasizes the efficacy endpoints, including changes in liver enzymes (ALT, AST), HFF assessed by MRI‐PDFF, and changes in metabolic or lipid profiles [18]. Safety outcomes included serious adverse events, treatment discontinuations, and common adverse effects such as diarrhoea and nausea. Only studies published in English were considered for inclusion.

Studies were excluded if they involved paediatric populations, patients with chronic liver diseases other than MASH, or populations that did not meet MASH diagnostic criteria. Trials were also excluded if they did not evaluate Resmetirom or Efruxifermin, if they were published in languages other than English, if only an abstract was available without full‐text access, or if they were non‐comparative designs such as case reports, case series, or review articles.

### 2.4 Data Extraction

Data extracted from each eligible study included the following information: baseline characteristics, intervention details, and outcome measures (Table 1, Supporting Information S2). Specifically, age, gender, body mass index (BMI), diabetes (DM), hypertension (HTN), liver enzyme levels, MRI‐PDFF, and serum lipid parameters, e.g., high‐density lipoprotein (HDL), low‐density lipoprotein (LDL), total cholesterol, and triglyceride levels were extracted (Table S3) [19, 20, 21, 22, 23, 24, 25, 26]. Safety outcomes, including treatment discontinuation due to adverse events and serious events, were also obtained. Data on study design, randomization, study duration, and phase were also extracted.

When the data were presented graphically, we used WebPlot Digitizer version 4.3 to estimate the values [27].

### 2.5 Statistical Analysis

To determine the relative efficacy of each treatment, we conducted a network meta‐analysis to compare one‐to‐one comparisons through the analysis of the included studies. Statistical analyses were conducted using a random‐effects model to account for heterogeneity among studies. MD for continuous outcomes and relative risks (RR) for dichotomous outcomes were calculated. Heterogeneity was evaluated using tau‐squared (*τ* ^2^), *I* ^2^ statistics, and *Q* statistics to assess consistency under the assumption of a full design‐by‐treatment interaction random effects model [28]. P‐scores were used to rank treatments based on their estimated effect sizes. Egger's test was used to assess funnel plot asymmetry by testing for a linear relationship between effect size and study precision (See Figures S5.1–S5.11) [29]. Significant results (*p* < 0.05) may indicate publication bias, though other factors can also cause asymmetry.

The outcomes evaluated include the following: Changes in liver enzyme levels include ALT and AST, which are indicators of liver injury; MRI‐PDFF measures the alteration in liver fat content over the study period; and lipid profiles. Safety outcomes include serious adverse events, treatment discontinuation due to treatment‐emergent adverse events (TEAEs), and the incidence of nausea and diarrhoea among participants. Leave‐out approach evaluated sensitivity, and meta‐regression analysis was performed using different statistical techniques to investigate the influence of study characteristics on the outcomes.

Statistical analysis for this network meta‐analysis was conducted using R (version 4.5.0), alongside using netmeta (version 3.2.0), which enables indirect comparisons among treatment arms [30, 31]. Confidence in Network Meta‐Analysis (CINeMA) framework, endorsed by the Cochrane Collaboration was used to determine the certainty of evidence of all estimates [32].

### 2.6 Risk of Bias of Individual Studies

We used the Cochrane randomized trial Risk of Bias tool (version 2.0) to assess the risk of bias in the included trials, including these domains: randomization, allocation concealment, blinding of participants and personnel, measurement of the outcome, missing outcome data, and selective reporting (Supporting Information S12) [33]. Two authors (DJ and A‐AL) independently assessed the risk of bias. Disagreements were resolved through discussion or by consulting a third author (IJ).

## 3 Results

### 3.1 Study Selection and Characteristics

**FIGURE 1:**
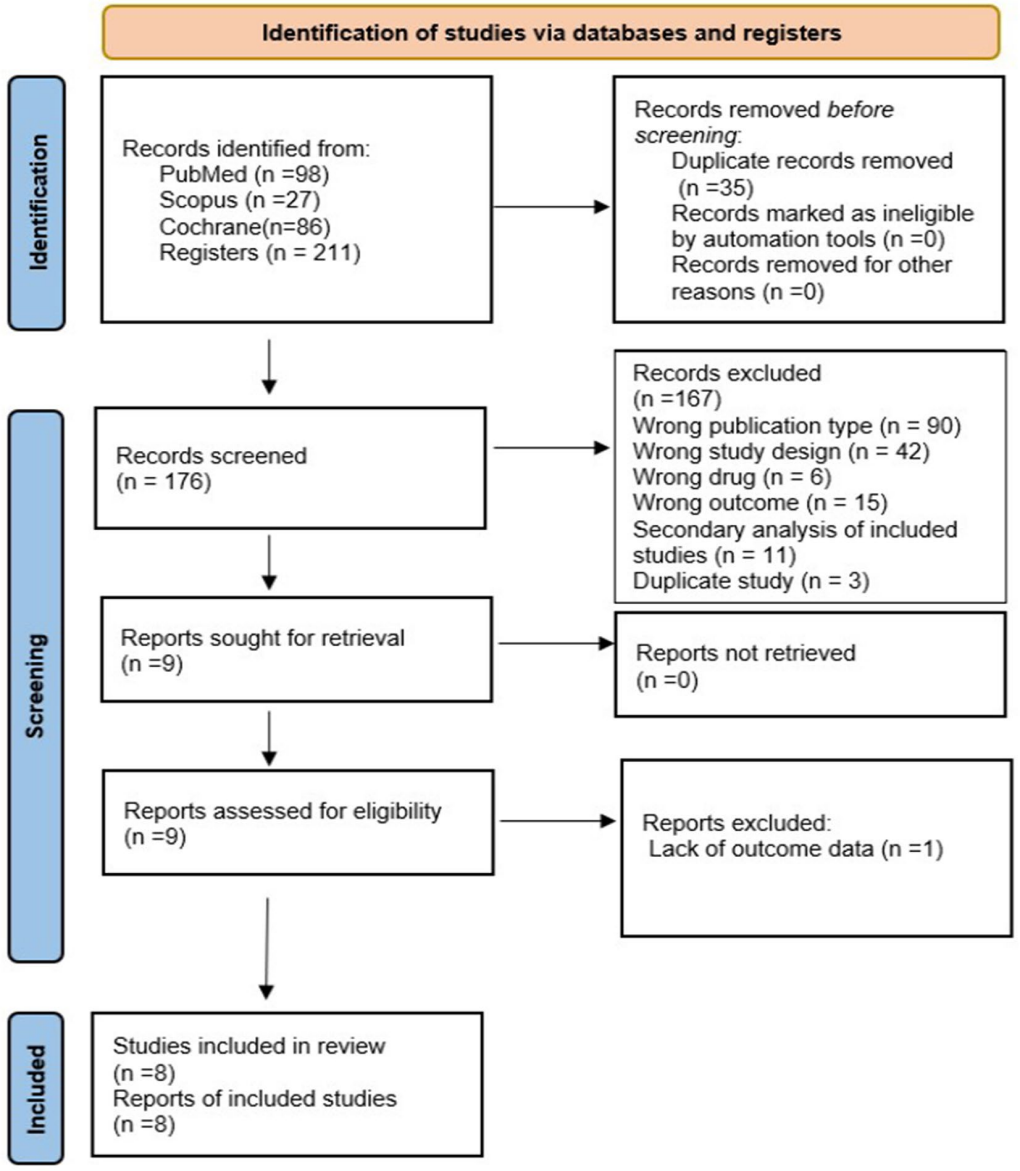
PRISMA flowchart of study screening and selection. PRISMA, Preferred Reporting Items for Systematic Reviews and Meta‐Analyses.

We identified 211 trials using the search strategy, and 35 duplicates were eliminated. After 176 trials were screened for titles and abstracts, nine trials were received for full‐text screening. Finally, a total of eight RCTs with 2534 participants were included in the systematic review and network meta‐analysis (Table 1, Supporting Information S2) [19, 20, 21, 22, 23, 24, 25, 26]. Four studies evaluated the efficacy and safety of daily Resmetirom; four studies evaluated the efficacy and safety of once‐weekly subcutaneous Efruxifermin; and one study was excluded due to a lack of outcome data (see Supporting Information S9) [34]. The details of study screening and trial selection are illustrated in the PRISMA flowchart (Figure 1). The mean age was 55.67 years (SD 11.41), with 43.67% of participants being male and a mean BMI of 35.72 kg/m2 (SD 6.43). Liver function tests showed a mean ALT level of 46.43 IU/L (SD 30.58) and a mean AST level of 33.79 IU/L (SD 21.55). Glycaemic control, measured by HbA1c, had a mean of 6.49% (SD 1.08). Lipid profiles showed a mean triglyceride level of 109.47 mg/dL (SD 114.04), a mean LDL level of 107.54 mg/dL (SD 37.47), and a mean HDL level of 43.67 mg/dL (SD 13.14).

### 3.2 Sensitivity Analysis and Meta‐Regression

We conducted a meta‐regression analysis to ensure validity of our findings. In our meta‐regression, we included key variables such as study duration, age, male gender, and BMI (See Figures S10.1–S10.11). Sensitivity analysis was conducted using a leave‐one‐out approach for each outcome, in order to ensure robustness of the results and to evaluate the influence of individual studies on the overall pooled estimates.

### 3.3 Imaging Outcomes

#### 3.3.1 Percent Change in MRI‐PDFF

**FIGURE 2:**
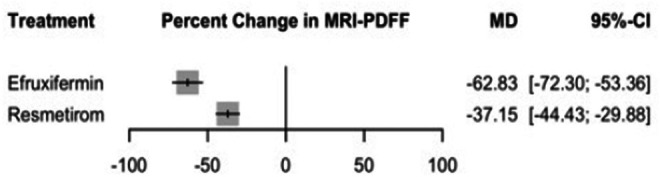
Network meta‐analysis results for percent change in MRI‐PDFF.

Efruxifermin was associated with significant improvement in MRI‐PDFF with an MD of −62.83% (95% CI: −72.30 to −53.36, *p* = 0.00), followed by Resmetirom with an MD of −37.15% (95% CI: −44.43 to −29.88, *p* = 0.00) (see Figure 2 and Supporting Information S7, Figure S7.1), in the indirect comparison between treatments, Efruxifermin was associated with a significant reduction in the MRI‐PDFF than Resmetirom with an MD of −25.68% (95% CI: −37.62 to −13.74) (Supporting Information S6, Table S6.1), and the corresponding network plot is shown in Figure S8.1. The heterogeneity analysis revealed moderate heterogeneity, with *I* ^2^ = 40.6%, *τ* ^2^ = 20.37. After conducting a leave‐one‐out analysis and excluding Harrison 2021_b, heterogeneity decreased significantly to 0%. However, the treatment ranking remains unchanged. BMI, male, and duration differences among studies contributed significantly to the high heterogeneity and the meta‐regression analysis (Coef = −9.62, *p* = 0.01), (Coef = 287.47, *p* = 0.05), and (Coef = 0.68, *p* = 0.05), respectively. Among treatment arms, Efruxifermin ranked highest based on network analysis with a *p*‐score of 1.00. According to the CINeMA framework, the certainty of evidence for the comparison between Resmetirom and Efruxifermin showed low certainty, due to major concerns with heterogeneity and some concerns regarding within‐study bias, reporting bias, and imprecision (Supporting Information S4, Table S4.1).

### 3.4 Biochemical Markers

#### 3.4.1 Change in ALT

**FIGURE 3:**
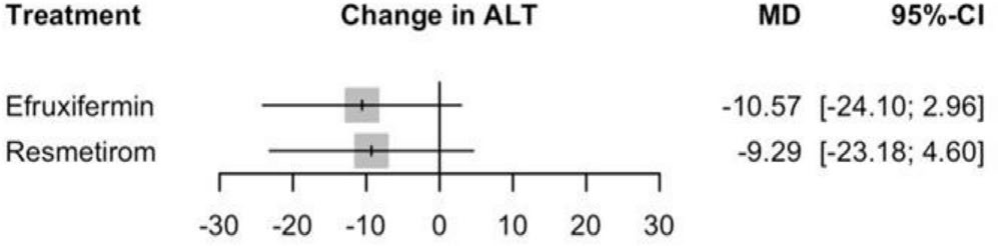
Network meta‐analysis results for change in ALT.

In the random effects model assessing the change in ALT, Efruxifermin demonstrated a non‐significant effect with an MD of −10.57 (95% CI: −24.10 to 2.96, *p* = 0.12). Resmetirom with an MD of −9.29 (95% CI: −23.18 to 4.60, *p* = 0.19), also not reaching significance (see Figure 3 and Supporting Information S7, Figure S7.2), no significant difference was observed between Efruxifermin and Resmetirom, MD of −1.28% (95% CI: −20.68 to 18.11) (Supporting Information S6, Table S6.2), and the corresponding network plot is shown in Figure S8.2. The heterogeneity analysis revealed significant heterogeneity with an *I* ^2^ of 90.1%. Tests of heterogeneity within designs were significant (Q = 60.46, df = 6, *p* < 0.0001). Despite conducting the leave‐one‐out analysis, the heterogeneity remains significant, indicating that no specific study was considered responsible for the result variability. The P‐scores, which rank treatments based on their effectiveness, were highest for Efruxifermin (p‐score = 0.74).

The certainty of evidence for the comparison between Resmetirom and Efruxifermin exhibited some concerns regarding within‐study bias and reporting bias. Major concerns were noted for imprecision and heterogeneity (Supporting Information S4, Table S4.2).

#### 3.4.2 Change in AST

**FIGURE 4:**
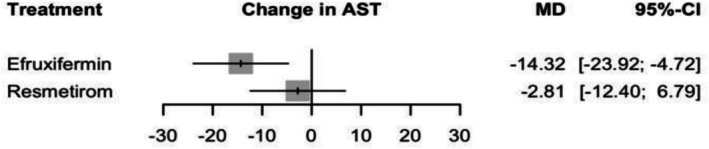
Network meta‐analysis results for change in AST.

Using a random effects model to assess the change in AST, Efruxifermin was associated with highest effect with an MD of −14.32 (95% CI: −23.92 to −4.72, *p* = 0.003). Resmetirom did not show a significant effect with an MD of −2.81 (95% CI: −12.40 to 6.79, *p* = 0.56) (see Figure 4 and Supporting Information S7, Figure S7.3), and no significant difference between the two treatments with an MD of −11.52 (95% CI: −25.09 to 2.06) (Supporting Information S6, Table S6.3), and the corresponding network plot is shown in Figure S8.3. Across all therapeutic options, Efruxifermin ranked highest regarding effectiveness with a p‐score of 0.97.

High heterogeneity was observed (*I* ^2^ = 90%, *τ* ^2^ = 85.49). Tests of heterogeneity within designs were significant (*Q* = 59.96, df = 6, *p* = 0.00). BMI difference among studies contributed significantly to the high heterogeneity and the meta regression analysis (Coef = −6.80, *p* = 0.02).

According to the CINeMA framework, the certainty of evidence for Resmetirom vs. Efruxifermin showed major concerns related to imprecision and heterogeneity. Also, there were some concerns regarding within‐study bias and reporting bias (Supporting Information S4, Table S4.3). The risk of publication bias was low.

### 3.5 Lipid Profiles

#### 3.5.1 Percentage Change in Triglyceride

**FIGURE 5:**
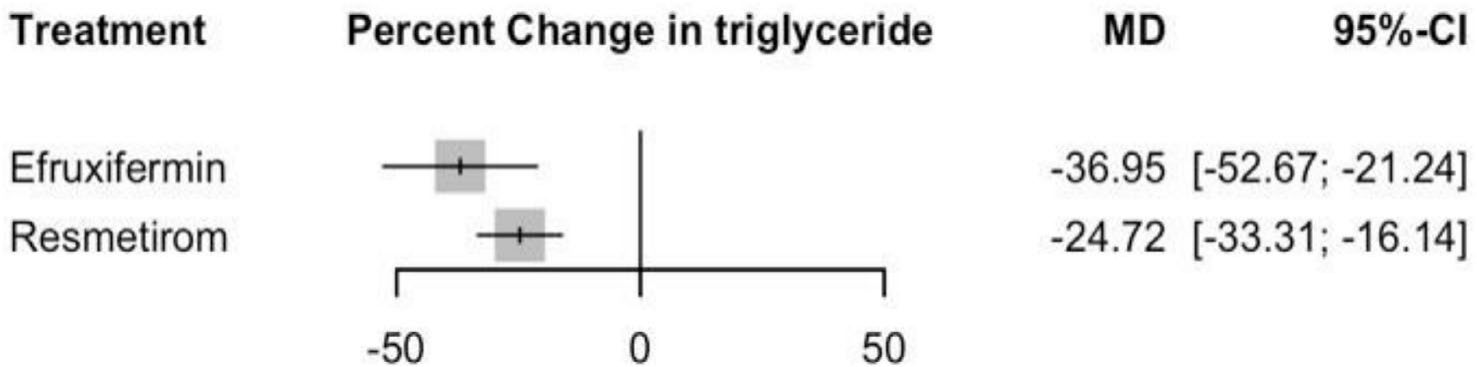
Network meta‐analysis results for percentage change in triglyceride.

Efruxifermin showed a significant change in triglyceride levels with an MD of −36.95% (95% CI: −52.67 to −21.24, *p* = 0), compared to Resmetirom with an MD of −24.72% (95% CI: −33.31 to −16.14, *p* = 0.00) (see Figure 5 and Supporting Information S7, Figure S7.4). No significant difference was observed in the indirect comparison between two treatments with an MD of −12.23% (95% CI: −30.14 to 5.68) (Supporting Information S6, Table S6.4), and the corresponding network plot is shown in Figure S8.4. The heterogeneity analysis revealed moderate heterogeneity (*I* ^2^ of 33.8%, *τ* ^2^ of 24.32). After performing a leave‐one‐out analysis and excluding Harrion‐2019 and Harrion‐2024, heterogeneity decreased significantly. However, the treatment ranking remains unchanged. The P‐scores, which rank treatments based on effectiveness, were highest for Efruxifermin (p‐score = 0.95).

For the CINeMA framework, the certainty of evidence for Resmetirom and Efruxifermin indicated very low certainty with major concerns, especially regarding heterogeneity and imprecision, and some concerns due to within‐study bias and reporting bias (Supporting Information S4, Table S4.4).

#### 3.5.2 Percentage Change in LDL

**FIGURE 6:**
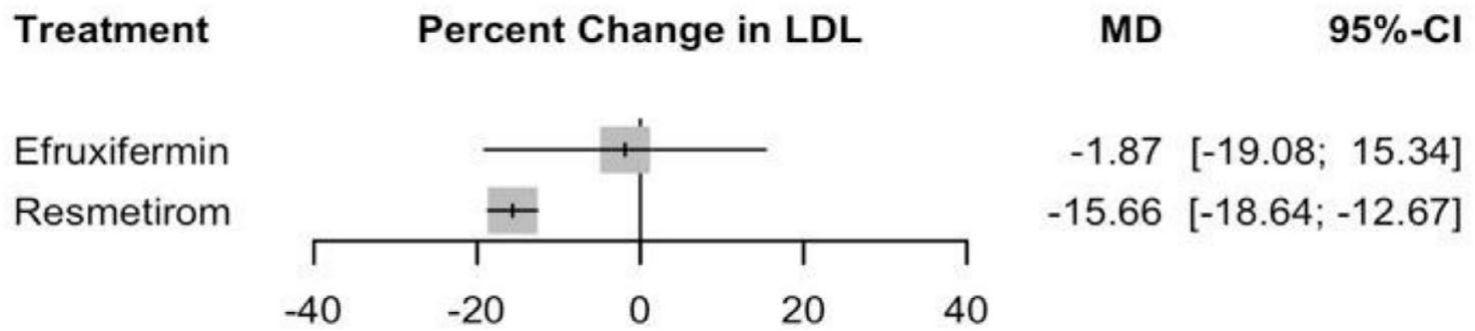
Network meta‐analysis results for percentage change in LDL.

Resmetirom represented the most significant effect in LDL levels with an MD of −15.66 (95% CI: −18.64 to −12.67, *p* = 0) compared to placebo. Efruxifermin showed a non‐significant effect with an MD of −1.87 (95% CI: −19.08 to 15.34, *p* = 0.83) (see Figure 6 and Supporting Information S7, Figure S7.5). In the indirect comparison between Efruxifermin and Resmetirom, no significant difference was observed, with an MD of 13.79% (95% CI: −3.68 to 31.25) (Supporting Information S6, Table S6.5), and the corresponding network plot is shown in Figure S8.5. No significant heterogeneity was detected, with *I* ^2^ = 0%, *τ* ^2^ = 0.00. Resmetirom was ranked highest based on its effectiveness with a p score of 0.96.

For the CINeMA framework, the certainty of evidence for the Resmetirom and Efruxifermin indicated low certainty with major concerns due to imprecision and some concerns due to reporting bias (Supporting Information S4, Table S4.5). The risk of bias was rated as low (*p* = 0.73).

#### 3.5.3 Percentage Change in HDL

**FIGURE 7:**
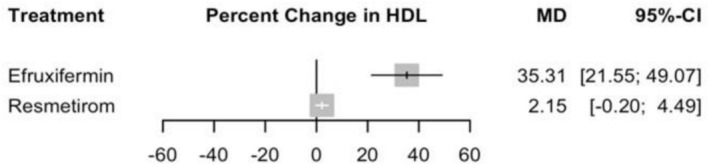
Network meta‐analysis results for percentage change in HDL.

Based on a random effects model assessing the percentage change in HDL, Efruxifermin was associated with a marked increase in HDL levels with an MD of 35.31 (95% CI: 21.55 to 49.07, *p* = 0.00) compared to placebo, and in the indirect comparison with Resmetirom, Efruxifermin also was associated with a significant increase in the HDL levels with an MD of 33.16% (95% CI: 19.20 to 47.12) (Supporting Information S6, Table S6.6), and the corresponding network plot is shown in Figure S8.6. Resmetirom showed a non‐significant increase with an MD of 2.15 (95% CI: −0.20 to 4.49, *p* = 0.07) (see Figure 7 and Supporting Information S7, Figure S7.6).

Regarding the heterogeneity analysis, no significant heterogeneity was detected, with *I* ^2^ = 0%, *τ* ^2^ = 0.00. The P‐scores, which rank treatments based on their effectiveness, were highest for Efruxifermin (P‐score = 1.00).

The certainty of evidence using the CINeMA framework, Resmetirom versus Efruxifermin, was moderate with some concerns due to reporting bias and imprecision (Supporting Information S4, Table S4.6). No evidence of publication bias.

### 3.6 Adverse Event Outcomes

#### 3.6.1 Serious Adverse Events

**FIGURE 8:**
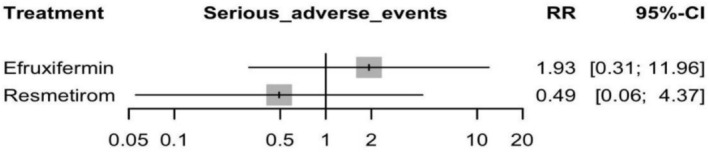
Network meta‐analysis results for serious adverse events.

Efruxifermin was associated with a non‐significant increase in the risk of SAEs with an RR of 1.93 (95% CI: 0.31 to 11.96, *p* = 0.48). Resmetirom also had a non‐significant decrease, with an RR of 0.49 (95% CI: 0.06 to 4.37, *p* = 0.52) (see Figure 8 and Supporting Information S7, Figure S7.7). There is no significant difference between Efruxifermin and Resmetirom, with an RR of 3.92 (95% CI: 0.23 to 67.45) (Supporting Information S6, Table S6.7), and the corresponding network plot is shown in Figure S8.7. The heterogeneity analysis showed moderate heterogeneity, with *I* ^2^ = 58.7%, *τ* ^2^ = 2.08, with significant heterogeneity within designs (*Q* = 12.12, df = 5, *p* = 0.03). BMI emerged as a statistical moderator with a possible effect on the estimates (*p* = 0.026). Despite conducting the leave‐one‐out analysis, the heterogeneity remains significant except when Harrison 2023_c was excluded; heterogeneity decreased with *I* ^2^ = 0%, and the treatment ranking remains the same. Conversely, excluding small sample size studies‐Harrison 2021_b, Harrison 2023_b, and Harrison 2025‐ decreases the heterogeneity *I* ^2^ = 0%, *τ* ^2^ = 0. Resmetirom was ranked the highest based on its effectiveness, with a *p*‐score of 0.78.

The certainty of evidence for Resmetirom vs. Efruxifermin was very low, due to major concerns related to imprecision and heterogeneity, and some concerns due to within‐study bias and reporting bias (Supporting Information S4, Table S4.7).

Regarding publication bias, significantly high concerns of bias were detected (*p* = 0.04).

#### 3.6.2 Treatment Discontinuation

**FIGURE 9:**
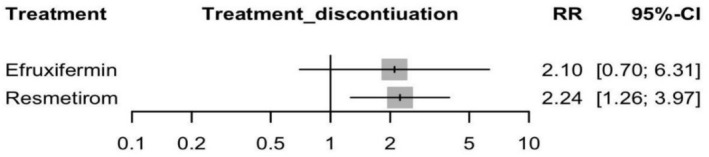
Network meta‐analysis results for treatment discontinuation.

Resmetirom showed a significant increase in the risk of discontinuation after random assessment with an RR of 2.24 (95% CI: 1.26 to 3.97, *p* = 0.005) compared to placebo. Efruxifermin showed a non‐significant increase in the risk of discontinuation with an RR of 2.10 (95% CI: 0.70 to 6.31, *p* = 0.18) (see Figure 9 and Supporting Information S7, Figure S7.8). The indirect comparison between Resmetirom and Efruxifermin with an RR of 0.94 (95% CI: 0.27 to 3.24), indicating no significant difference in the two treatment discontinuation rates (Supporting Information S6, Table S6.8), and the corresponding network plot is shown in Figure S8.8. The heterogeneity analysis revealed no significant heterogeneity, with *I* ^2^ = 0%, *τ* ^2^ = 0.00.

According to the CINeMA framework, the certainty of evidence in comparison between Resmetirom and Efruxifermin was low, due to within‐study bias, reporting bias, and imprecision (Supporting Information S4, Table S4.8).

#### 3.6.3 Nausea

**FIGURE 10:**
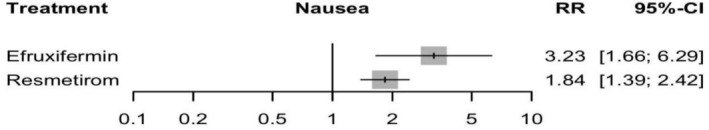
Network meta‐analysis results for nausea.

Efruxifermin significantly increased the risk of nausea, with an RR of 3.23 (95% CI: 1.66 to 6.29, *p* = 0.0006) compared to placebo. Resmetirom also showed an increased risk of nausea with an RR of 1.84 (95% CI: 1.39 to 2.42, *p* = 0.00) (see Figure 10 and Supporting Information S7, Figure S7.9). In the indirect comparison between Efruxifermin and Resmetirom, there is no significant difference, with an RR of 1.76 (95% CI: 0.85 to 3.62) (Supporting Information S6, Table S6.9), and the corresponding network plot is shown in Figure S8.9. The heterogeneity analysis revealed no significant heterogeneity, with *I* ^2^ = 0%, *τ* ^2^ = 0.00, indicating consistency among study results. The check of the transitivity assumption was valid, although there was a significant imbalance for study duration (*p* = 0.003).

The level of confidence in the certainty of evidence using the CINeMA model for the Resmetirom vs. Efruxifermin was ranked as moderate, due to some concerns with within‐study bias and reporting bias (Supporting Information S4, Table S4.9). There was a high level of concern regarding publication bias.

#### 3.6.4 Diarrhoea

**FIGURE 11:**
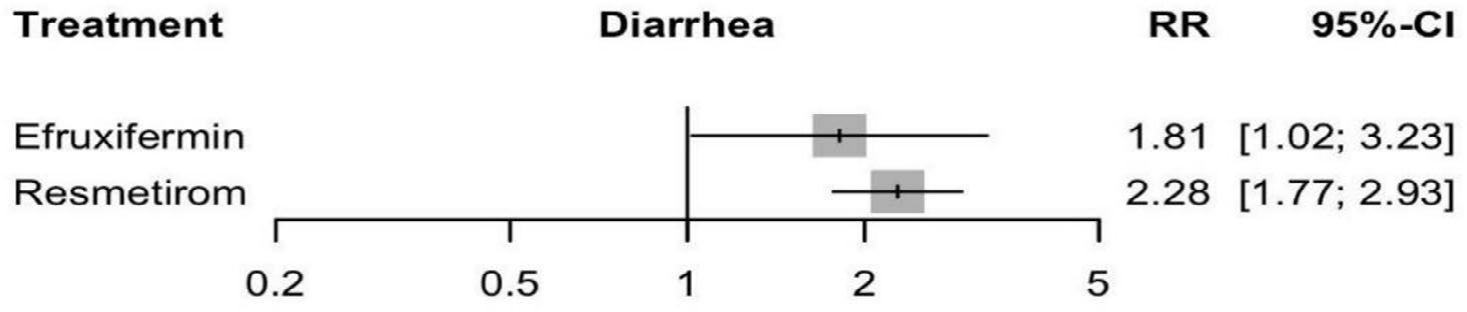
Network meta‐analysis results for diarrhoea.

Resmetirom and Efruxifermin were both associated with a significant increase in the risk of diarrhoea with an RR of 2.28 (95% CI: 1.77 to 2.93, *p* = 0.00) and 1.81 (95% CI: 1.02 to 3.23, *p* = 0.04), respectively (see Figure 11 and Supporting Information S7, Figure S7.10). There is no significant difference in the indirect comparison between Efruxifermin and Resmetirom, with an RR of 0.79 (95% CI: 0.42 to 1.49) (Supporting Information S6, Table S6.10), and the corresponding network plot is shown in Figure S8.10. Analysis showed low heterogeneity, with *I* ^2^ = 9.7%, *τ* ^2^ = 0.01, representing consistency among the study results.

Regarding the transitivity assumption validity, a significant imbalance was detected for study duration (*p* = 0.003). No evidence of publication bias among the included studies was detected (*p* = 0.13).

According to the CINeMA framework, the certainty of evidence for Resmetirom vs. Efruxifermin was ranked as moderate, due to some concerns about within‐study bias and reporting bias (Supporting Information S4, Table S4.10).

#### 3.6.5 Abdominal Pain

**FIGURE 12:**
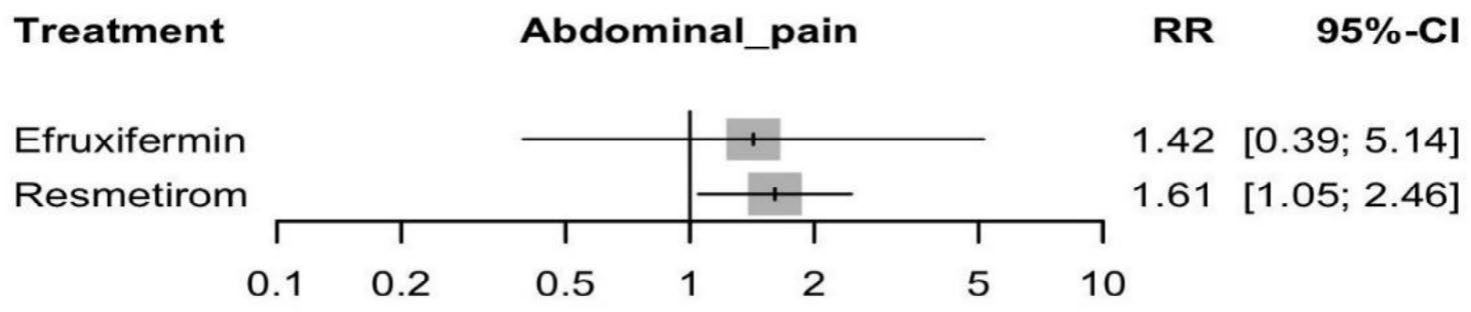
Network meta‐analysis results for abdominal pain.

Applying the random effects framework to assess the abdominal pain incidence showed a significant increase in Resmetirom group for abdominal pain with an RR of 1.61 (95% CI: 1.05 to 2.46, *p* = 0.03). While Efruxifermin showed non‐significant increase of abdominal pain with an RR of 1.42 (95% CI: 0.39 to 5.14, *p* = 0.58) (see Figure 12 and Supporting Information S7, Figure S7.11). In the indirect comparison between Efruxifermin and Resmetirom, there is no significant difference with an RR of 0.88 (95% CI: 0.23 to 3.43) (Supporting Information S6, Table S6.11), and the corresponding network plot is shown in Figure S8.11. No heterogeneity was detected between studies with *I* ^2^ = 0.0%, *τ* ^2^= 0, and non‐significant heterogeneity within designs (*Q* = 1.62, df = 2, *p* = 0.44).

According to the CINeMA framework, the certainty of evidence was rated as low for Resmetirom vs. Efruxifermin because of major concerns about imprecision and some concerns with within‐study bias and reporting bias (Supporting Information S4, Table S4.11).

There was little evidence of publication bias among the included studies (*p* = 0.51).

## 4 Discussion

In this systematic review and network meta‐analysis, 8 RCTs were included, aiming to comprehensively assess the effect of Resmetirom in comparison to Efruxifermin for MASH on hepatic steatosis and fibrosis, with particular focus on presenting the impact on liver enzymes, liver fat content, lipid profiles, and safety profile. Efruxifermin demonstrated substantial efficacy, particularly in reducing liver fat content (MRI‐PDFF), AST, ALT, and triglyceride levels. Resmetirom was associated with significant hepatic steatosis reduction, improving liver enzymes, and reducing LDL.

In regard to safety outcomes, Resmetirom was associated with higher risks of treatment discontinuation and increased risk of diarrhoea and abdominal pain; in contrast, Efruxifermin demonstrated a higher risk of nausea.

To our knowledge, no comprehensive meta‐analysis in the literature has investigated a head‐to‐head comparison between Resmetirom and Efruxifermin among patients with MASH, evaluating their impact on non‐invasive outcomes, biochemical markers, and metabolic endpoints, as well as their safety profiles. Importantly, the combination of MRI‐PDFF response and improvements in liver enzymes is clinically relevant, with higher odds of histological response or MASH resolution, which can serve as predictors of histological improvements [35, 36, 37]. Therefore, we conducted this systematic meta‐analysis to address this gap and provide a therapeutic comparison, highlighting the relevance of biochemical and non‐invasive imaging endpoints, along with metabolic and safety outcomes for clinical decision‐making.

Resmetirom (MGL‐3196) is a liver‐targeted, thyroid hormone receptor (THR)‐β‐selective drug [11]. Besides reducing liver steatosis and hepatic fibrogenesis, leading to improved lipid profile and reducing inflammation, Resmetirom induces iodothyronine deiodinase 1 (DIO1), which converts Thyroxine (T4) into the metabolically active free triiodothyronine (T3) in the liver [11]. The T3 then binds to its nuclear thyroid receptor β (THR‐β), which heterodimerizes with retinoid X receptor (RXR) to regulate carnitine palmitoyltransferase I (CPT1) in hepatocytes [11]. This enzyme plays a key role in hepatic de novo lipogenesis by controlling mitochondrial fatty acid oxidation (FAO), and Sterol regulatory element binding transcription factor 1 (SREBF1) leads to a rapid decrease and robust lowering of non‐HDL‐C and hepatic triglyceride accumulation, consistent with our network findings [11, 38, 39].

Efruxifermin, a bivalent Fc‐fibroblast growth factor 21 (FGF21) analogue, FGF21 has been recognized as important for the control of glucose and lipid metabolism, leading to reduced dyslipidemia, body weight, and hepatic steatosis, besides increasing insulin sensitivity [12]. In addition, Efruxifermin showed promising results, including improvement in non‐invasive markers of liver injury such as AST and ALT, as well as improvements in fibrosis, aligned with our network results [12, 13, 40, 41].

The management of patients with MASH consists of a multimodal intervention that covers multiple aspects, like weight reduction, lifestyle modifications, and pharmacological intervention [42]. Decreasing MASH‐related mortality, resolution of steatohepatitis without worsening of fibrosis, or regression of fibrosis without worsening of steatohepatitis, and more recently, a combined resolution of steatohepatitis and regression of fibrosis, as well, reducing progression to cirrhosis or HCC, are considered the mainstay for assessing treatment efficacy [5, 43].

The phase 3 trial MAESTRO‐ NASH showed the efficacy of Resmetirom 100 mg on both MASH resolution without fibrosis impairment and ≥ 1 stage improvement in fibrosis without MASH impairment, compared to placebo, for 52 weeks [21]. Based on these results and a surrogate clinical endpoint, the Food and Drug Administration (FDA) approved Resmetirom for MASLD patients with F2–F3 fibrosis [21, 44].

The findings of our study revealed that Efruxifermin has a superior hepatic response through hepatic fat reduction and improving hepatic enzymes and lipid profile, such as HDL and TG. Our results align with a recent meta‐analysis that supports Efruxifermin's potential as a multifaceted and effective treatment for MASH. Although Efruxifermin is not yet approved by the FDA for the treatment of MASH, Efruxifermin's biochemical and metabolic enhancements, alongside its reassuring safety profile, suggest its potential to become a cornerstone in the future clinical guidelines and treatment strategies of MASH, offering a more comprehensive approach to combating the disease [13, 45, 46].

The strength of our study lies in addressing one‐to‐one treatment comparisons using only RCTs, the gold standard for evaluating the effectiveness of an intervention. It must be mentioned that in this network meta‐analysis, we had some limitations: firstly, the number of participants in some RCTs was small and relatively short study duration, also the heterogeneity among trials—such as differences in inclusion criteria, dosing regimens, and follow‐up duration; secondly, although using the network meta‐analysis structure allowed us to generate comparative effects in the absence of direct head‐to‐head evidence, this structure relies on indirect comparisons, which potentially introduces uncertainty into the estimated treatment effects with misleading ranking. Finally, a significant heterogeneity was found in the measurement of change in ALT, AST, triglycerides, and serious adverse events. This could be clarified by variation in the patient population, disease severity, and lack of standardization through the studies on medication dosage or duration of treatment.

Most comparisons between treatments were based on indirect evidence; therefore, these data inform the need for one‐to‐one comparison between different treatment methods in future clinical trials to validate the promising results in the literature, which may help the development of combination therapies and decision‐making process by providing evidence‐based data to guide patient treatment based on individual patient needs and health conditions [10, 47].

## 5 Conclusion

Using network meta‐analysis, we observed that Resmetirom and Efruxifermin are superior to placebo and comparable to one another in improving key biochemical and non‐invasive markers. Large comparative RCTs, particularly head‐to‐head trials with longer duration, are warranted to further establish the comparative efficacy of different interventions for MASH.

## Author Contributions

**Thekrayat Asad:** conceptualization, resources, writing – review and editing, data curation. **Doha Jaber:** conceptualization, methodology, writing – original draft, writing – review and editing, resources, project administration, visualization, supervision, validation, investigation, data curation. **Hazem Ayesh:** conceptualization, methodology, software, formal analysis, writing – original draft, writing – review and editing, project administration. **Ayah Abu Lehia:** conceptualization, data curation, resources, writing – review and editing. **Inas Jaber:** conceptualization, data curation, investigation, resources, writing – review and editing.

## Funding

The authors have nothing to report.

## Disclosure

Permission to reproduce material from other sources: Not applicable; this article is a meta‐analysis of previously published clinical trials that are appropriately cited.

## Ethics Statement

This article is a meta‐analysis of previously published clinical trials; it does not contain any new studies with human or animal participants. Therefore, ethics approval and informed consent were not required.

## Consent

The authors have nothing to report.

## Conflicts of Interest

The authors declare no conflicts of interest.

## Supporting information


**Data S1:** edm270218‐sup‐0001‐SupinfoS1.docx.

**Data S2:** edm270218‐sup‐0002‐SupinfoS2.docx.

## Data Availability

Original data generated and analyzed during this study are included in this published article or in the data repositories listed in References. Supporting Information contains additional data.
