# Supplementary material for: Comparative Efficacy and Safety of Resmetirom and Efruxifermin for Metabolic Dysfunction‐Associated Steatohepatitis: A Network Meta‐Analysis of Randomized Controlled Trials
Source: Endocrinol Diabetes Metab. 2026 Apr 7;9(3):e70218. doi: 10.1002/edm2.70218 (PMC13056698; doi:10.1002/edm2.70218)
Supplement: Supplementary file 1 — Data S1: edm270218‐sup‐0001‐SupinfoS1.docx. [file EDM2-9-e70218-s002.docx]

Supplementary 12: S12: Risk of bias of individual studies.

| **Study ID** | **Domain** | **Assessment** | **Explanation** |
| --- | --- | --- | --- |
| **Harrison,2019** | Random Sequence Generation | Low risk | A computer-generated simple randomisation schedule |
|  | Allocation Concealment | Low risk | A computer-generated simple randomisation schedule prepared by study administrators was used to randomly assign patients (2:1) |
|  | Blinding of Participants/Personnel | Low risk | Patients, the sponsor, investigators, and site personnel involved with dispensing study medication, carrying out study procedures, evaluating patients, entering study data, or evaluating study data were masked to treatment assignment throughout the study |
|  | Blinding of Outcome Assessment | Low risk | Patients, the sponsor, investigators, and site personnel involved with dispensing study medication, carrying out study procedures, evaluating patients, entering study data, or evaluating study data were masked to treatment assignment throughout the study |
|  | Incomplete Outcome Data | Low risk | The enrolment size was designed to allow for 10% dropout before the week 12 visit, and as such, patients who dropped out of the study would not be replaced. Additionally, the sample size was expected to provide meaningful liver biopsy-related data. |
|  | Selective Reporting | Low risk | All endpoints, except where otherwise specified as post hoc (the only post-hoc analysis included the dose comparison), were prespecified in the statistical analysis plan, which was finalised and signedbefore the week 12 interim (if relevant) or study unblinding after all patients completed the 36-week study. |
|  | Other Bias | None |  |
|  | **Overall Judgment** | **Low risk** |  |
| **Harrison,2021_a** | Random Sequence Generation | Low risk | The main was a 36-week multicenter, randomized, double-blind, placebo-controlled, and this study (OLE study) is conducted as an extension to it. |
|  | Allocation Concealment | High risk | Based on a trough and 4-hour post-dose pharmacokinetic assessment at week 2, patients remained on the initial dose or were down-titrated or up-titrated by 20 mg at week 4, as determined by an unblinded reviewer. |
|  | Blinding of Participants/Personnel | High risk | Treatment and dose were blinded at the time of entry into the OLE study, and an unblinded reviewer assigned dose until the main study was completed and had been unblinded. After unblinding of the main study and reviewing the data, all patients in the OLE study had a dose increase to at least 80 mg, the last increase in dose occurring at OLE week 24 |
|  | Blinding of Outcome Assessment | Some concerns | After the main study was unblinded, all patients in the OLE study had doses increased to at least 80 mg or 100 mg of resmetirom (the patients most advanced in the OLE study had the dose increase no later than week 24). |
|  | Incomplete Outcome Data | Low risk | Twenty nine of the 31 patients completed all 36 weeks of the OLE study; 2 discontinuations were patient decision. |
|  | Selective Reporting | Low risk | During the main 36-week study, a protocol amendment was completed to allow patients to enroll in a 36-week active treatment OLE study in which all patients received open-label resmetirom treatment, and safety, serial imaging, and biomarker assessments were conducted. |
|  | Other Bias | None |  |
|  | **Overall Judgment** | **High risk** |  |
| **Harrison,2021_b** | Random Sequence Generation | Low risk | This multicenter, randomized, double-blind, placebo-controlled, parallel-group. |
|  | Allocation Concealment | Some concerns | The principal investigators enrolled participants. |
|  | Blinding of Participants/Personnel | Low risk | Quadruple Masking (Participant, Care Provider, Investigator, Outcomes Assessor) |
|  | Blinding of Outcome Assessment | Low risk | Investigators, radiologists, pathologist, staff, patients, sponsor and medical monitor remained masked to treatment groups during the study. |
|  | Incomplete Outcome Data | Some concerns | Multiple imputation was employed for missing primary efficacy values in the FAS, Three secondary endpoint responder analyses were performed. MRI-PDFF responders in the FAS were calculated with missing values imputed as non-responders and in the MRI–PDFF (HFF) evaluable analysis set (MAS, the subgroup with baseline and week 12 MRI–PDFF assessments) without imputation.  NAS responders in the liver biopsy evaluable analysis set (BAS, MRI–PDFF  responders with liver biopsy at baseline and after treatment) were calculated  without imputation. |
|  | Selective Reporting | Low risk | All pre-specified outcomes were reported. |
|  | Other Bias |  | The study was funded by Akero Therapeutics, which, in collaboration with the authors, was involved in study design and data collection. |
|  | **Overall Judgment** | **Some concerns** |  |
| **Harrison,2023_a** | Random Sequence Generation | Low risk | MAESTRO-NAFLD-1 was a randomized, DB, placebo-controlled phase 3 trial evaluating the safety and tolerability of resmetirom in patients with NAFLD (presumed NASH). |
|  | Allocation Concealment | Low risk | Patients and study personnel administering the study drug and performing the clinical assessments were blinded to the individual patient’s treatment (resmetirom or placebo). |
|  | Blinding of Participants/Personnel | Low risk | Quadruple Masking (Participant, Care Provider, Investigator, Outcomes Assessor) |
|  | Blinding of Outcome Assessment | Low risk | Quadruple Masking (Participant, Care Provider, Investigator, Outcomes Assessor) |
|  | Incomplete Outcome Data | Low risk | This study was impacted by COVID-19 and the estimand in the statistical analysis plan reflected the statistical approach taken to address COVID-19-related missing data, but they handle the missing data by imputation , The imputation was conducted in two stages, where stage 1 utilized a single imputation using a patient’s own data and stage 2 used a multiple imputation approach. In stage 1, invalid and missing data caused by the COVID-19 pandemic were imputed using the ‘valid visit’ lipid measurement (if available) obtained just before the missing visit. |
|  | Selective Reporting | Low risk | All pre-specified outcomes were reported. |
|  | Other Bias | None |  |
|  | **Overall Judgment** | **Low risk** |  |
| **Harrison,2023_b** **Supplementary** | Random Sequence Generation | Low risk | Cohort C was a randomized, double-blind, placebo-controlled phase IIa expansion cohort of the BALANCED study (NCT03976401) that evaluated safety and tolerability of efruxifermin in patients with NASH and compensated cirrhosis (F4 fibrosis). |
|  | Allocation Concealment | Low risk | Interactive response technology (IRT) was used for centralized randomization and treatment assignment. |
|  | Blinding of Participants/Personnel | Low risk | Quadruple Masking (Participant, Care Provider, Investigator, Outcomes Assessor) |
|  | Blinding of Outcome Assessment | Low risk | Quadruple Masking (Participant, Care Provider, Investigator, Outcomes Assessor) |
|  | Incomplete Outcome Data | Some concerns | Missing values were imputed using the last-observed-carried-forward (LOCF) method, but due to small number of participants this increase the risk |
|  | Selective Reporting | Low risk | All pre-specified outcomes were reported. |
|  | Other Bias | None |  |
|  | **Overall Judgment** | **Some concerns** |  |
| **Harrison,2023_c** | Random Sequence Generation | Low risk | HARMONY is a 96-week multicentre, randomised,double-blind, placebo-controlled, parallel-group, Patient randomisation was performed by an interactive response technology (IRT) system (Endpoint Clinical, Wakefield, MA, USA). |
|  | Allocation Concealment | Low risk | Site personnel obtained the patient’s identification number and blinded study drug assignment from the IRT. |
|  | Blinding of Participants/Personnel | Low risk | Patients, investigators, pathologists, site staff, and the sponsor remained masked to group assignments during the course of the study. |
|  | Blinding of Outcome Assessment | Low risk | The prespecified primary efficacy analysis was done in the LBAS to estimate treatment effect under ideal conditions (completer analysis). |
|  | Incomplete Outcome Data | Some concerns | All biomarkers, including non-invasive markers of fibrosis, were assessed in the FAS using data from patients with non-missing values, without performing any imputations. |
|  | Selective Reporting | Low risk | The prespecified primary efficacy analysis was done in the LBAS to estimate treatment effect under ideal conditions (completer analysis). |
|  | Other Bias | Some concerns | The funder of the study had a role in the study design, data collection, data analysis, data interpretation, and writing of the report. |
|  | **Overall Judgment** | **Some concerns** |  |
| Harrison,2024 | Random Sequence Generation | Low risk | This is a multicenter, double-blind, randomized, placebo-controlled study. |
|  | Allocation Concealment | Low risk | Randomization was performed with the use of an interactive Web response system. |
|  | Blinding of Participants/Personnel | Low risk | double-blind |
|  | Blinding of Outcome Assessment | Some concerns | There will be 2 interim analyses of the final primary endpoint (time to experiencing an adjudicated Composite Clinical Outcome event). All interim analyses will be performed by an unblinded team, not otherwise involved in the management of the study, and overseen by the DMC. |
|  | Incomplete Outcome Data | Some concerns | Patients with missing biopsies were considered to have not had a response, A total of 11 of 966 patients had a delay in their week 52 biopsy for reasons related to coronavirus disease 2019 (Covid-19), were considered to have missing data completely at random, and were removed from the primary biopsy analysis population |
|  | Selective Reporting | Low risk | Details of primary, secondary, exploratory, and safety analyses were specified in the statistical analysis plan. |
|  | Other Bias | None |  |
|  | **Overall Judgment** | **Some concerns** |  |
| **Harrison,2025** | Random Sequence Generation | Low risk | Cohort D (AK-US-001-0103) was multicenter, randomized, double-blind, placebo-controlled, parallel-group. |
|  | Allocation Concealment | Low risk | An interactive response system was used to randomly assign patients (2:1) to the efruxifermin 50 mg or placebo group. |
|  | Blinding of Participants/Personnel | Low risk | Quadruple Masking (Participant, Care Provider, Investigator, Outcomes Assessor) |
|  | Blinding of Outcome Assessment | Some concerns | Quadruple Masking (Participant, Care Provider, Investigator, Outcomes Assessor) |
|  | Incomplete Outcome Data | Low risk | The mean change in HFF with efruxifermin treatment may have been affected by data missing for 5 patients due to 2 discontinuations and 3 baseline measurements being excluded because of invalid data collection.All other biomarkers, including noninvasive markers of fibrosis, were assessed in the FAS using data from patients with non-missing values,without performing any imputations. |
|  | Selective Reporting | Low risk | This article describes results of the primary (safety and tolerability of efruxifermin) and secondary (markers of liver health and glucose and lipid metabolism) endpoints. |
|  | Other Bias | Some concerns | Conflicts of interest: The authors disclose the following: Stephen A. Harrison is involved in clinical trials, receives support for attending meetings or travel, and serves on advisory boards of Akero Therapeutics, Inc. Juan P.Frias has received a research grant through his institution and receives consulting fees from Akero Therapeutics,etc. |
|  | **Overall Judgment** | **Some concerns** |  |
