# Supplementary material for: Comparative Efficacy and Safety of Resmetirom and Efruxifermin for Metabolic Dysfunction‐Associated Steatohepatitis: A Network Meta‐Analysis of Randomized Controlled Trials
Source: Endocrinol Diabetes Metab. 2026 Apr 7;9(3):e70218. doi: 10.1002/edm2.70218 (PMC13056698; doi:10.1002/edm2.70218)
Supplement: Supplementary file 2 — Data S2: edm270218‐sup‐0002‐SupinfoS2.docx. [file EDM2-9-e70218-s001.docx]

Contents

[Supplement 1. Search strategy 1](#_Toc223812506)

[Supplement S2. Baseline characteristics of the included studies 3](#_Toc223812507)

[Supplement S3. Baseline Characteristics of the Participants 6](#_Toc223812508)

[Suplement S4. Risk of bias assessment of included trials for each outcome 7](#_Toc223812509)

[Table S4.1: Certainty of evidence for percentage change in MRI-PDFF 7](#_Toc223812510)

[Table S4.2: Certainty of evidence for change in ALT 8](#_Toc223812511)

[Table S4.3: Certainty of evidence for change in AST 8](#_Toc223812512)

[Table S4.4: Certainty of evidence for percentage change in Triglyceride 10](#_Toc223812513)

[Table S4.5: Certainty of evidence for percentage change in LDL 10](#_Toc223812514)

[Table S4.6: Certainty of evidence for percentage change in HDL 11](#_Toc223812515)

[Table S4.7: Certainty of evidence for Serious adverse events 11](#_Toc223812516)

[Table S4.8: Certainty of evidence for treatment discontinuation 11](#_Toc223812517)

[Table S4.9: Certainty of evidence for Nausea 12](#_Toc223812518)

[Table S4.10: Certainty of evidence for diarrhea 13](#_Toc223812519)

[Table S4.11: Certainty of evidence for Abdominal pain 13](#_Toc223812520)

[Supplement S5. Publication bias (funnel plot) 14](#_Toc223812521)

[Figure S5.1 Percentage Change in MRI-PDFF 14](#_Toc223812522)

[Figure S5.2 Change in ALT 15](#_Toc223812523)

[Figure S5.3 Change in AST 15](#_Toc223812524)

[Figure S5.4 Percentage Change in Triglyceride 16](#_Toc223812525)

[Figure S5.5 Percentage Change in LDL 16](#_Toc223812526)

[Figure S5.6 Percentage Change in HDL 17](#_Toc223812527)

[Figure S5.7 Serious adverse events 17](#_Toc223812528)

[Figure S5.8 Treatment Discontinuation 18](#_Toc223812529)

[Figure S5.9 Nausea 18](#_Toc223812530)

[Figure S5.10 Diarrhea 19](#_Toc223812531)

[Figure S5.11 Abdominal pain 19](#_Toc223812532)

[Supplement 6: Network meta-analysis results (league) 20](#_Toc223812533)

[Table 6.1. Network meta-analysis results for Percentage change in MRI-PDFF 20](#_Toc223812534)

[Table 6.2. Network meta-analysis results for change in ALT 20](#_Toc223812535)

[Table 6.3. Network meta-analysis results for change in AST 21](#_Toc223812536)

[Table 6.4. Network meta-analysis results for Percentage change in Triglyceride 21](#_Toc223812537)

[Table 6.5. Network meta-analysis results for Percentage Change in LDL 21](#_Toc223812538)

[Table 6.6 Network meta-analysis results for Percentage change in HDL 22](#_Toc223812539)

[Table 6.7. Network meta-analysis results for serious adverse events 22](#_Toc223812540)

[Table 6.8. Network meta-analysis results for Treatment Discontinuation 22](#_Toc223812541)

[Table 6.9. Network meta-analysis results for Nausea 23](#_Toc223812542)

[Table 6.10. Network meta-analysis results for Diarrhea 23](#_Toc223812543)

[Table 6.11. Network meta-analysis results for Abdominal pain 24](#_Toc223812544)

[Supplement 7: Network meta-analysis results for each outcome 25](#_Toc223812545)

[Figure S7.1: Network meta-analysis results for Percent change in MRI-PDFF 25](#_Toc223812546)

[Figure S7.2: Network meta-analysis results for change in ALT 25](#_Toc223812547)

[Figure S7.3: Network meta-analysis results for change in AST 26](#_Toc223812548)

[Figure S7.4: Network meta-analysis results for percentage change in triglyceride 26](#_Toc223812549)

[Figure S7.5: Network meta-analysis results for percentage change in LDL 27](#_Toc223812550)

[Figure S7.6: Network meta-analysis results for percentage change in HDL 27](#_Toc223812551)

[Figure S7.7: Network meta-analysis results for serious adverse events 28](#_Toc223812552)

[Figure S7.8: Network meta-analysis results for Treatment Discontinuation 28](#_Toc223812553)

[Figure S7.9: Network meta-analysis results for Nausea 29](#_Toc223812554)

[Figure S7.10: Network meta-analysis results for Diarrhea 29](#_Toc223812555)

[Figure S7.11: Network meta-analysis results for Abdominal pain 30](#_Toc223812556)

[Supplement S8. Network plots of treatment comparisons 31](#_Toc223812557)

[Figure S8.1 Network Plot of Treatment Comparisons for Percent change in MRI-PDFF 31](#_Toc223812558)

[Figure S8.2 Network Plot of Treatment Comparisons for change in ALT 32](#_Toc223812559)

[Figure S8.3 Network Plot of Treatment Comparisons for change in AST 32](#_Toc223812560)

[Figure S8.4 Network Plot of Treatment Comparisons for Percent change in Triglyceride 33](#_Toc223812561)

[Figure S8.5 Network Plot of Treatment Comparisons for Percent change in LDL 33](#_Toc223812562)

[Figure S8.6 Network Plot of Treatment Comparisons for Percent change in HDL 34](#_Toc223812563)

[Figure S8.7 Network Plot of Treatment Comparisons for Serious Adverse Events 35](#_Toc223812564)

[Figure S8.8 Network Plot of Treatment Comparisons for Treatment Discontinuation 36](#_Toc223812565)

[Figure S8.9 Network Plot of Treatment Comparisons for Nausea 37](#_Toc223812566)

[Figure S8.10 Network Plot of Treatment Comparisons for Diarrhea 38](#_Toc223812567)

[Figure S8.11 Network Plot of Treatment Comparisons for Abdominal Pain 39](#_Toc223812568)

[Supplement S9: Summary of the excluded studies: 40](#_Toc223812569)

[Supplement S10: Meta-regression: 41](#_Toc223812570)

[Figure S10.2 Percentage Change in MRI-PDFF 41](#_Toc223812571)

[Figure S10.3 Change in ALT 44](#_Toc223812572)

[Figure S10.4 Change in AST 46](#_Toc223812573)

[Figure S10.5 Percentage Change in Triglyceride 49](#_Toc223812574)

[Figure S10.6 Percentage Change in LDL 51](#_Toc223812575)

[Figure S10.7 Percentage Change in HDL 53](#_Toc223812576)

[Figure S10.8 Serious adverse events 55](#_Toc223812577)

[Figure S10.9 Treatment Discontinuation 57](#_Toc223812578)

[Figure S10.10 Nausea 59](#_Toc223812579)

[Figure S10.11 Diarrhea 61](#_Toc223812580)

[Figure S10.12 Abdominal pain 63](#_Toc223812581)

[Supplement S11: PRISMA Checklist 65](#_Toc223812582)

# Supplement 1. Search strategy

Search strategy from inception to January 25, 2025.

Pubmed

(((MASH OR (Metabolic dysfunction-associated steatohepatitis) OR (NASH OR Nonalcoholic Steatohepatitis OR nonalcoholic fatty liver disease OR NAFLD OR Metabolic Dysfunction-Associated Steatotic Liver Disease OR MASLD)) AND ((Resmetirom OR MGL-3196) OR (thyroid hormone receptor-β agonist)) OR (Efruxifermin OR AKR-001)) AND (RCT OR trial OR (Randomized Controlled Trial)))

Scopus

((MASH OR "Metabolic dysfunction-associated steatohepatitis" OR "Nonalcoholic Steatohepatitis" OR NASH OR "nonalcoholic fatty liver disease" OR NAFLD OR "Metabolic Dysfunction-Associated Steatotic Liver Disease" OR MASLD) AND (Resmetirom OR MGL-3196 OR "thyroid hormone receptor-β agonist" OR Efruxifermin) AND ("Randomized Controlled Trial" OR RCT OR "placebo-controlled" OR "double-blind trial" OR "comparative study")AND ("liver enzymes" OR ALT OR AST OR "MRI-PDFF" OR "liver fat fraction" OR "NASH resolution" OR "liver histology" OR "metabolic parameters" OR "lipid profile"))AND NOT ("case report" OR "review article" OR "pediatric" OR "non-human" OR "in vitro")

Cochrane CENTRAL

((MASH OR "Metabolic dysfunction-associated steatohepatitis" OR "Nonalcoholic Steatohepatitis" OR NASH OR "nonalcoholic fatty liver disease" OR NAFLD OR "Metabolic Dysfunction-Associated Steatotic Liver Disease" OR MASLD) AND (Resmetirom OR MGL-3196 OR "thyroid hormone receptor-β agonist" OR Efruxifermin) AND ("Randomized Controlled Trial" OR RCT OR "placebo-controlled" OR "double-blind trial" OR "comparative study")AND ("liver enzymes" OR ALT OR AST OR "MRI-PDFF" OR "liver fat fraction" OR "NASH resolution" OR "liver histology" OR "metabolic parameters" OR "lipid profile"))AND NOT ("case report" OR "review article" OR "pediatric" OR "non-human" OR "in vitro")

# Supplement S2. Baseline characteristics of the included studies

Table 1 S2: Study Characteristics and Outcomes in the Included Clinical Trials

The table summarizes various MASH clinical trials, detailing study design, registration, duration, treatment arms, and population characteristics. Abbreviations used include RCT (randomized controlled trial), DB (double-blind), PC (placebo-controlled), NAFLD (non-alcoholic fatty liver disease), NASH (non-alcoholic steatohepatitis), and MRI-PDFF (magnetic resonance imaging-proton density fat fraction).

| Study | Trial Phase | Year | Registration | Intervention | Comparator | Duration | Population |
| --- | --- | --- | --- | --- | --- | --- | --- |
| Harrison,2019 | phase 2 | 2019 | NCT02912260 | Resmetirom 80 mg | Placebo | 36 weeks | Adults with biopsy-confirmed non-alcoholic steatohepatitis (NASH) and liver fibrosis stages F1 to F3. -Hepatic fat fraction of at least 10% at baseline, assessed by MRI-proton density fat fraction (MRI-PDFF). |
| Harrison,2021_a | Phase 2 | 2021 | NCT03900429 | (Res/Res) Resmetirom 80 mg or Resmetirom 100 mg after receiving Resmetriom in main trial | Pbo/Res)Resmetirom 80 mg or Resmetirom 100 mg after receiving Resmetriom in main trial | 36 weeks | Adults with biopsy-confirmed nonalcoholic steatohepatitis (NASH) who had persistently mild to markedly elevated liver enzymes at the end of the main study |
| Harrison,2021_b | phase 2a | 2021 | NCT03976401 | Efruxifermin 28 mg or  Efruxifermin 50 mg or  Efruxifermin 70 mg | Placebo | 16 weeks | Patients were adults with biopsy-proven NASH, fibrosis stage 1–3, NAS of ≥4 (with at least a score of 1 in each of steatosis, ballooning degeneration and lobular infammation), HFF of ≥10% by MRI–PDFF and FibroScan measurement >7.0 kPa. |
| Harrison,2023_a | phase 3 | 2023 | NCT04197479) | Resmetirom 100 mg open label, Resmetirom 100 mg double blind, Resmetirom 80 mg double blind | Placebo | 64 weeks | Adults with biopsy-confirmed nonalcoholic fatty liver disease (NAFLD) and presumed nonalcoholic steatohepatitis (NASH) |
| Harrison,2023_b | phase 2a | 2023 | NCT03976401 | Efruxifermin 50 mg | placebo | 26 weeks | Adults with biopsy-confirmed non-alcoholic steatohepatitis (NASH) and compensated cirrhosis (stage 4 fibrosis). |
| Harrison,2023_c | phase 2b | 2023 | NCT04767529 | Efruxifermin 28 mg or  Efruxifermin 50 mg | placebo | 96 weeks | Adults with biopsy-confirmed NASH, defined by a non-alcoholic fatty liver disease activity score (NAS) of 4 or higher and scores of 1 or higher in each of steatosis, ballooning, and lobular inflammation,with histological stage F2 or F3 fibrosis. |
| Harrison,2024 | Phase 3 | 2024 | NCT03900429 | Resmetirom 100 mg, Resmetirom 80 mg | placebo | 52 weeks | Adults who are 18 years of age or older. -A total of 1050 patients underwent randomization; 966 patients who had a fibrosis stage of F1B, F2, or F3 at baseline (primary population for safety and efficacy) |
| Harrison,2025 | Phase 2 | 2025 | NCT05039450 | Efruxifermin 50 mg | placebo | 12 weeks | Adults with T2D and MASH with fibrosis (F1–F3) on stable GLP-1RA therapy |

# Supplement S3. Baseline Characteristics of the Participants

Table S3. Baseline Characteristics of Patients

The table summarizes the baseline characteristics of patients included in the study. Characteristics include age, male participants, body mass index (BMI), glycated hemoglobin (HbA1c), Hypertension, levels of alanine aminotransferase (ALT), aspartate aminotransferase (AST), MRI-PDFF, and lipid profiles. Mean values with SD are provided for continuous variables, while percentages are provided for categorical variables.

| Study ID | Age (mean, SD) | Male (%) | HbA1c (%) | BMI (Mean ± SD) | Diabetes % | Hypertension % | Baseline ALT (Mean ± SD) | Baseline AST (Mean ± SD) | Baseline MRI-PDFF (% ± SD ) | Baseline Triglycerides (Mean ± SD) | Baseline LDL (Mean ± SD) | Baseline HDL (Mean ± SD) | Baseline Total Cholesterol (Mean ± SD) |
| --- | --- | --- | --- | --- | --- | --- | --- | --- | --- | --- | --- | --- | --- |
| Harrison,2019 | 50.32±11 | 50% | 6.25±1.1 | 35.08±6.14 | 39.20% | 36.14±52.03 | 53.31±30.46 | 36.05±18.7 | 20±7.26 | 172.79±80.23 | 113.14±30.26 | 44.26±12.76 | 194.77±38.59 |
| Harrison,2021_a | 48.2±12.3 | 51.60% | NR | 35.3±5.2 | 45.20% | 16±51.6 | 64±43.2 | 42.5±20.3 | 19.4±7.1 | 177.4±88.8 | 123.7±39.1 | 45.1±12.6 | NR |
| Harrison,2021_b | 52.12±12.24 | 42.50% | 6.34±1.1 | 37.56±6.67 | 51.25% | NR | 55.7±31.36 | 39.89±20.1 | 19.57±6.88 | 185.67±99.88 | 108.65±36.1 | 42.28±10.31 | 188.04±43.98 |
| Harrison,2023_a | 55.88±11.77 | 43.60% | NR | 35.42±6.1 | 55.80% | 226.69±87.6 | 37.03±26.24 | 25.65±14.96 | 17.87±6.98 | 180.1±100.6 | 110.12±37.74 | 43.77±13.9 | 179.88±44.3 |
| Harrison,2023_b | 59.76±11.56 | 36.70% | 6.3±1.15 | 37±6.6 | 50% | NR | 32±17.6 | 30.6±16.2 | NR | 130.3±61.03 | 89.9±33.9 | 48.03±13.6 | 163.96±40.8 |
| Harrison,2023_c | 54.7±10.4 | 38% | 6.8±1.1 | 38±7 | 70.30% | NR | 58.5±34.3 | 50.5±33.4 | 17.7±6.5 | 160.7±70 | 100.4±33.7 | 41.5±8.8 | NR |
| Harrison,2024 | 56.6±10.9 | 43.80% | NR | 35.7±6.8 | 67.20% | 251.3±78.24 | 54.6±32.2 | 40.5±23.23 | 17.7±6.74 | NR | 105.5±38.5 | NR | NR |
| Harrison,2025 | 57.7±NR | 42% | 6.8±NR | 35.13±NR | NR | NR | 33.7±NR | 25.4±NR | 11.9±NR | 165.58±NR | 81.1±NR | 44.6±NR | NR |

# Suplement S4. Risk of bias assessment of included trials for each outcome

## Table S4.1: Certainty of evidence for percentage change in MRI-PDFF

| Comparison | Within_Study_Bias | Reporting_Bias | Indirectness | Imprecision | Heterogeneity | Incoherence | Overall_Confidence | Reason_for_downgrade |
| --- | --- | --- | --- | --- | --- | --- | --- | --- |
| Placebo:Resmetirom | No concerns | Some concerns | No concerns | Some concerns | Major concerns | No concerns | Low | Reporting bias, Imprecision, Heterogeneity |
| Efruxifermin:Placebo | Some concerns | Some concerns | Some concerns | Some concerns | Major concerns | No concerns | Low | Within-study bias, Reporting bias, Indirectness, Imprecision, Heterogeneity |
| Efruxifermin:Resmetirom | Some concerns | Some concerns | No concerns | Some concerns | Major concerns | No concerns | Low | Within-study bias, Reporting bias, Imprecision, Heterogeneity |

## Table S4.2: Certainty of evidence for change in ALT

##

| Comparison | Within_Study_Bias | Reporting_Bias | Indirectness | Imprecision | Heterogeneity | Incoherence | Overall_Confidence | Reason_for_downgrade |
| --- | --- | --- | --- | --- | --- | --- | --- | --- |
| Placebo:Resmetirom | Some concerns | Some concerns | No concerns | Major concerns | Major concerns | No concerns | Very low | Within-study bias, Reporting bias, Imprecision, Heterogeneity |
| Efruxifermin:Placebo | Some concerns | Some concerns | No concerns | Major concerns | Major concerns | No concerns | Very low | Within-study bias, Reporting bias, Imprecision, Heterogeneity |
| Efruxifermin:Resmetirom | Some concerns | Some concerns | No concerns | Major concerns | Major concerns | No concerns | Very low | Within-study bias, Reporting bias, Imprecision, Heterogeneity |

## Table S4.3: Certainty of evidence for change in AST

| Comparison | Within_Study_Bias | Reporting_Bias | Indirectness | Imprecision | Heterogeneity | Incoherence | Overall_Confidence | Reason_for_downgrade |
| --- | --- | --- | --- | --- | --- | --- | --- | --- |
| Placebo:Resmetirom | Some concerns | Some concerns | No concerns | Major concerns | Major concerns | No concerns | Very low | Within-study bias, Reporting bias, Imprecision, Heterogeneity |
| Efruxifermin:Placebo | Some concerns | Some concerns | No concerns | Some concerns | Major concerns | No concerns | Low | Within-study bias, Reporting bias, Imprecision, Heterogeneity |
| Efruxifermin:Resmetirom | Some concerns | Some concerns | No concerns | Major concerns | Major concerns | No concerns | Very low | Within-study bias, Reporting bias, Imprecision, Heterogeneity |

## Table S4.4: Certainty of evidence for percentage change in Triglyceride

| Comparison | Within_Study_Bias | Reporting_Bias | Indirectness | Imprecision | Heterogeneity | Incoherence | Overall_Confidence | Reason_for_downgrade |
| --- | --- | --- | --- | --- | --- | --- | --- | --- |
| Placebo:Resmetirom | No concerns | Some concerns | No concerns | Some concerns | Major concerns | No concerns | Low | Reporting bias, Imprecision, Heterogeneity |
| Efruxifermin:Placebo | Some concerns | Some concerns | Some concerns | Some concerns | Major concerns | No concerns | Low | Within-study bias, Reporting bias, Indirectness, Imprecision, Heterogeneity |
| Efruxifermin:Resmetirom | Some concerns | Some concerns | No concerns | Major concerns | Major concerns | No concerns | Very low | Within-study bias, Reporting bias, Imprecision, Heterogeneity |

## Table S4.5: Certainty of evidence for percentage change in LDL

| Comparison | Within_Study_Bias | Reporting_Bias | Indirectness | Imprecision | Heterogeneity | Incoherence | Overall_Confidence | Reason_for_downgrade |
| --- | --- | --- | --- | --- | --- | --- | --- | --- |
| Placebo:Resmetirom | No concerns | Some concerns | No concerns | Some concerns | No concerns | No concerns | Moderate | Reporting bias, Imprecision |
| Efruxifermin:Placebo | Some concerns | Some concerns | Major concerns | Major concerns | No concerns | No concerns | Very low | Within-study bias, Reporting bias, Indirectness, Imprecision |
| Efruxifermin:Resmetirom | No concerns | Some concerns | No concerns | Major concerns | No concerns | No concerns | Low | Reporting bias, Imprecision |

## Table S4.6: Certainty of evidence for percentage change in HDL

| Comparison | Within_Study_Bias | Reporting_Bias | Indirectness | Imprecision | Heterogeneity | Incoherence | Overall_Confidence | Reason_for_downgrade |
| --- | --- | --- | --- | --- | --- | --- | --- | --- |
| Placebo:Resmetirom | No concerns | Some concerns | No concerns | Major concerns | No concerns | No concerns | Low | Reporting bias, Imprecision |
| Efruxifermin:Placebo | Some concerns | Some concerns | Major concerns | Some concerns | No concerns | No concerns | Low | Within-study bias, Reporting bias, Indirectness, Imprecision |
| Efruxifermin:Resmetirom | No concerns | Some concerns | No concerns | Some concerns | No concerns | No concerns | Moderate | Reporting bias, Imprecision |

## Table S4.7: Certainty of evidence for Serious adverse events

| Comparison | Within_Study_Bias | Reporting_Bias | Indirectness | Imprecision | Heterogeneity | Incoherence | Overall_Confidence | Reason_for_downgrade |
| --- | --- | --- | --- | --- | --- | --- | --- | --- |
| Placebo:Resmetirom | No concerns | Some concerns | No concerns | Major concerns | Major concerns | No concerns | Very low | Reporting bias, Imprecision, Heterogeneity |
| Efruxifermin:Placebo | Some concerns | Some concerns | No concerns | Major concerns | Major concerns | No concerns | Very low | Within-study bias, Reporting bias, Imprecision, Heterogeneity |
| Efruxifermin:Resmetirom | Some concerns | Some concerns | No concerns | Major concerns | Major concerns | No concerns | Very low | Within-study bias, Reporting bias, Imprecision, Heterogeneity |

## Table S4.8: Certainty of evidence for treatment discontinuation

| Comparison | Within_Study_Bias | Reporting_Bias | Indirectness | Imprecision | Heterogeneity | Incoherence | Overall_Confidence | Reason_for_downgrade |
| --- | --- | --- | --- | --- | --- | --- | --- | --- |
| Placebo:Resmetirom | No concerns | Some concerns | No concerns | No concerns | No concerns | No concerns | Moderate | Reporting bias |
| Efruxifermin:Placebo | Some concerns | Some concerns | No concerns | Major concerns | No concerns | No concerns | Low | Within-study bias, Reporting bias, Imprecision |
| Efruxifermin:Resmetirom | Some concerns | Some concerns | No concerns | Major concerns | No concerns | No concerns | Low | Within-study bias, Reporting bias, Imprecision |

## Table S4.9: Certainty of evidence for Nausea

| Comparison | Within_Study_Bias | Reporting_Bias | Indirectness | Imprecision | Heterogeneity | Incoherence | Overall_Confidence | Reason_for_downgrade |
| --- | --- | --- | --- | --- | --- | --- | --- | --- |
| Placebo:Resmetirom | No concerns | Some concerns | No concerns | No concerns | No concerns | No concerns | Moderate | Reporting bias |
| Efruxifermin:Placebo | Some concerns | Some concerns | No concerns | No concerns | No concerns | No concerns | Moderate | Within-study bias, Reporting bias |
| Efruxifermin:Resmetirom | Some concerns | Some concerns | No concerns | Major concerns | No concerns | No concerns | Low | Within-study bias, Reporting bias, Imprecision |

## Table S4.10: Certainty of evidence for diarrhea

| Comparison | Within_Study_Bias | Reporting_Bias | Indirectness | Imprecision | Heterogeneity | Incoherence | Overall_Confidence | Reason_for_downgrade |
| --- | --- | --- | --- | --- | --- | --- | --- | --- |
| Placebo:Resmetirom | No concerns | Some concerns | No concerns | No concerns | No concerns | No concerns | Moderate | Reporting bias |
| Efruxifermin:Placebo | Some concerns | Some concerns | No concerns | No concerns | No concerns | No concerns | Moderate | Within-study bias, Reporting bias |
| Efruxifermin:Resmetirom | Some concerns | Some concerns | No concerns | Major concerns | No concerns | No concerns | Low | Within-study bias, Reporting bias, Imprecision |

## Table S4.11: Certainty of evidence for Abdominal pain

| Comparison | Within_Study_Bias | Reporting_Bias | Indirectness | Imprecision | Heterogeneity | Incoherence | Overall_Confidence | Reason_for_downgrade |
| --- | --- | --- | --- | --- | --- | --- | --- | --- |
| Efruxifermin:Placebo | Some concerns | Some concerns | No concerns | Major concerns | No concerns | No concerns | Low | Within-study bias, Reporting bias, Imprecision |
| Placebo:Resmetirom | No concerns | Some concerns | No concerns | No concerns | No concerns | No concerns | Moderate | Reporting bias |
| Efruxifermin:Resmetirom | Some concerns | Some concerns | No concerns | Major concerns | No concerns | No concerns | Low | Within-study bias, Reporting bias, Imprecision |

# Supplement S5. Publication bias (funnel plot)

##

## Figure S5.1 Percentage Change in MRI-PDFF


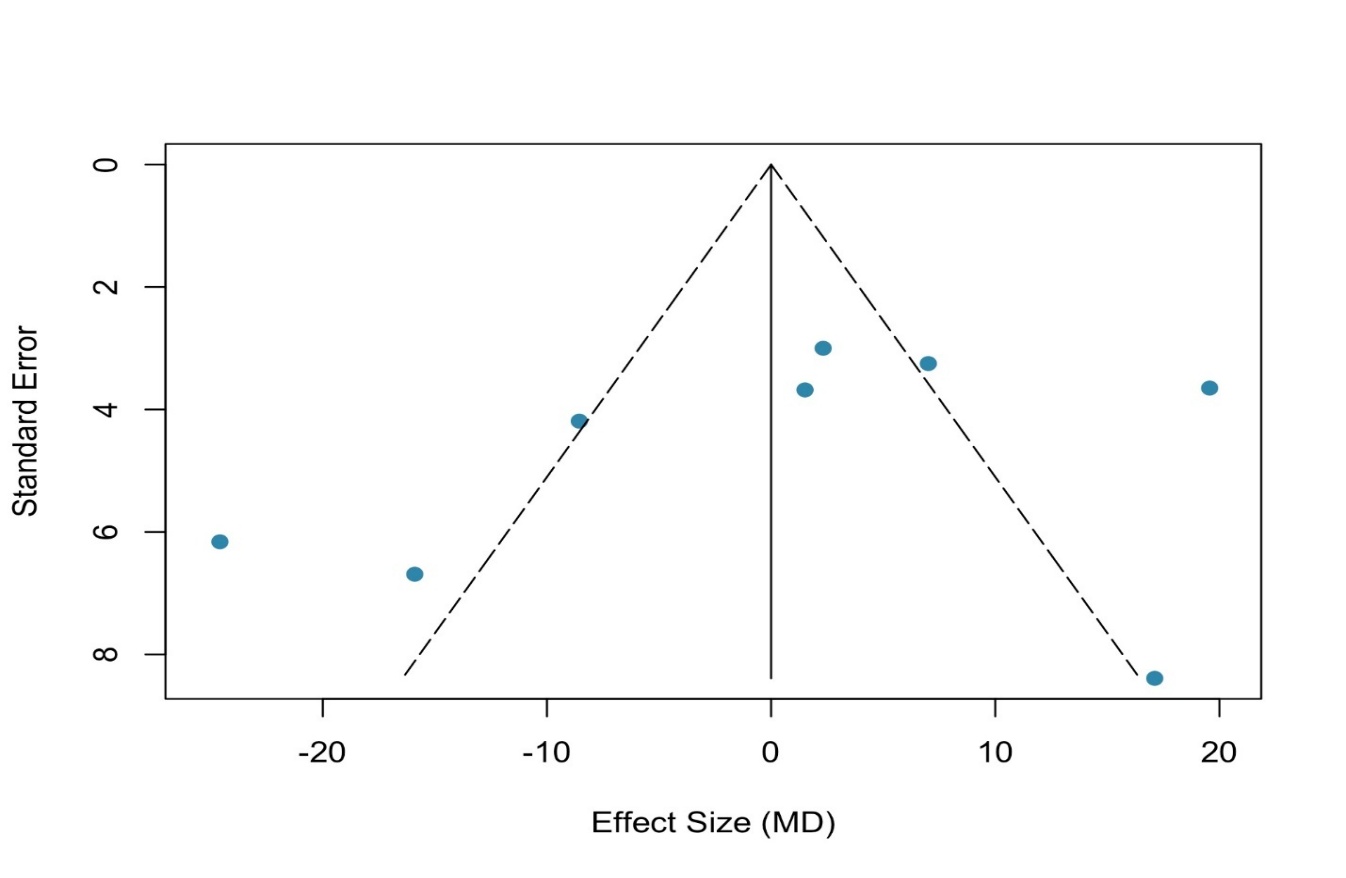


## Figure S5.2 Change in ALT


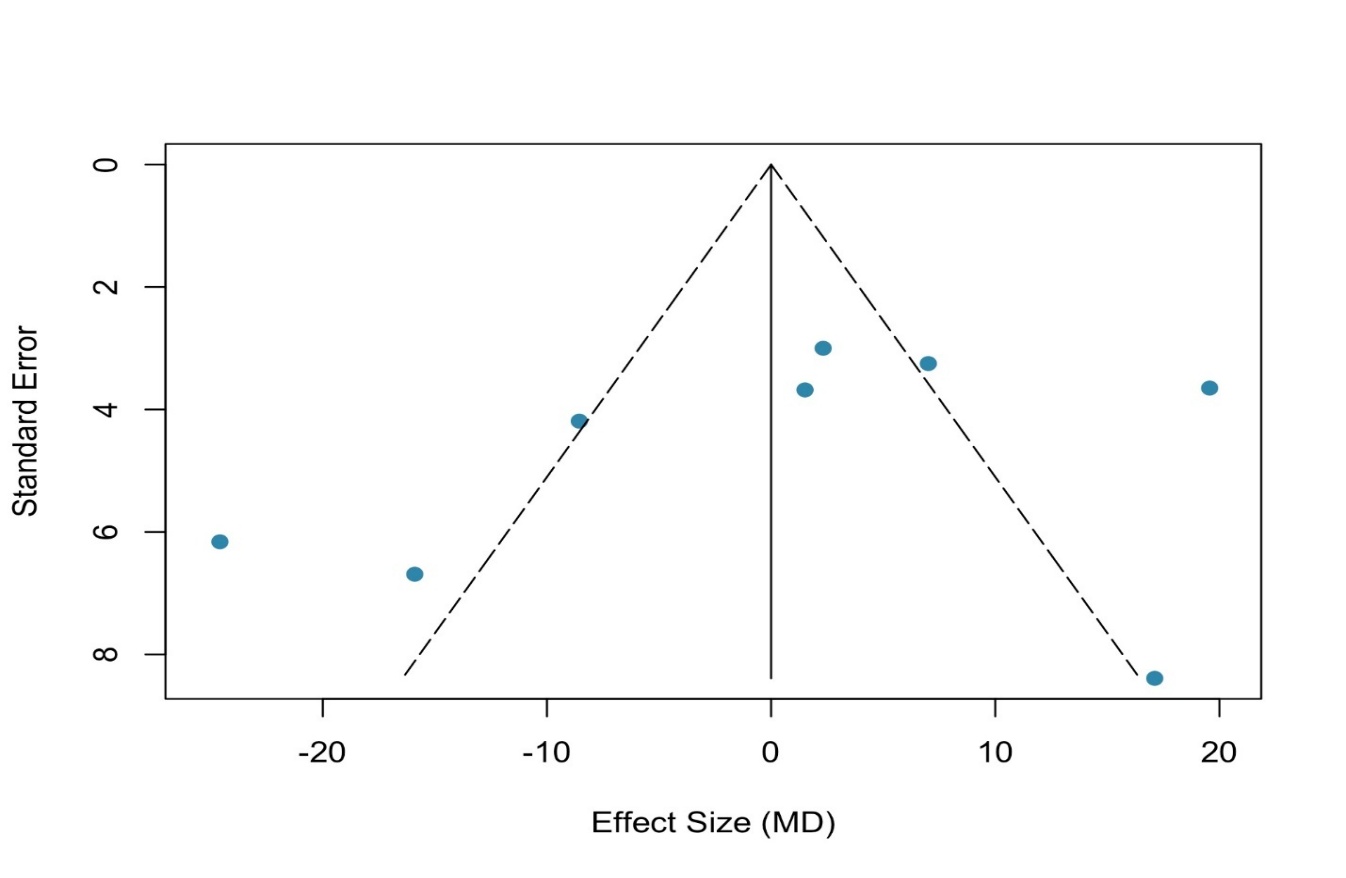


## Figure S5.3 Change in AST


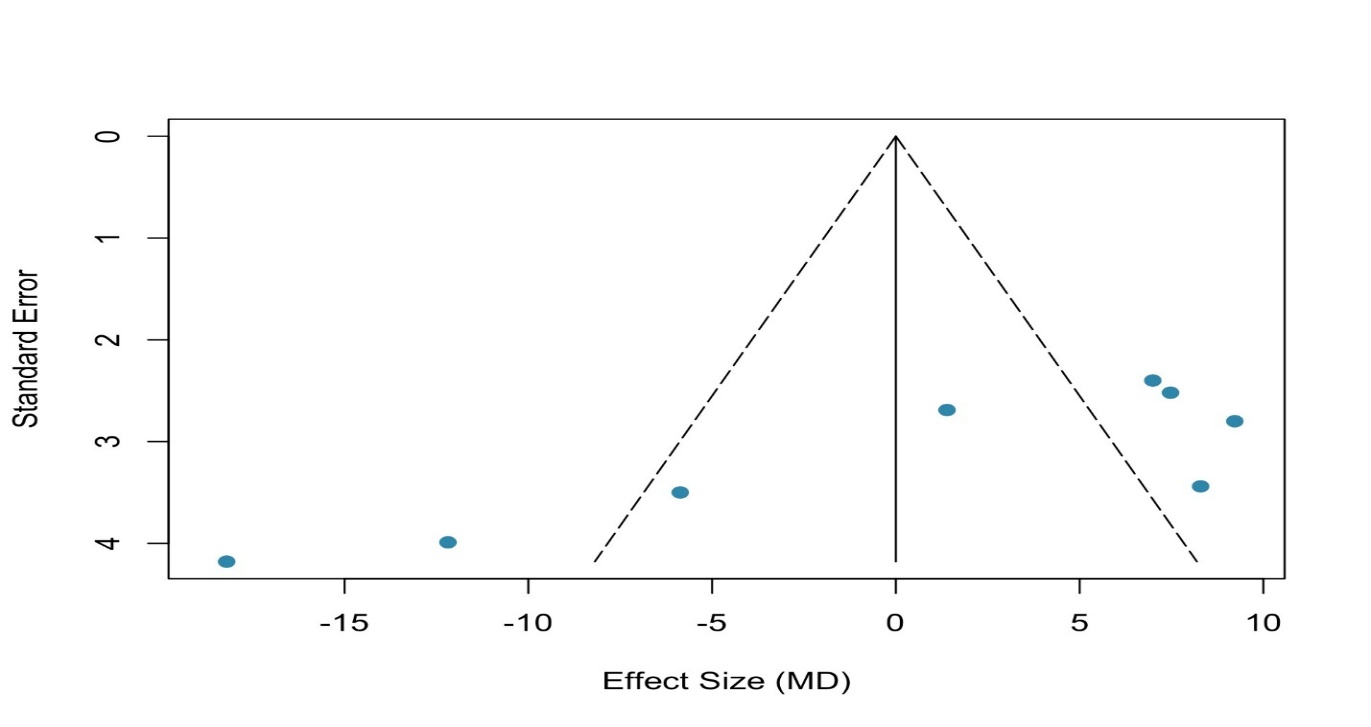


## Figure S5.4 Percentage Change in Triglyceride


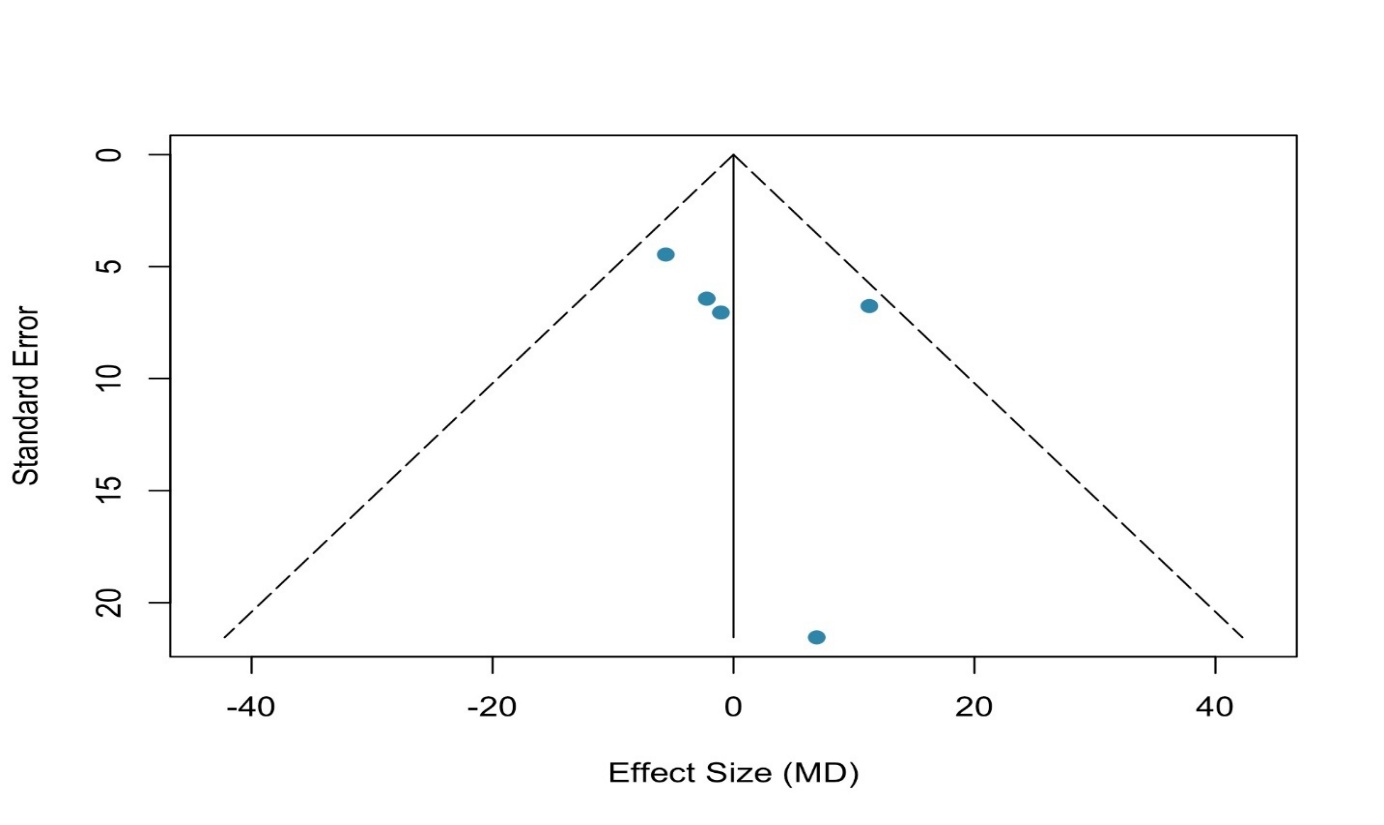


## Figure S5.5 Percentage Change in LDL


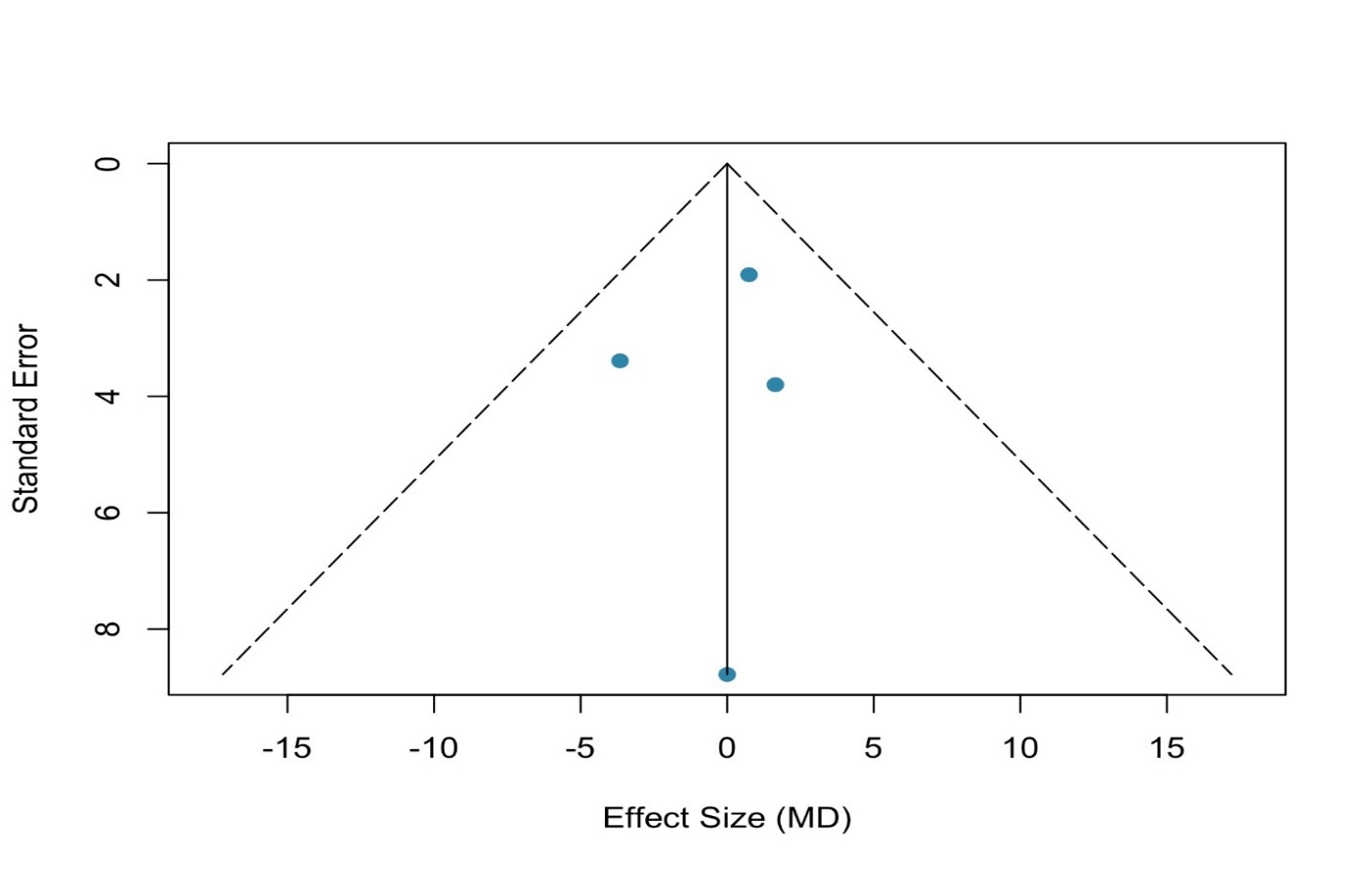


## Figure S5.6 Percentage Change in HDL


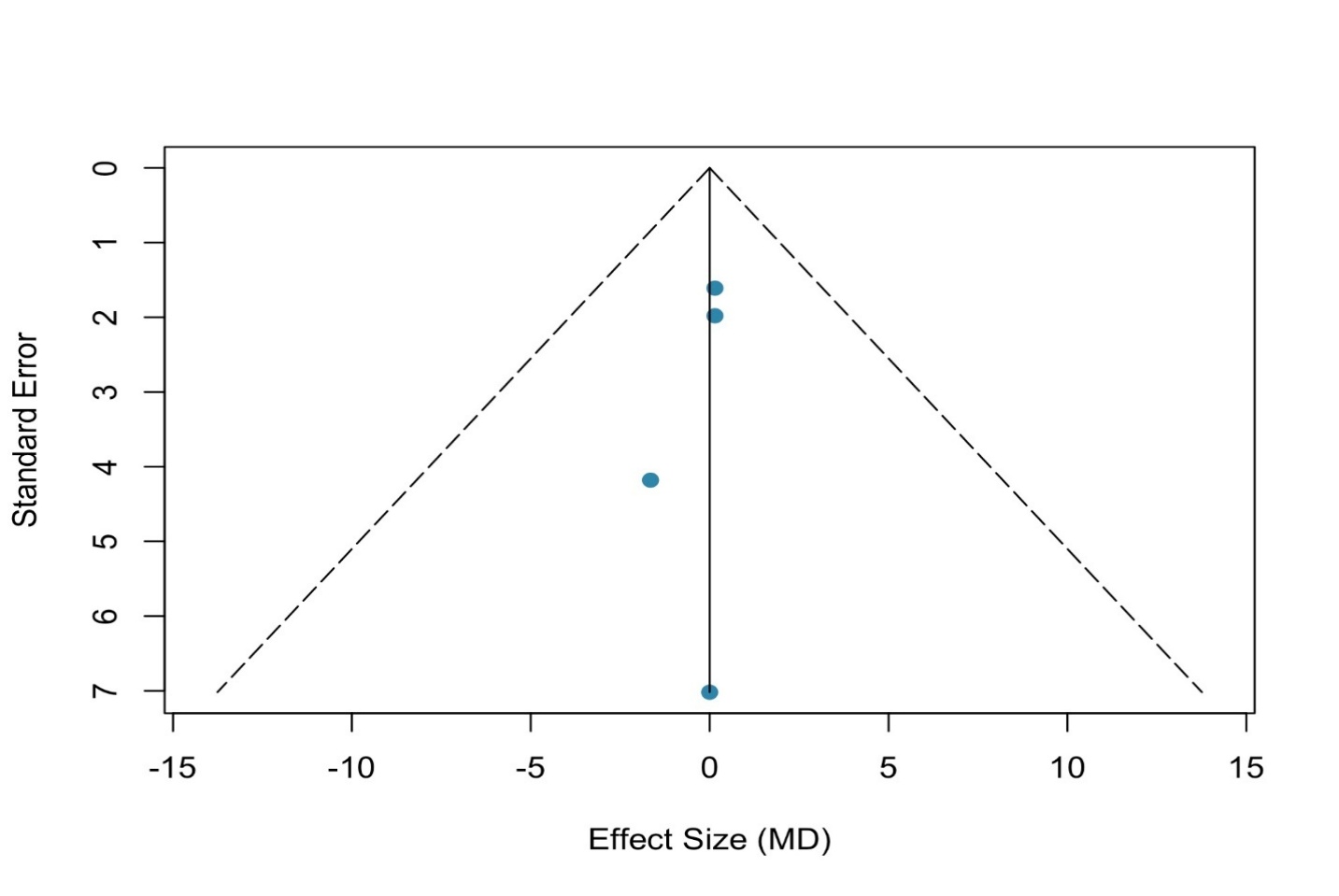


## Figure S5.7 Serious adverse events


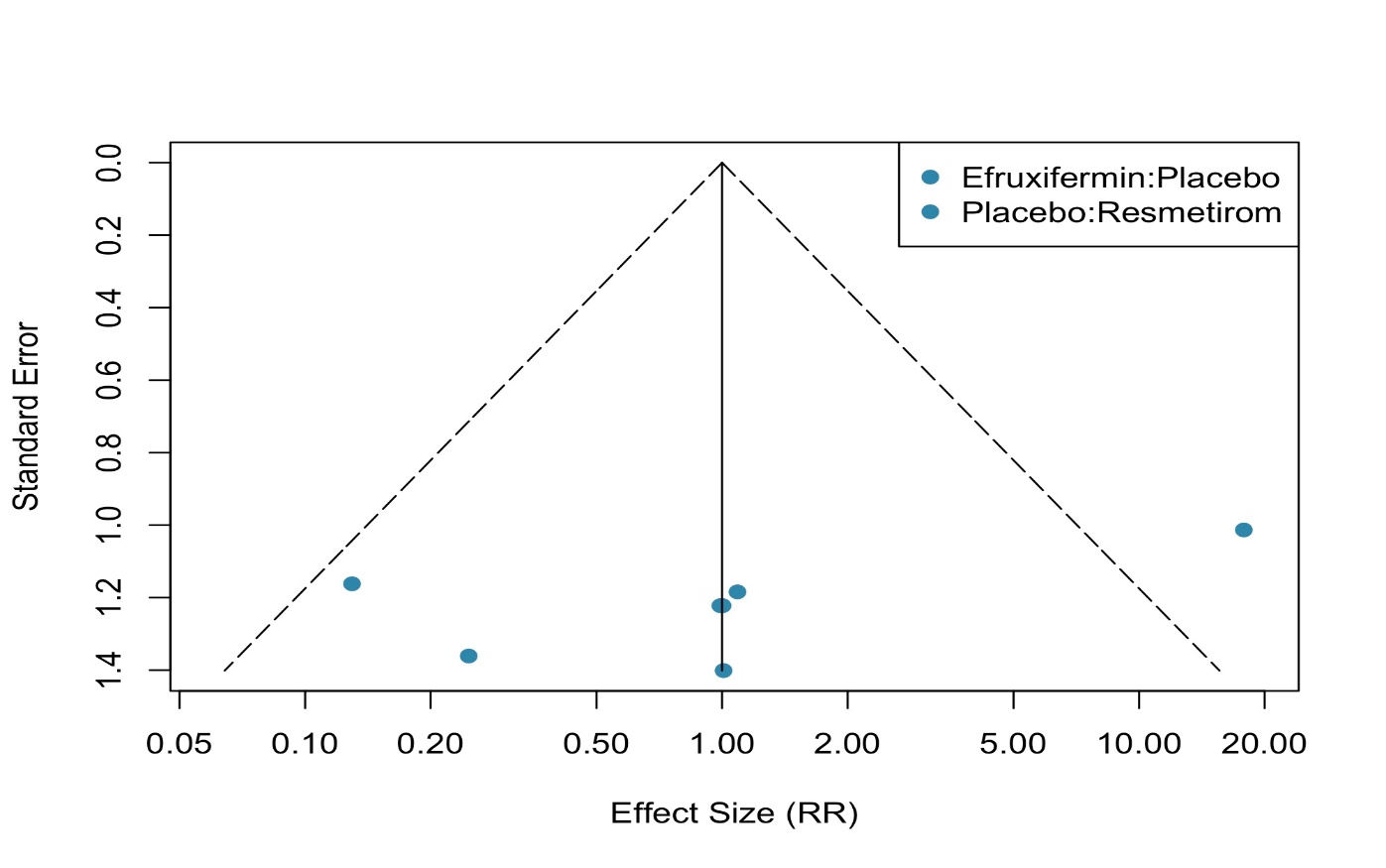


## Figure S5.8 Treatment Discontinuation


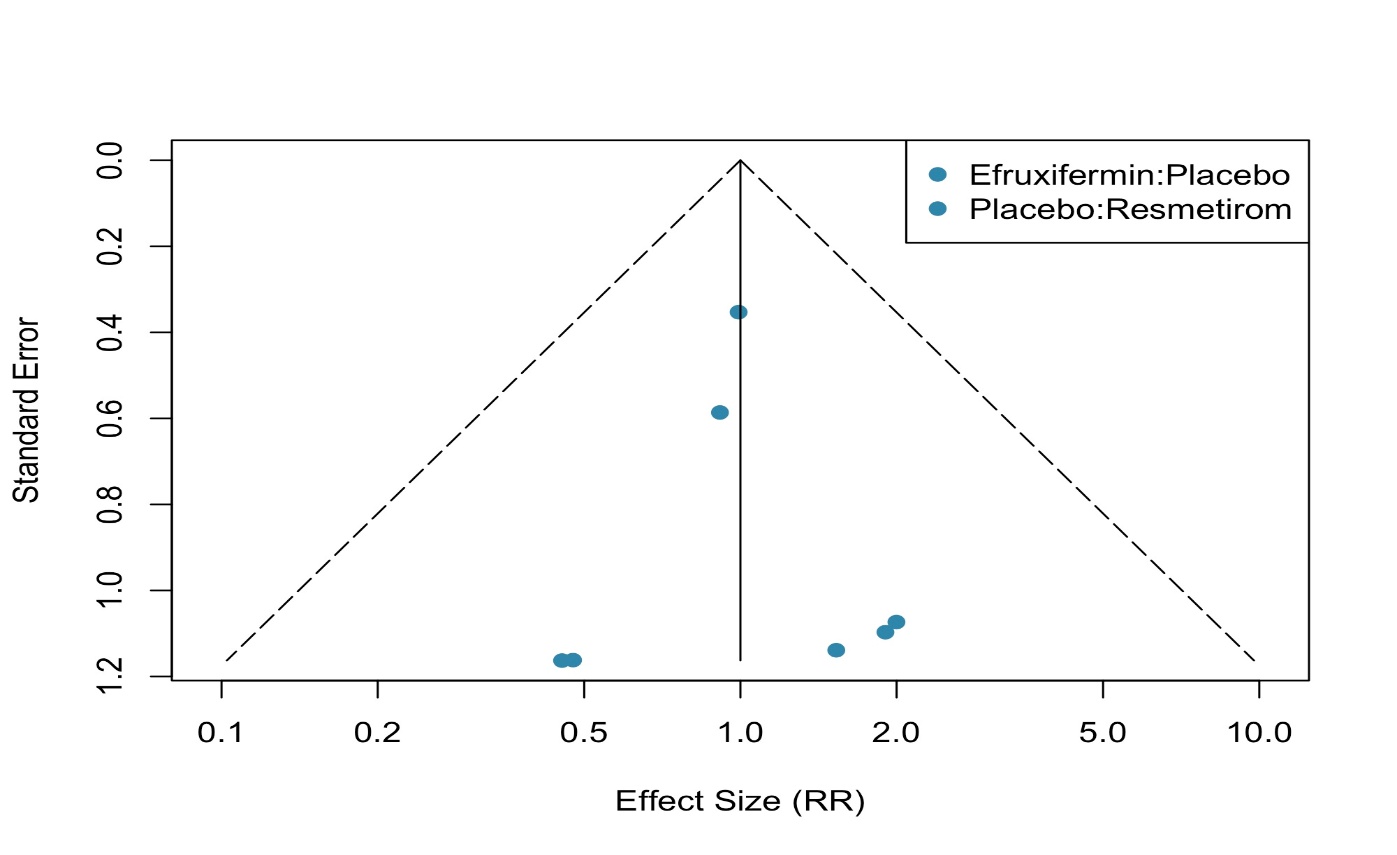


## Figure S5.9 Nausea


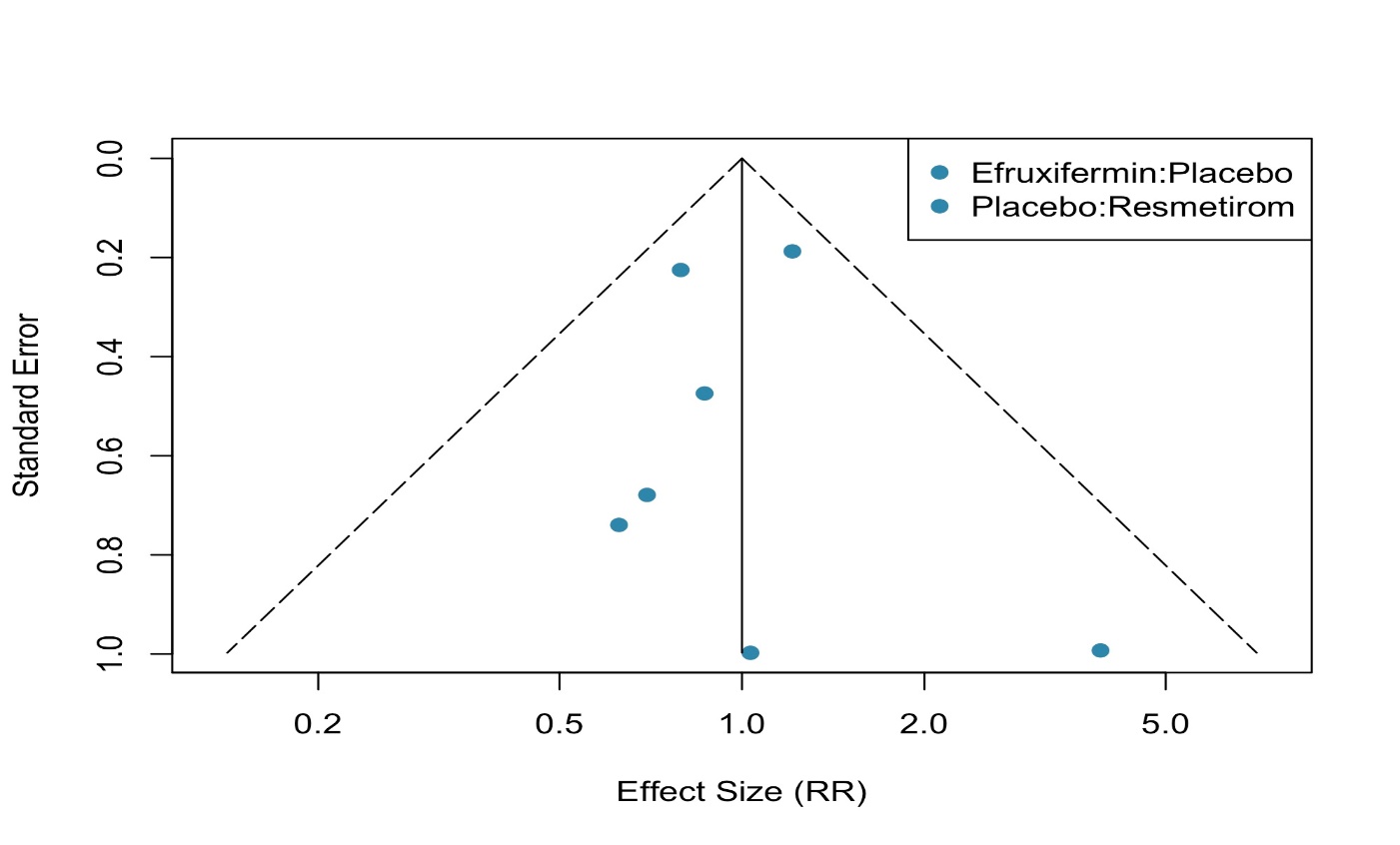


## Figure S5.10 Diarrhea


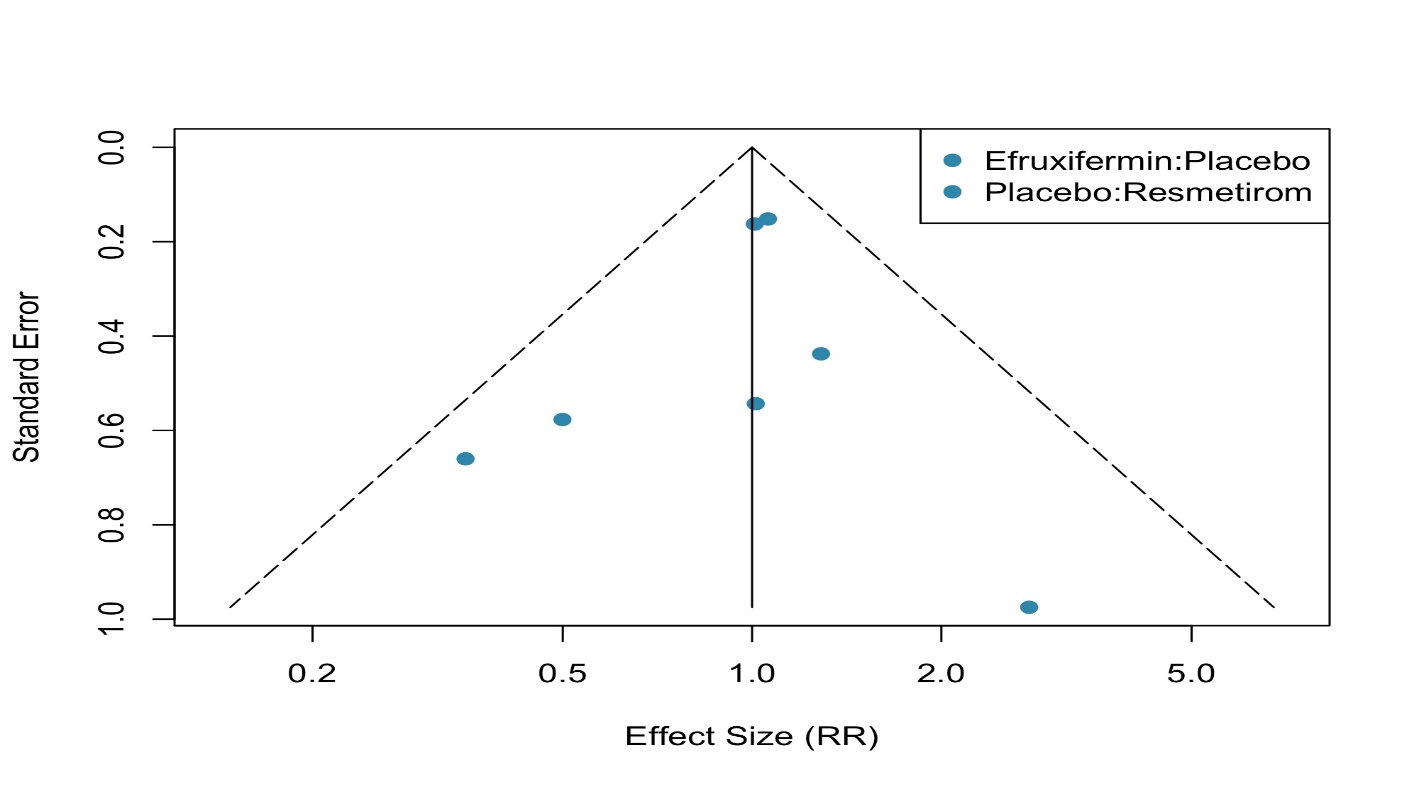


## Figure S5.11 Abdominal pain


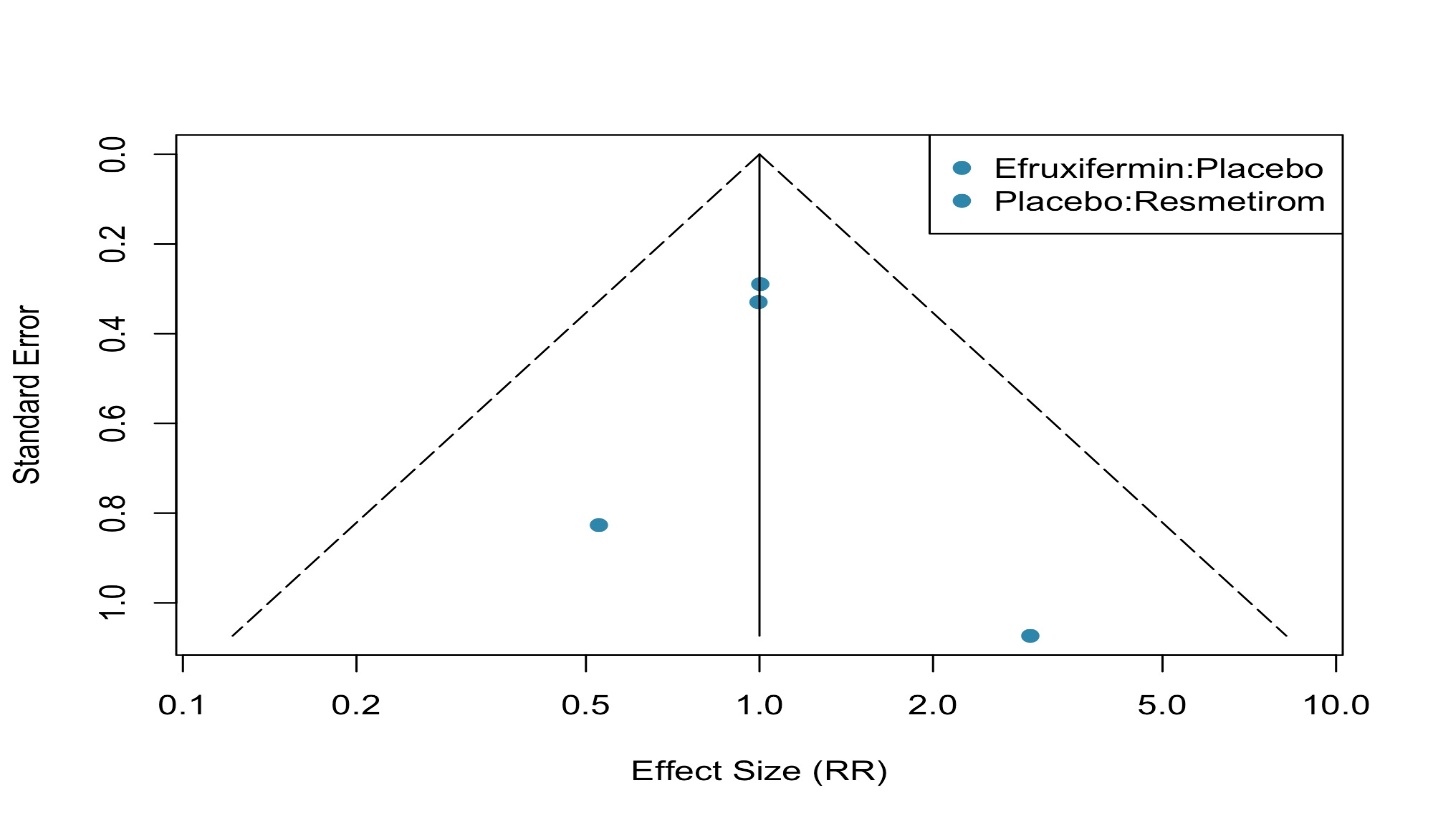


# Supplement 6: Network meta-analysis results (league)

## Table 6.1. Network meta-analysis results for Percentage change in MRI-PDFF

|  | Efruxifermin | Placebo | Resmetirom |
| --- | --- | --- | --- |
| Efruxifermin | Efruxifermin | -62.83 [-72.30; -53.36] | . |
| Placebo | -62.83 [-72.30; -53.36] | Placebo | 37.15 [ 29.88; 44.43] |
| Resmetirom | -25.68 [-37.62; -13.74] | 37.15 [ 29.88; 44.43] | Resmetirom |

## Table 6.2. Network meta-analysis results for change in ALT

|  | Efruxifermin | Placebo | Resmetirom |
| --- | --- | --- | --- |
| Efruxifermin | Efruxifermin | -10.57 [-24.10; 2.96] | . |
| Placebo | -10.57 [-24.10; 2.96] | Placebo | 9.29 [ -4.60; 23.18] |
| Resmetirom | -1.28 [-20.68; 18.11] | 9.29 [ -4.60; 23.18] | Resmetirom |

## Table 6.3. Network meta-analysis results for change in AST

|  | Efruxifermin | Placebo | Resmetirom |
| --- | --- | --- | --- |
| Efruxifermin | Efruxifermin | -14.32 [-23.92; -4.72] | . |
| Placebo | -14.32 [-23.92; -4.72] | Placebo | 2.81 [ -6.79; 12.40] |
| Resmetirom | -11.52 [-25.09; 2.06] | 2.81 [ -6.79; 12.40] | Resmetirom |

## Table 6.4. Network meta-analysis results for Percentage change in Triglyceride

|  | Efruxifermin | Placebo | Resmetirom |
| --- | --- | --- | --- |
| Efruxifermin | Efruxifermin | -36.95 [-52.67; -21.24] | . |
| Placebo | -36.95 [-52.67; -21.24] | Placebo | 24.72 [ 16.14; 33.31] |
| Resmetirom | -12.23 [-30.14; 5.68] | 24.72 [ 16.14; 33.31] | Resmetirom |

## Table 6.5. Network meta-analysis results for Percentage Change in LDL

|  | Efruxifermin | Placebo | Resmetirom |
| --- | --- | --- | --- |
| Efruxifermin | Efruxifermin | -1.87 [-19.08; 15.34] | . |
| Placebo | -1.87 [-19.08; 15.34] | Placebo | 15.66 [ 12.67; 18.64] |
| Resmetirom | 13.79 [ -3.68; 31.25] | 15.66 [ 12.67; 18.64] | Resmetirom |

## Table 6.6 Network meta-analysis results for Percentage change in HDL

|  | Efruxifermin | Placebo | Resmetirom |
| --- | --- | --- | --- |
| Efruxifermin | Efruxifermin | 35.31 [ 21.55; 49.07] | . |
| Placebo | 35.31 [ 21.55; 49.07] | Placebo | -2.15 [ -4.49; 0.20] |
| Resmetirom | 33.16 [ 19.20; 47.12] | -2.15 [ -4.49; 0.20] | Resmetirom |

## Table 6.7. Network meta-analysis results for serious adverse events

|  | Efruxifermin | Placebo | Resmetirom |
| --- | --- | --- | --- |
| Efruxifermin | Efruxifermin | 1.929 [0.311; 11.964] | . |
| Placebo | 1.929 [0.311; 11.964] | Placebo | 2.032 [0.229; 18.031] |
| Resmetirom | 3.921 [0.228; 67.454] | 2.032 [0.229; 18.031] | Resmetirom |

## Table 6.8. Network meta-analysis results for Treatment Discontinuation

|  | Efruxifermin | Placebo | Resmetirom |
| --- | --- | --- | --- |
| Efruxifermin | Efruxifermin | 2.102 [0.700; 6.309] | . |
| Placebo | 2.102 [0.700; 6.309] | Placebo | 0.446 [0.252; 0.792] |
| Resmetirom | 0.938 [0.272; 3.240] | 0.446 [0.252; 0.792] | Resmetirom |

## Table 6.9. Network meta-analysis results for Nausea

|  | Efruxifermin | Placebo | Resmetirom |
| --- | --- | --- | --- |
| Efruxifermin | Efruxifermin | 3.228 [1.656; 6.289] | . |
| Placebo | 3.228 [1.656; 6.289] | Placebo | 0.545 [0.413; 0.719] |
| Resmetirom | 1.758 [0.854; 3.621] | 0.545 [0.413; 0.719] | Resmetirom |

## Table 6.10. Network meta-analysis results for Diarrhea

|  | Efruxifermin | Placebo | Resmetirom |
| --- | --- | --- | --- |
| Efruxifermin | Efruxifermin | 1.813 [1.017; 3.229] | . |
| Placebo | 1.813 [1.017; 3.229] | Placebo | 0.440 [0.342; 0.565] |
| Resmetirom | 0.797 [0.424; 1.496] | 0.440 [0.342; 0.565] | Resmetirom |

## Table 6.11. Network meta-analysis results for Abdominal pain

|  | Efruxifermin | Placebo | Resmetirom |
| --- | --- | --- | --- |
| Efruxifermin | Efruxifermin | 1.424 [0.395; 5.141] | . |
| Placebo | 1.424 [0.395; 5.141] | Placebo | 0.623 [0.407; 0.954] |
| Resmetirom | 0.887 [0.229; 3.430] | 0.623 [0.407; 0.954] | Resmetirom |

# Supplement 7: Network meta-analysis results for each outcome

## Figure S7.1: Network meta-analysis results for Percent change in MRI-PDFF


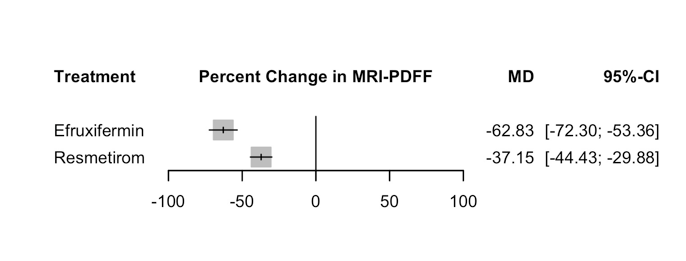


## Figure S7.2: Network meta-analysis results for change in ALT


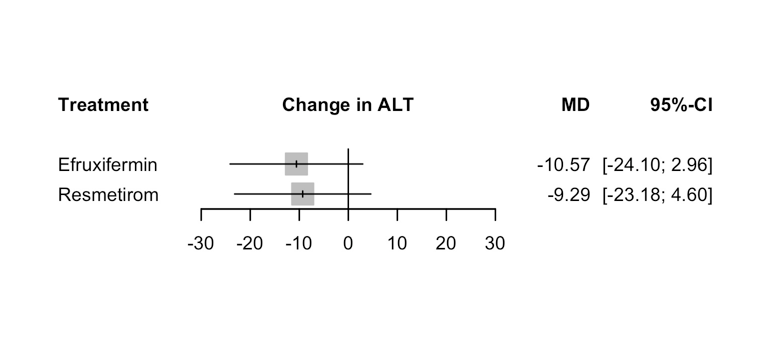


## Figure S7.3: Network meta-analysis results for change in AST


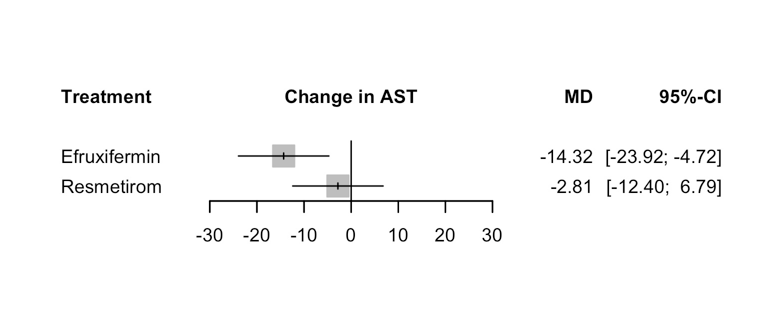


## Figure S7.4: Network meta-analysis results for percentage change in triglyceride


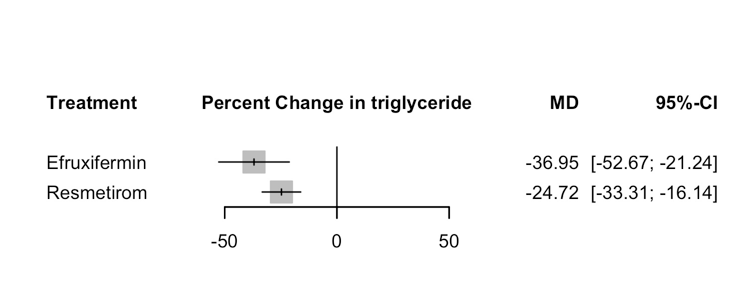


## Figure S7.5: Network meta-analysis results for percentage change in LDL


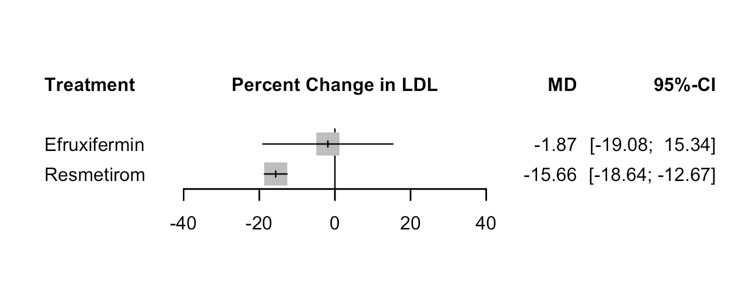


## Figure S7.6: Network meta-analysis results for percentage change in HDL


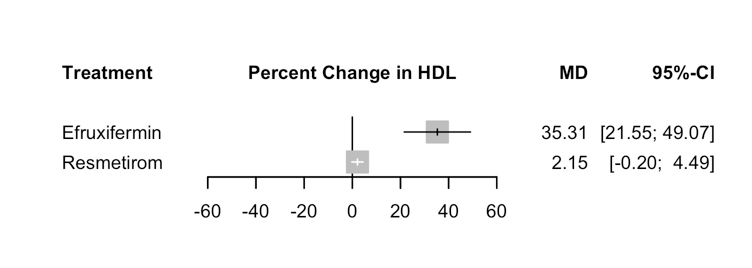


## Figure S7.7: Network meta-analysis results for serious adverse events


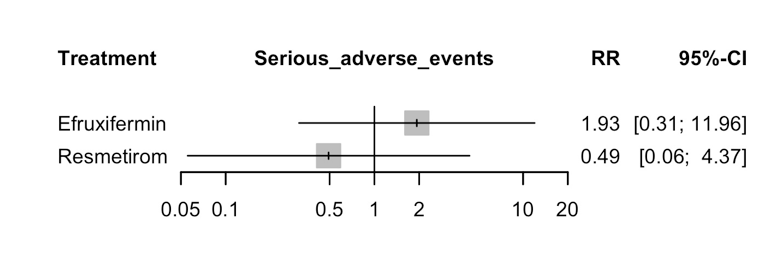


## Figure S7.8: Network meta-analysis results for Treatment Discontinuation


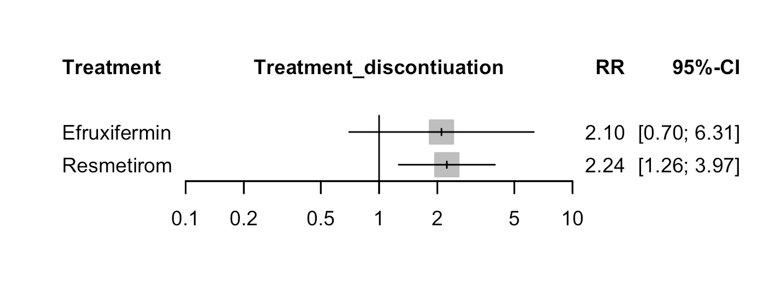


## Figure S7.9: Network meta-analysis results for Nausea


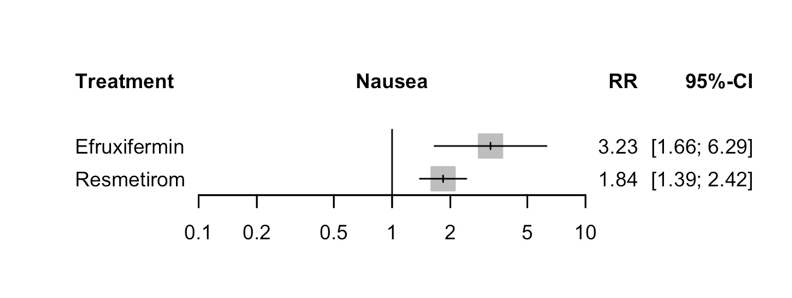


## Figure S7.10: Network meta-analysis results for Diarrhea


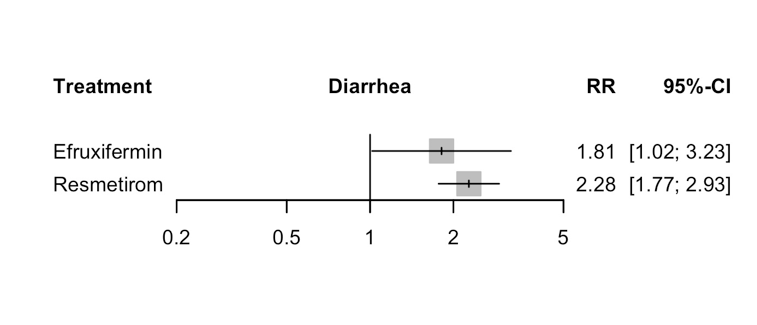


## Figure S7.11: Network meta-analysis results for Abdominal pain


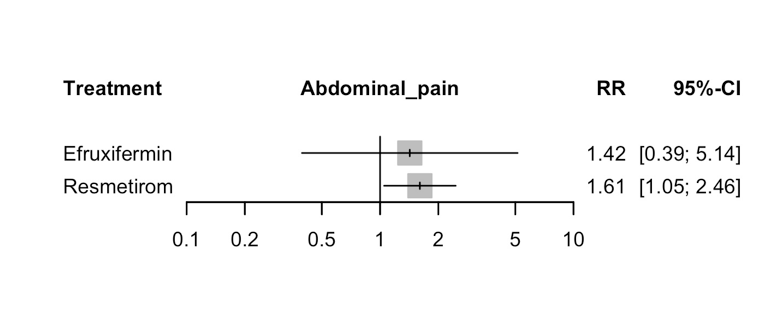


# Supplement S8. Network plots of treatment comparisons

## Figure S8.1 Network Plot of Treatment Comparisons for Percent change in MRI-PDFF


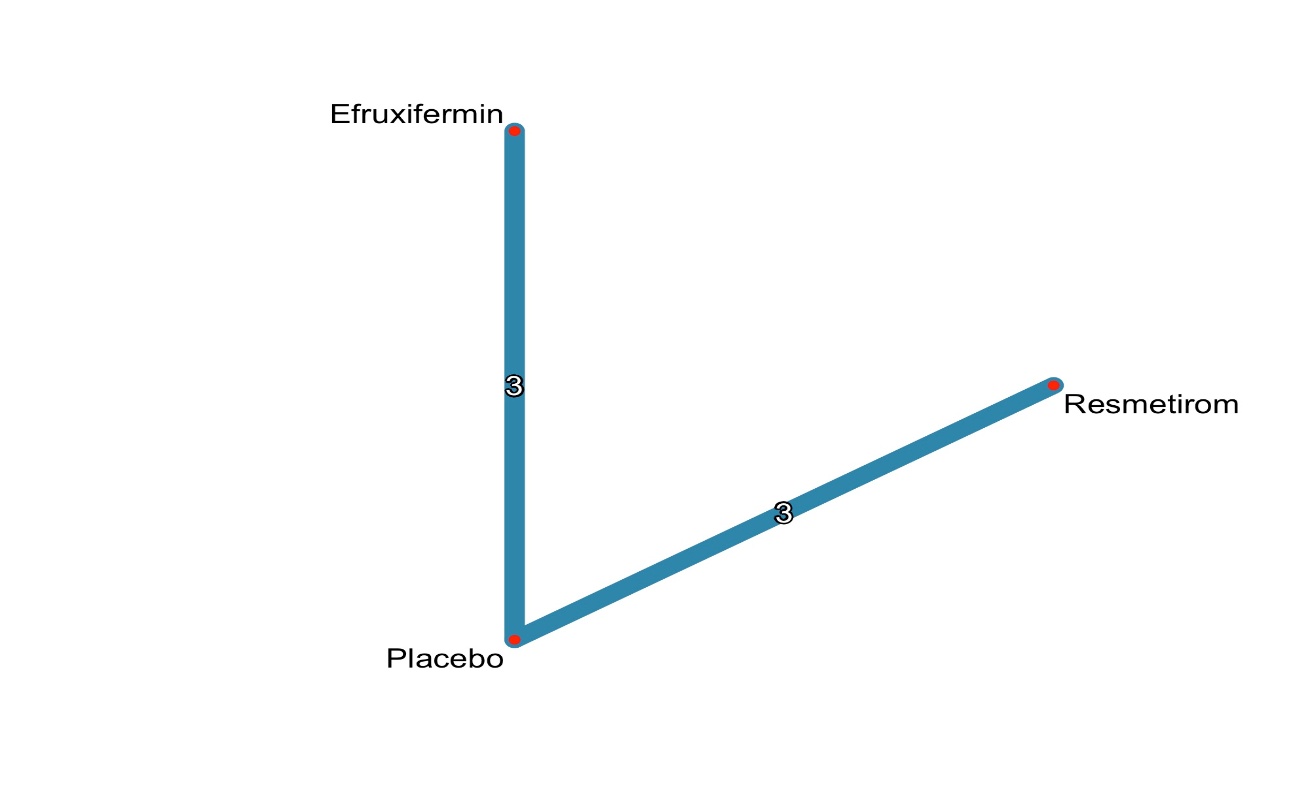


## Figure S8.2 Network Plot of Treatment Comparisons for change in ALT


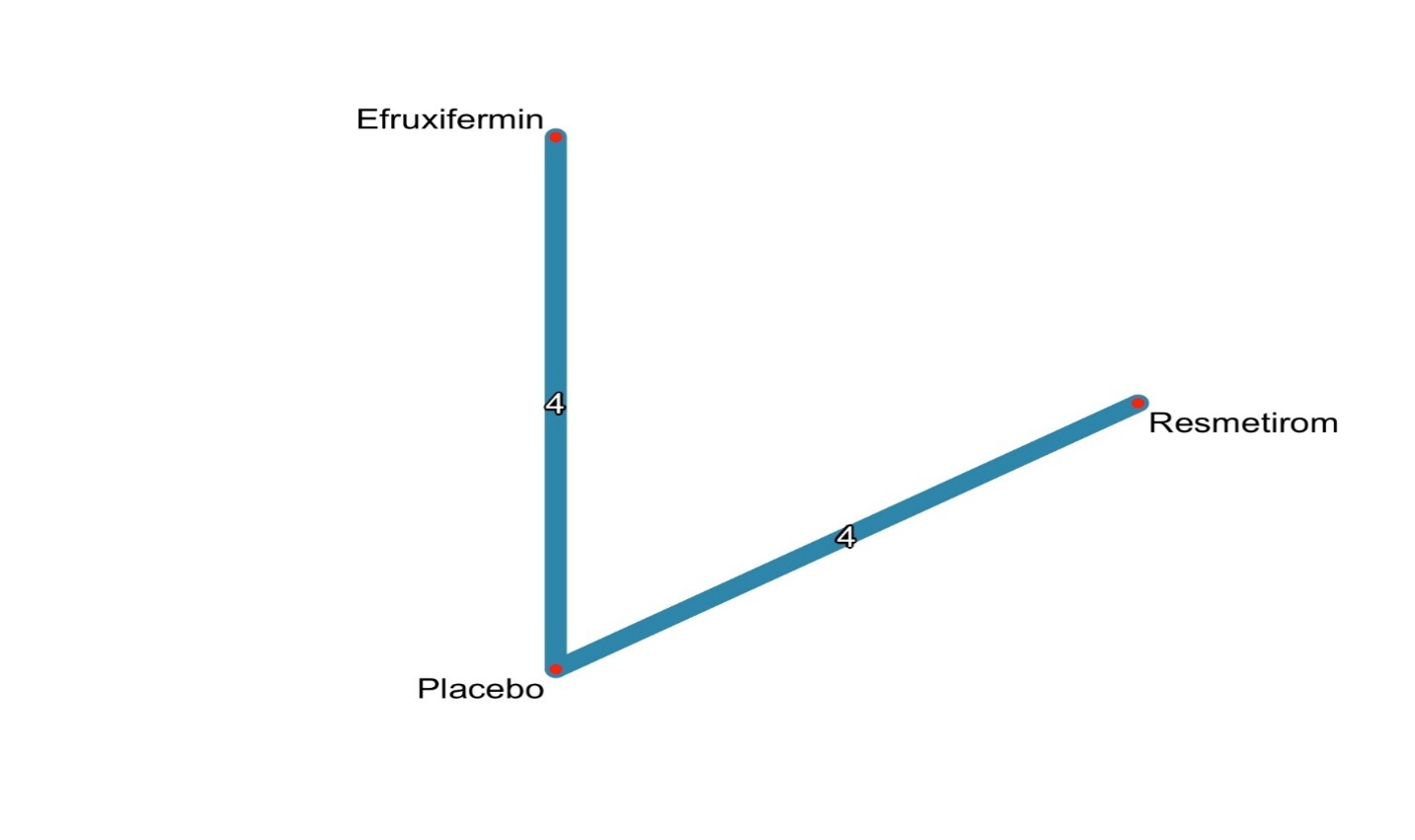


## Figure S8.3 Network Plot of Treatment Comparisons for change in AST


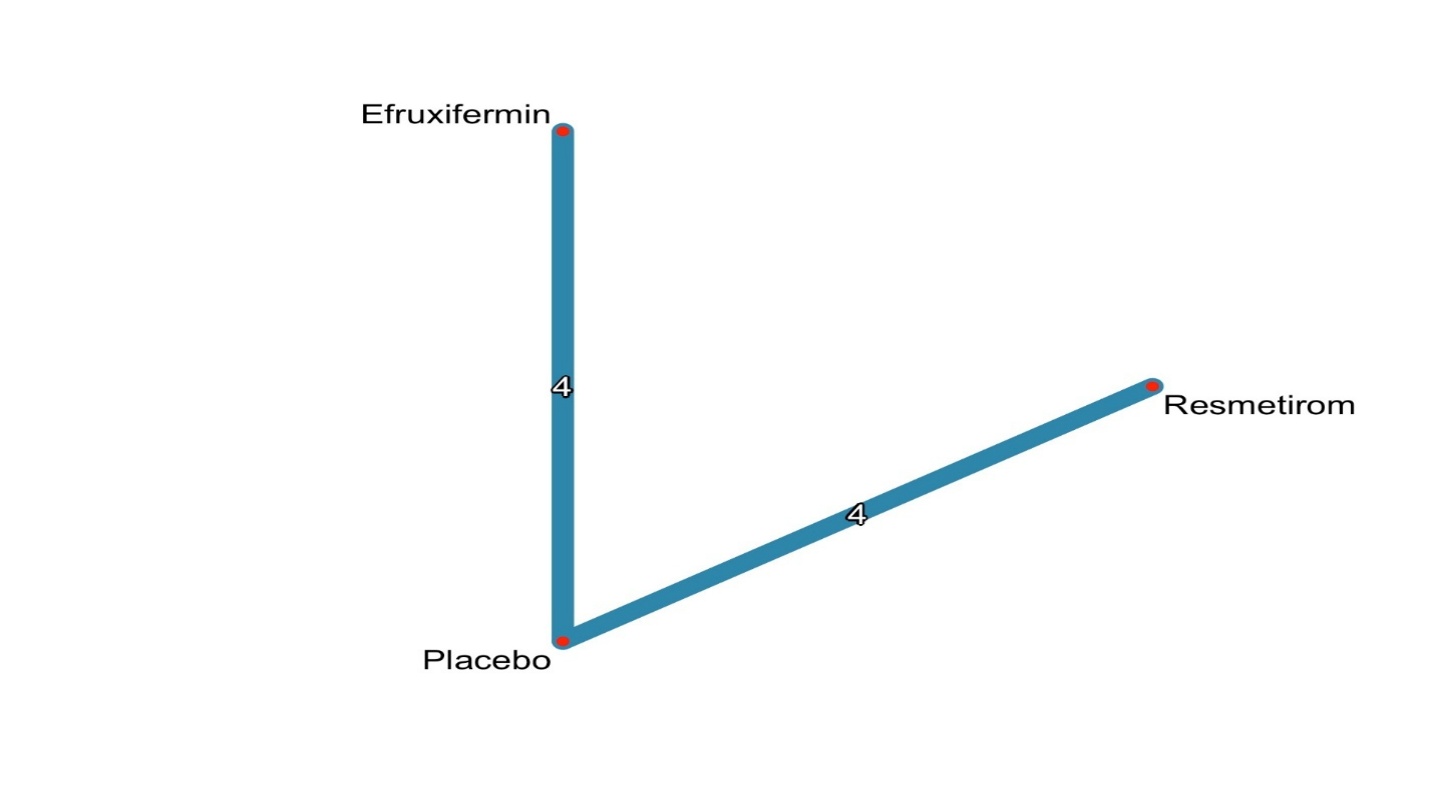


## Figure S8.4 Network Plot of Treatment Comparisons for Percent change in Triglyceride


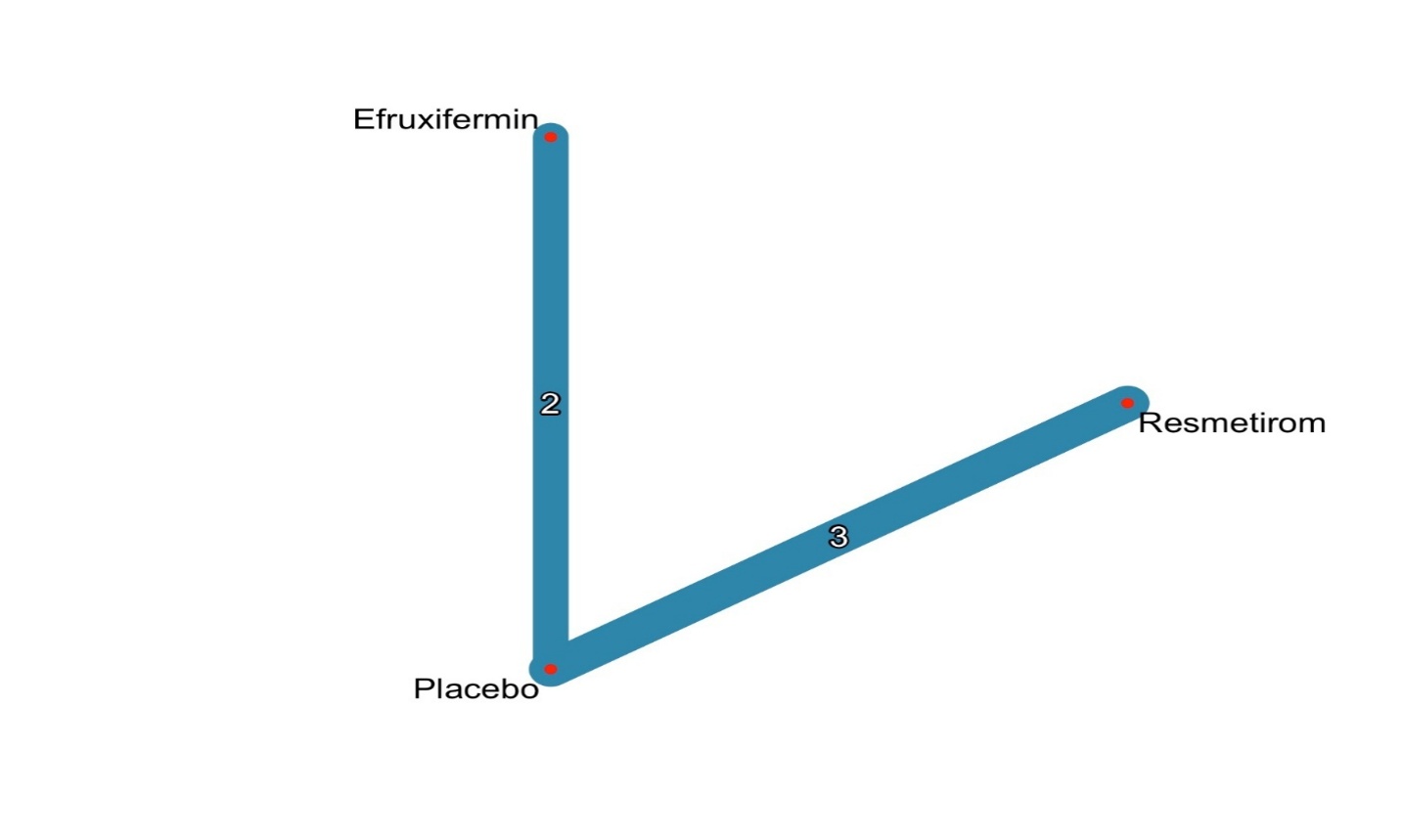


Figure S8.5 Network Plot of Treatment Comparisons for Percent change in LDL


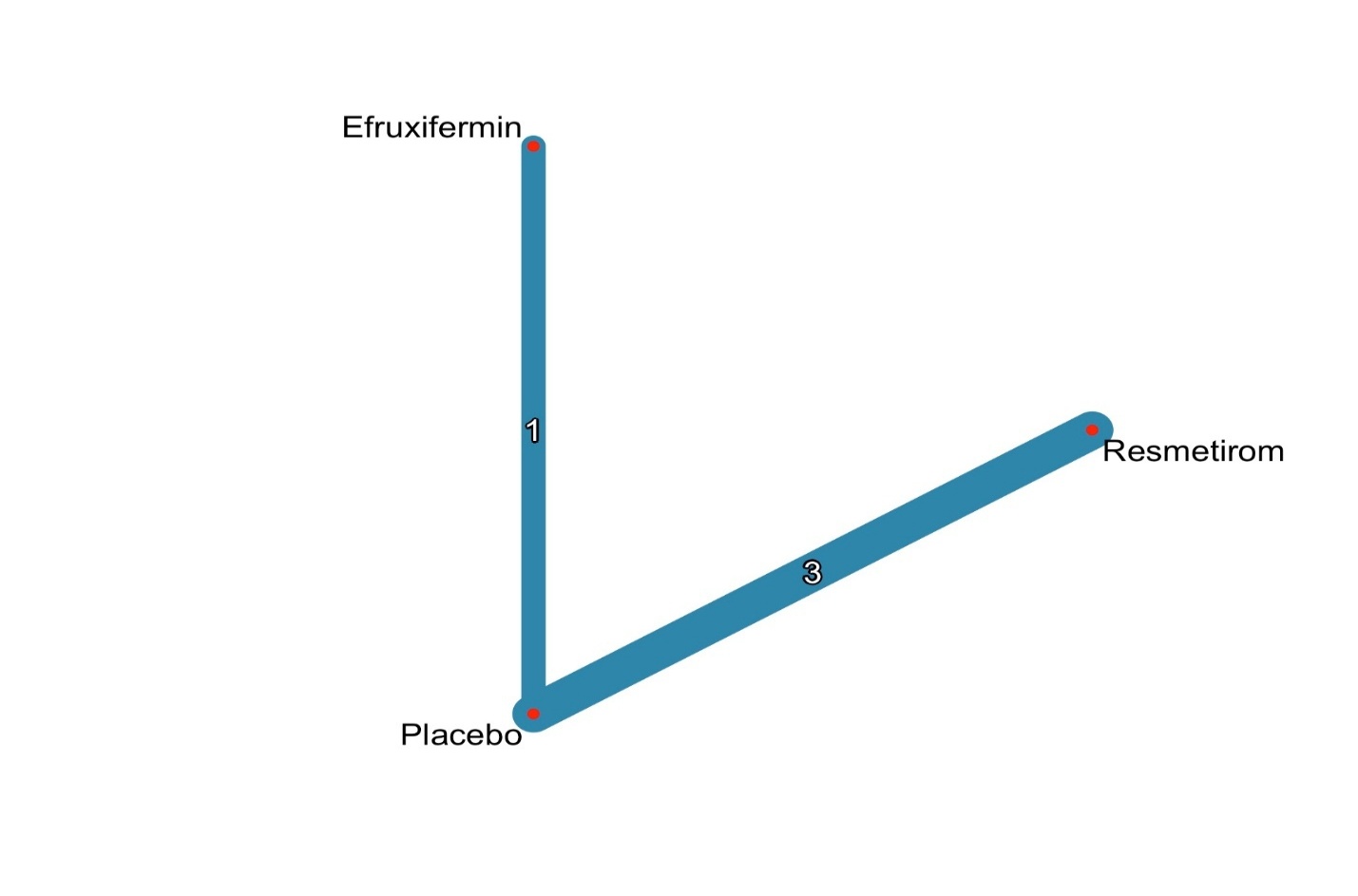


## Figure S8.6 Network Plot of Treatment Comparisons for Percent change in HDL


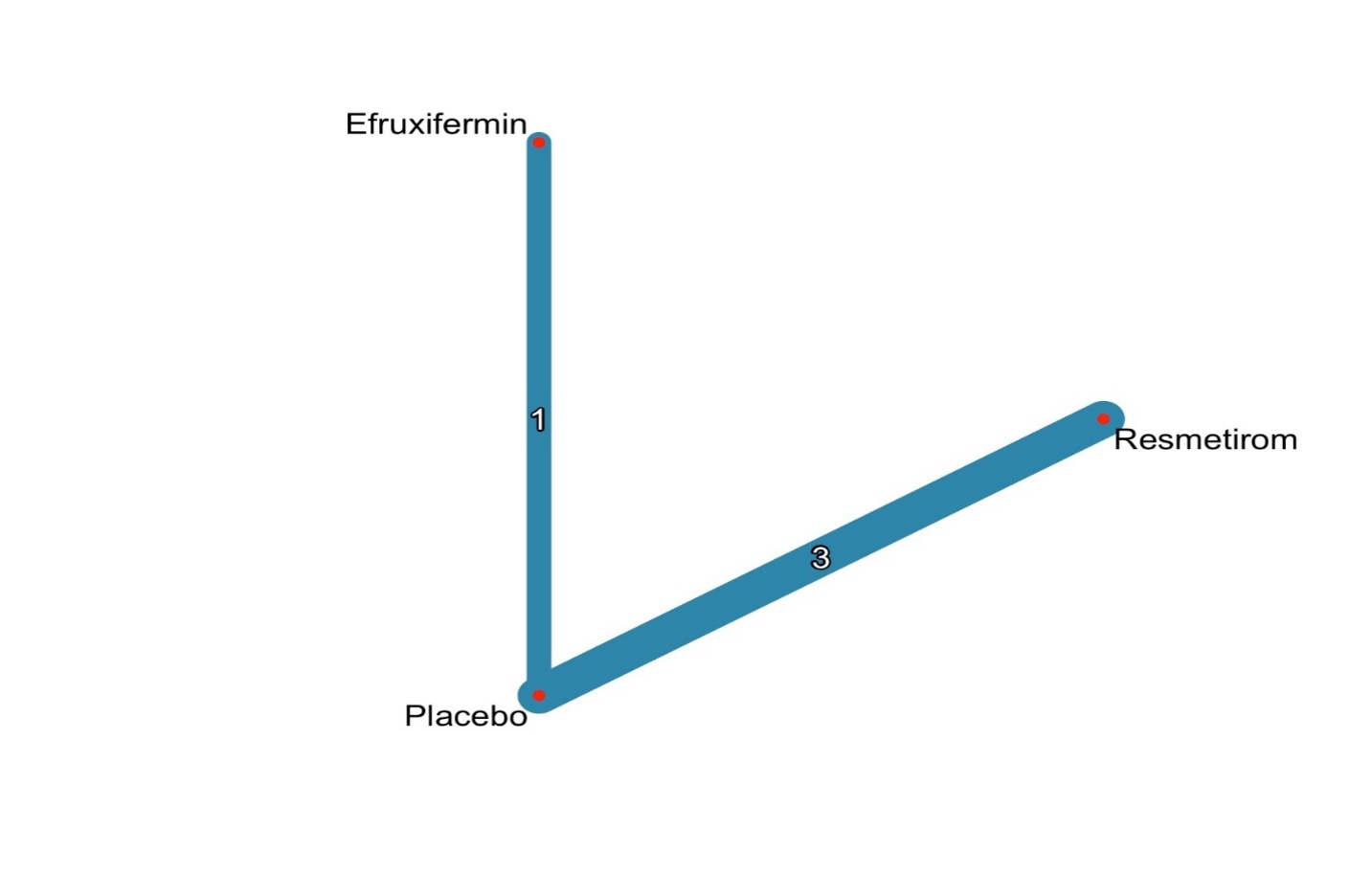


## Figure S8.7 Network Plot of Treatment Comparisons for Serious Adverse Events


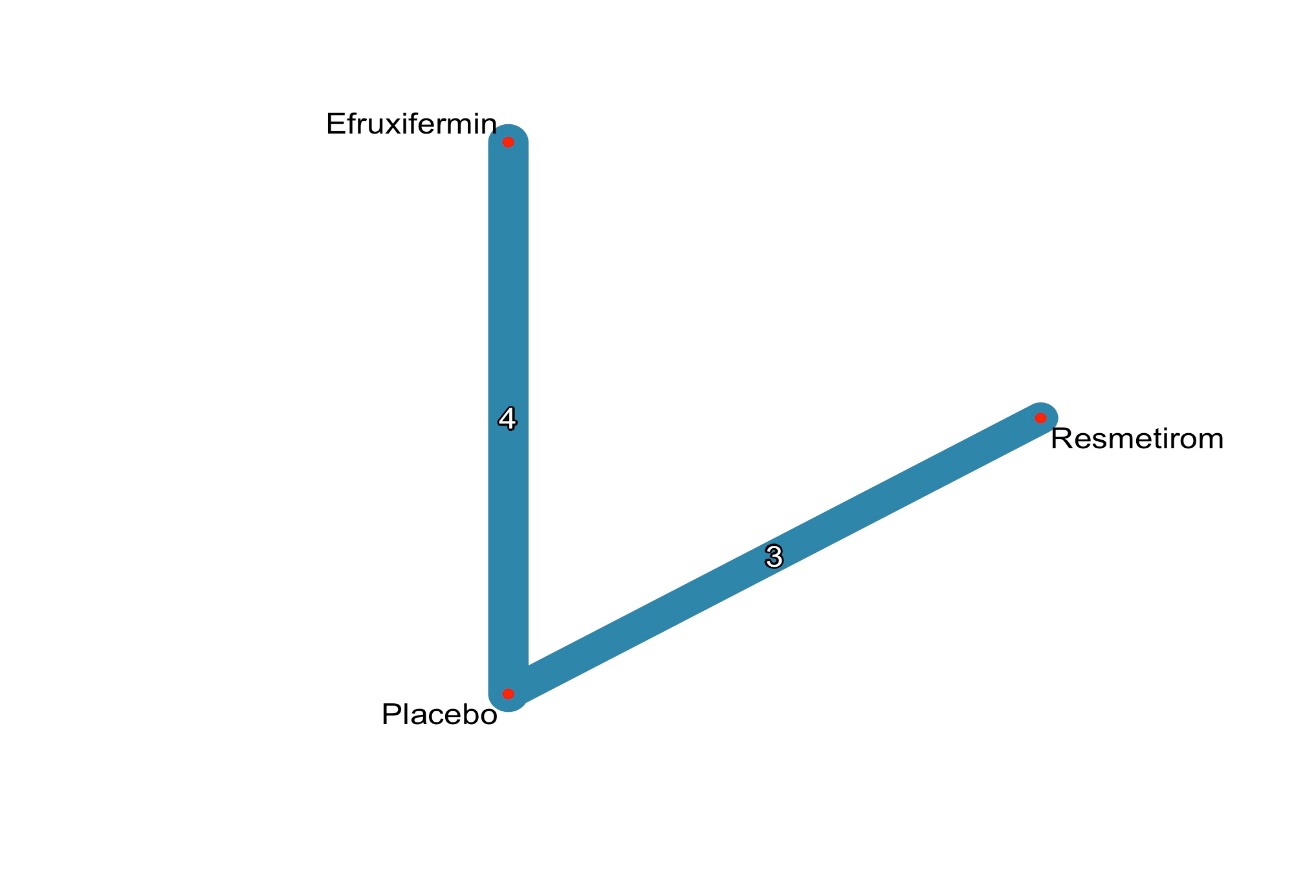


## Figure S8.8 Network Plot of Treatment Comparisons for Treatment Discontinuation


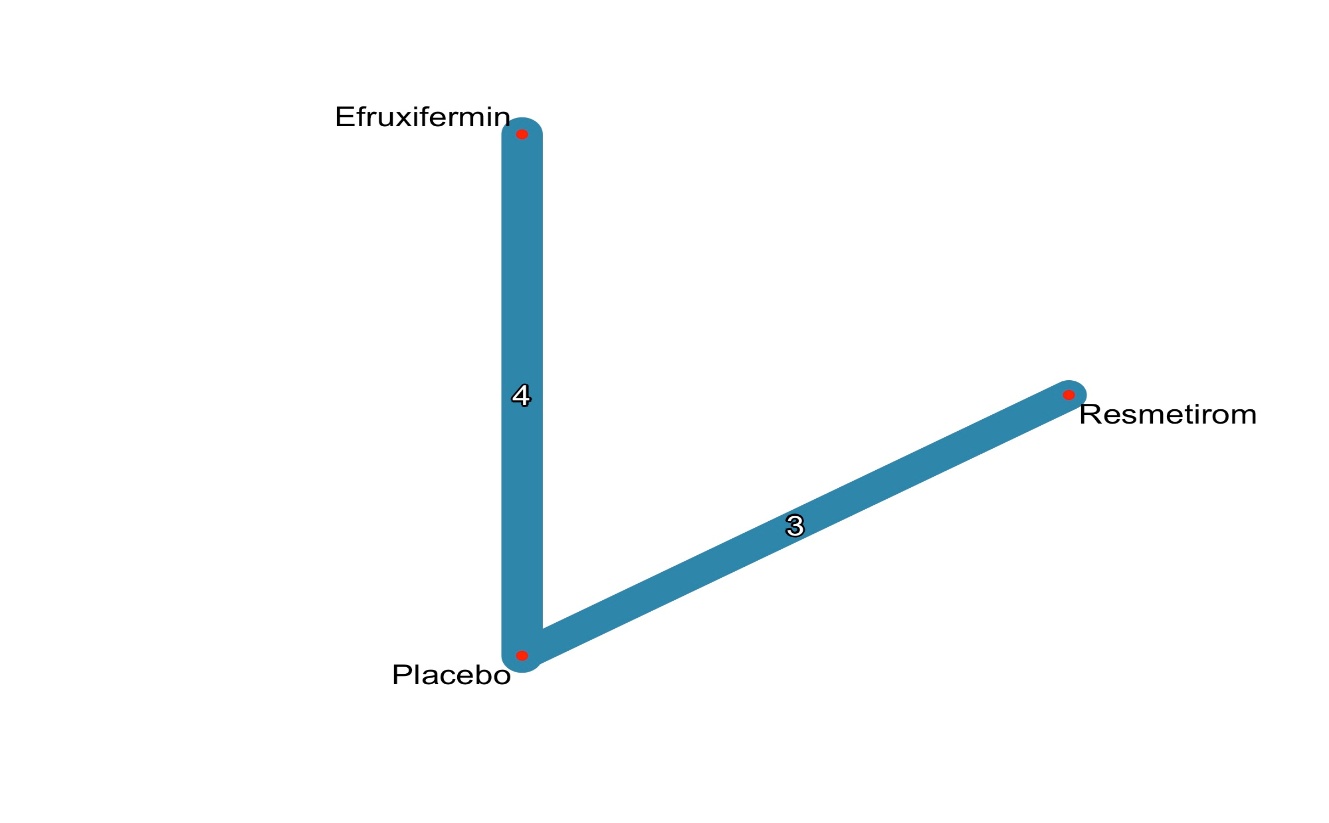


## Figure S8.9 Network Plot of Treatment Comparisons for Nausea


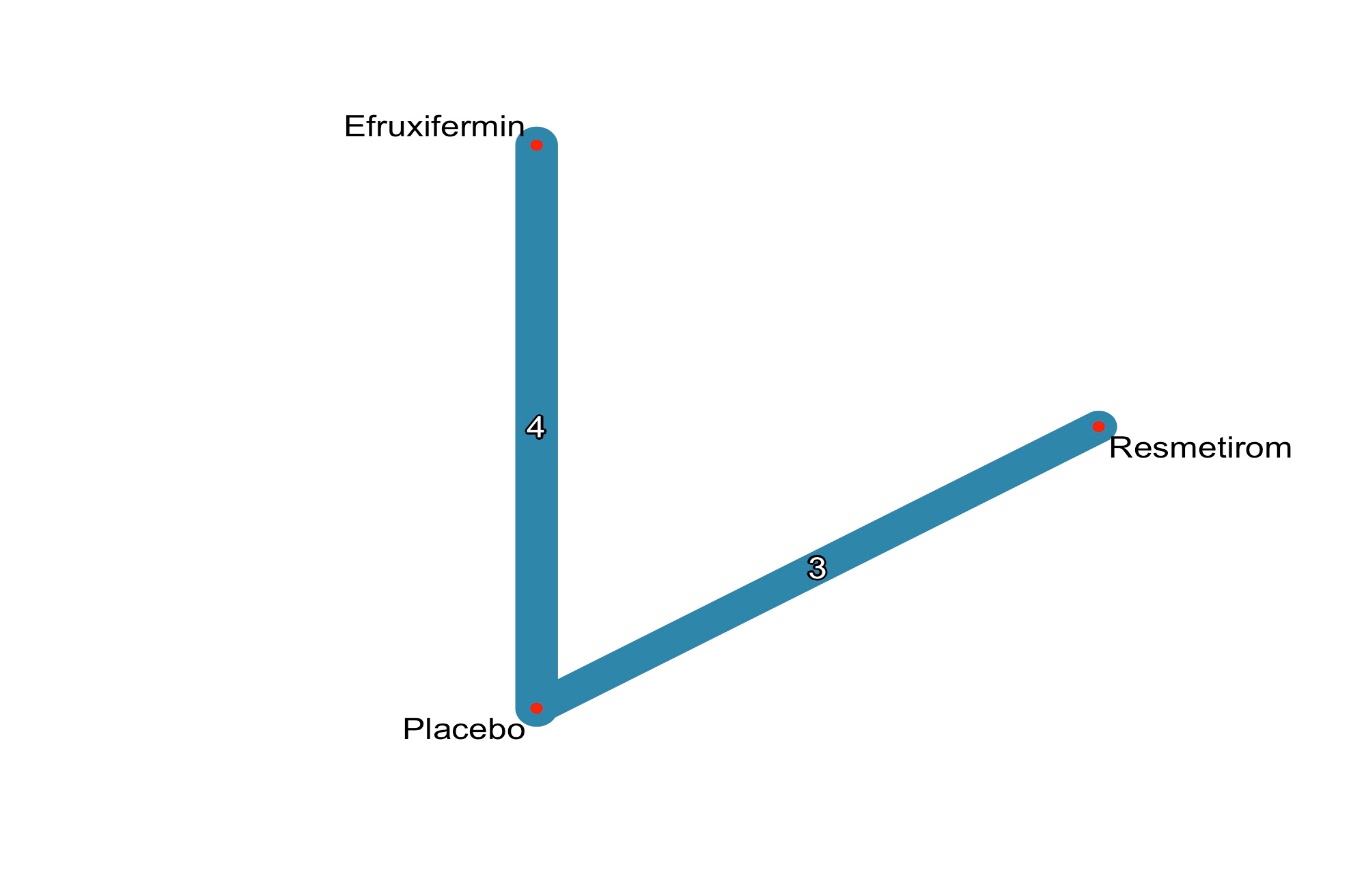


## Figure S8.10 Network Plot of Treatment Comparisons for Diarrhea


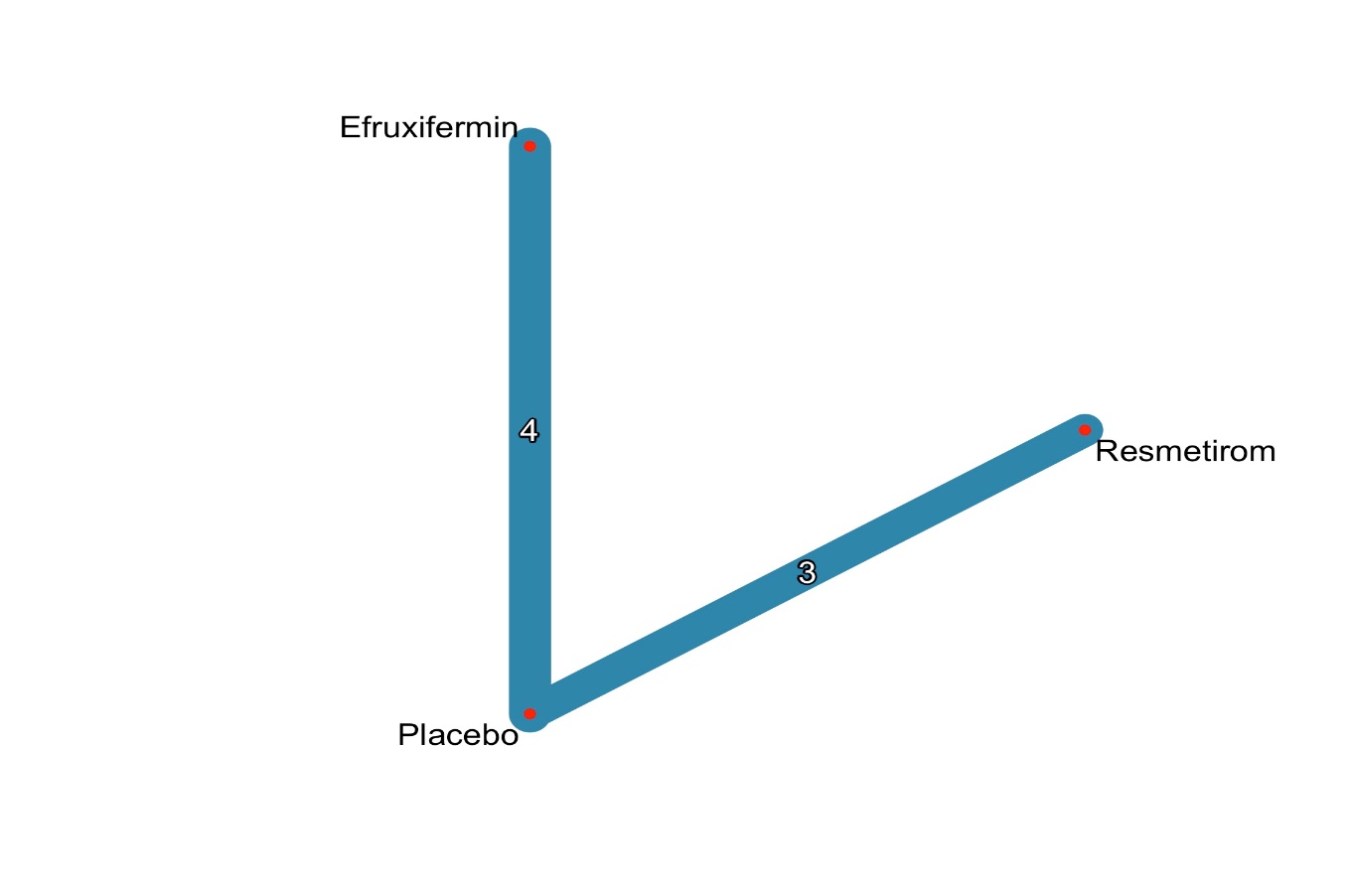


## Figure S8.11 Network Plot of Treatment Comparisons for Abdominal Pain


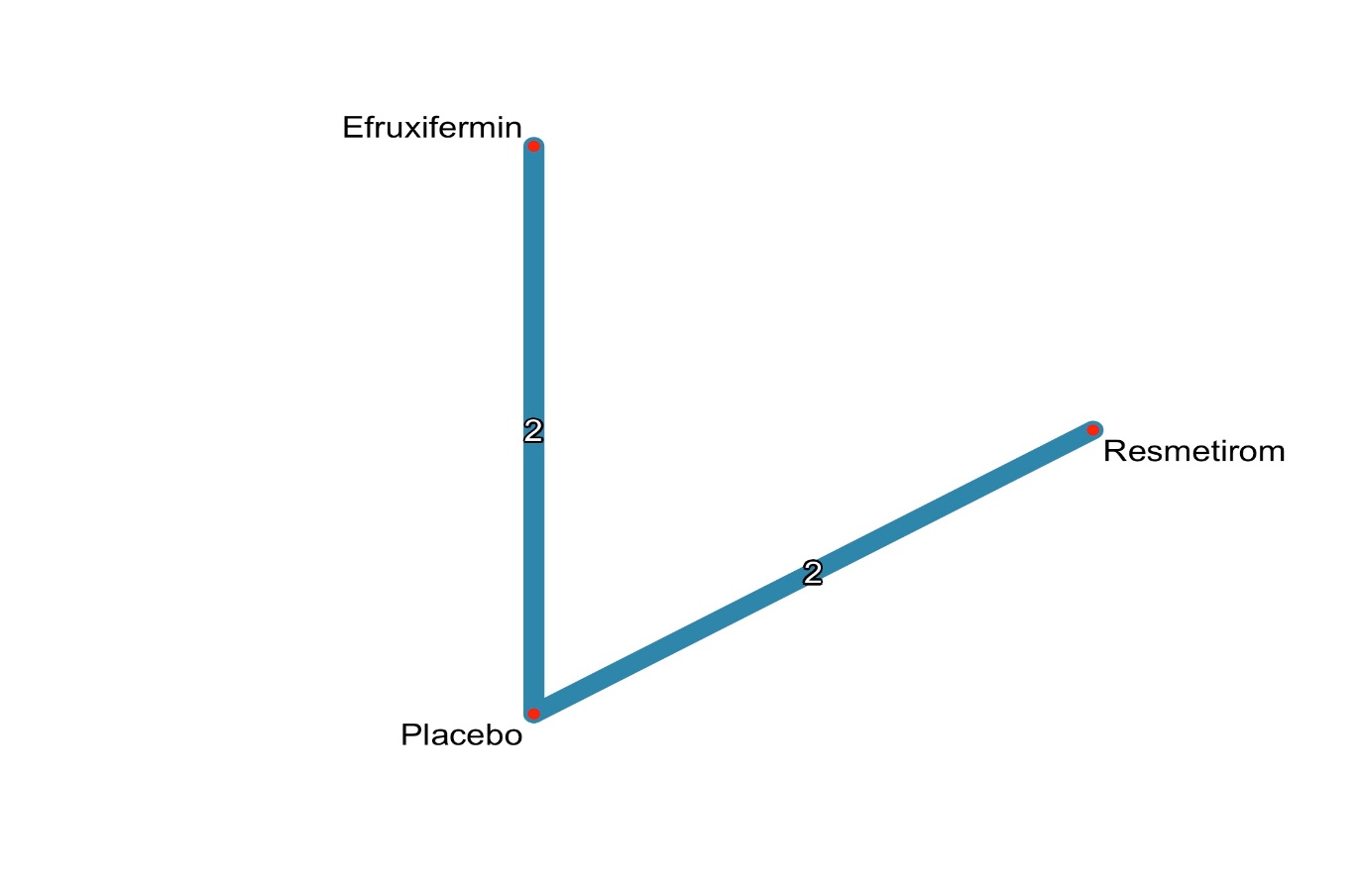


# Supplement S9: Summary of the excluded studies:

| Study | Reason for Exclusion |
| --- | --- |
| Younossi ZM, Stepanova M, Taub RA, Barbone JM, Harrison SA. Hepatic Fat Reduction Due to Resmetirom in Patients With Nonalcoholic Steatohepatitis Is Associated With Improvement of Quality of Life. Clin Gastroenterol Hepatol. 2022 Jun;20(6):1354-1361.e7. doi: 10.1016/j.cgh.2021.07.039. Epub 2021 Jul 27. PMID: 34329774. | Lack of outcome data |

# Supplement S10: Meta-regression:

## Figure S10.2 Percentage Change in MRI-PDFF


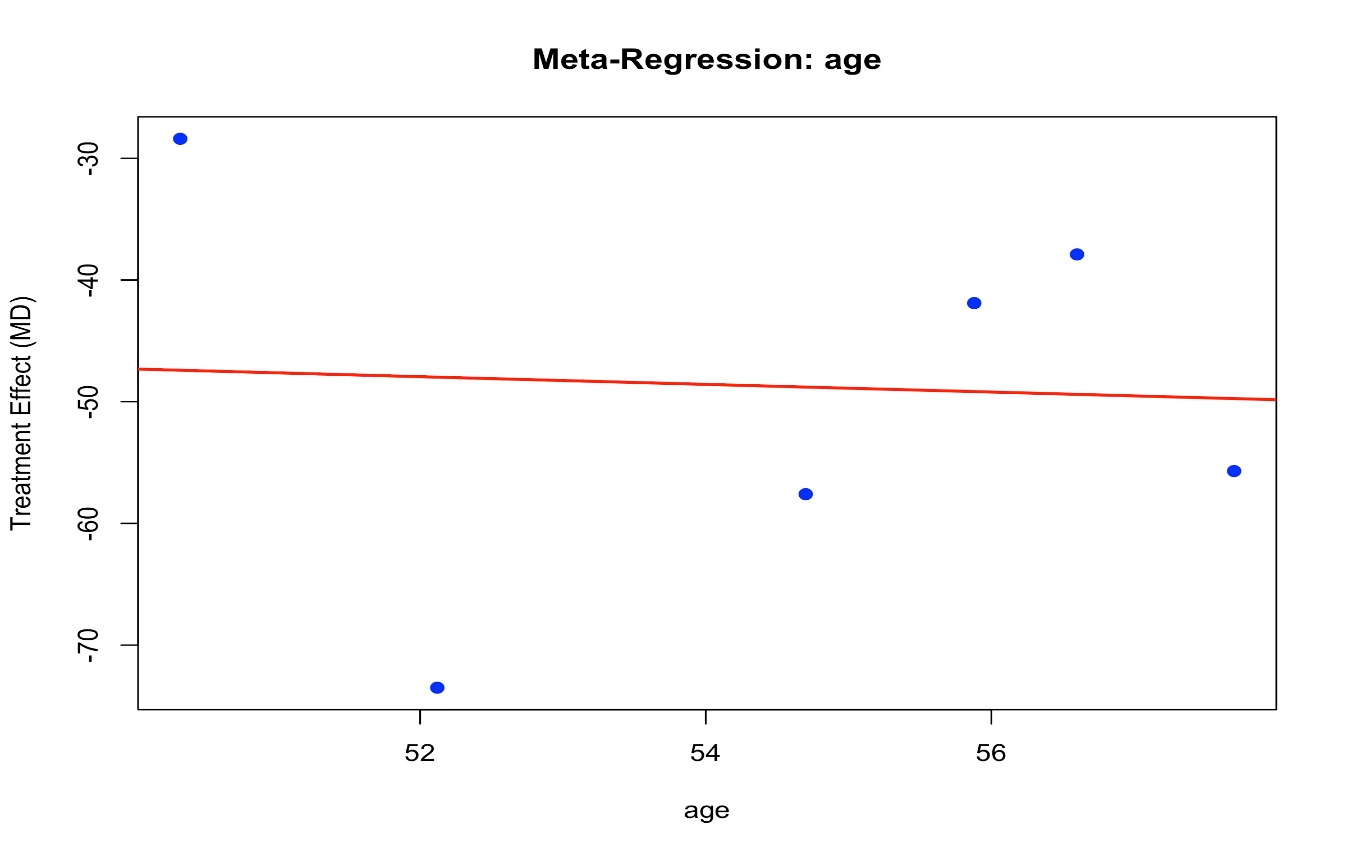


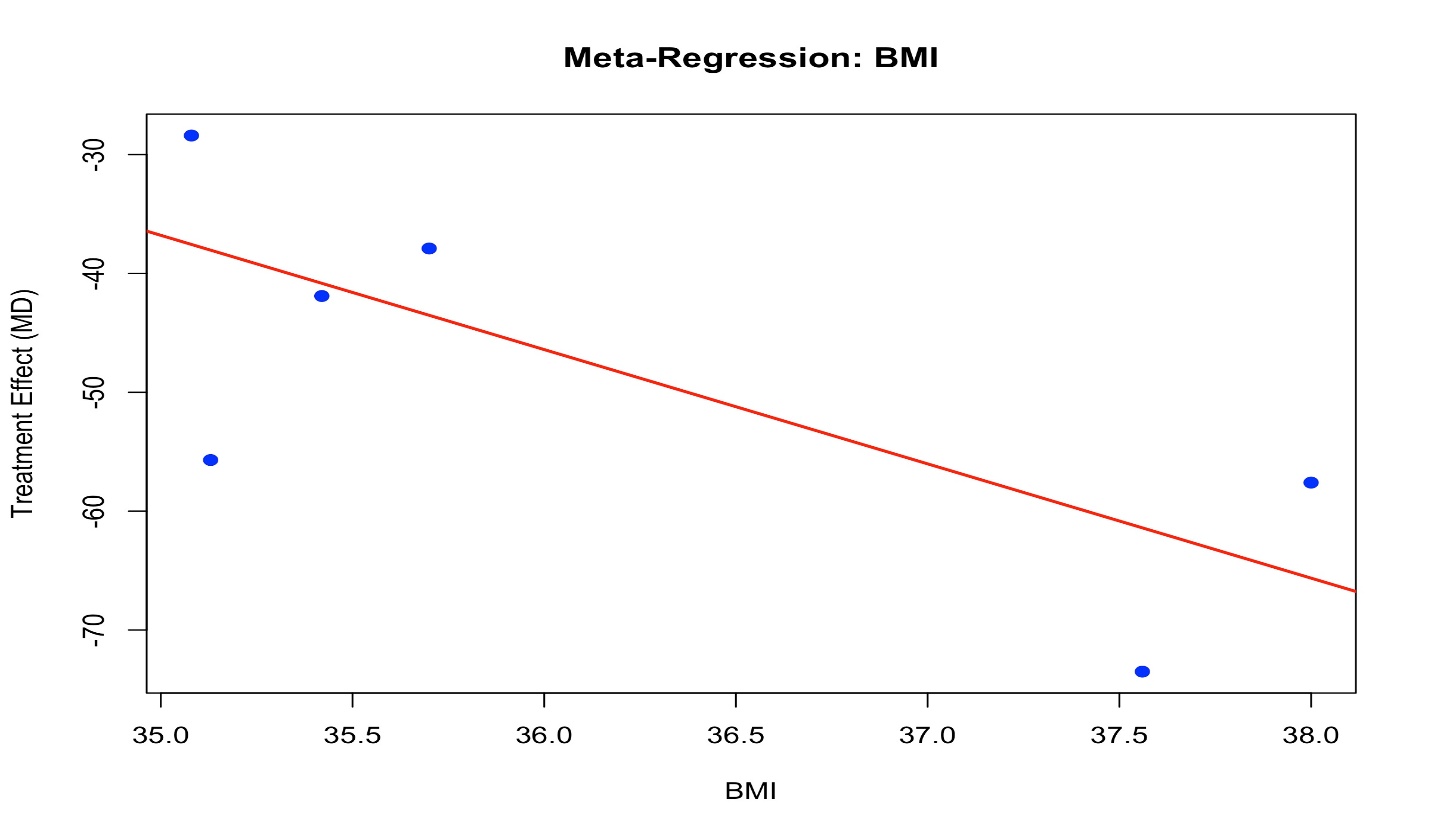


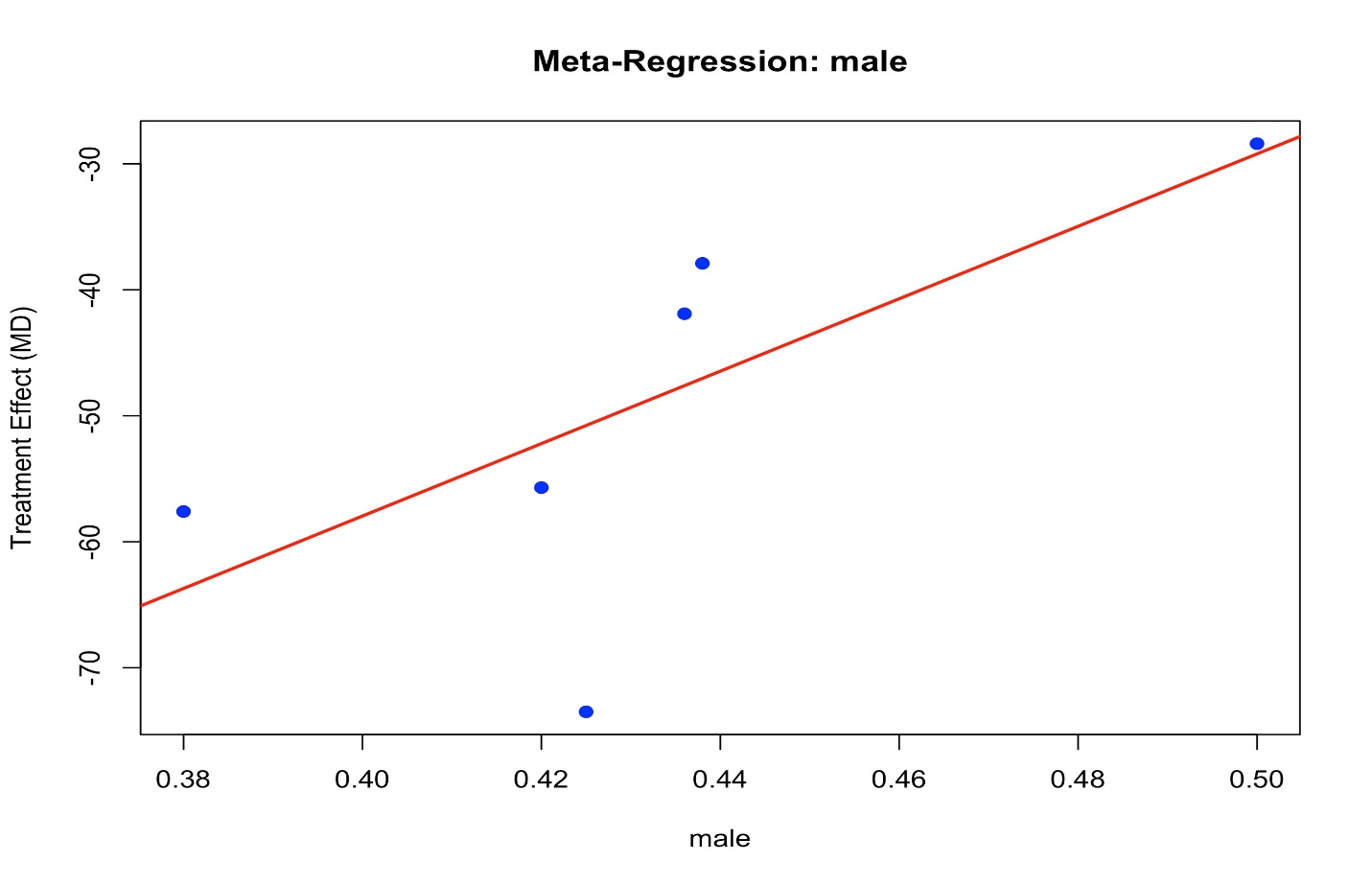

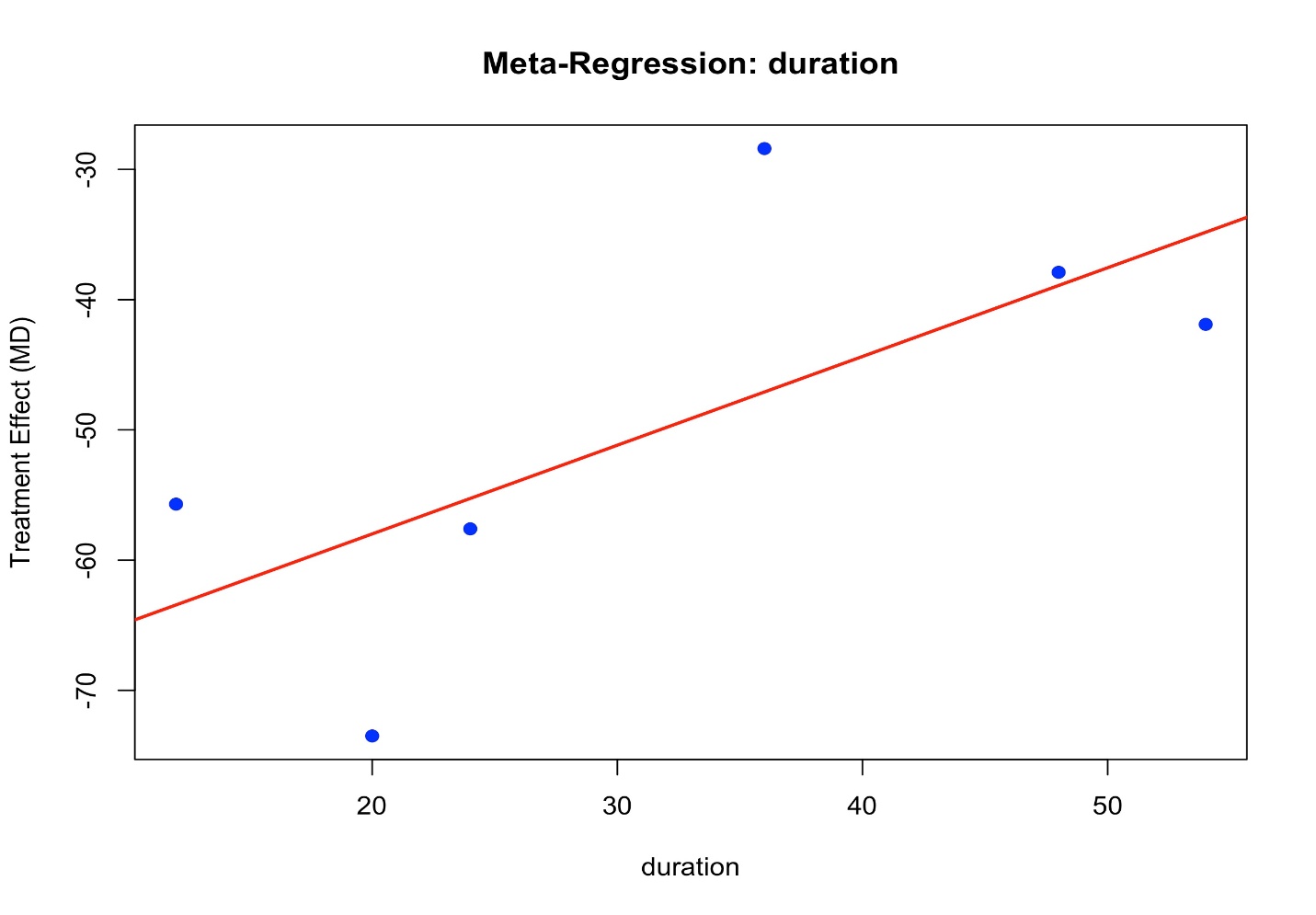


## Figure S10.3 Change in ALT


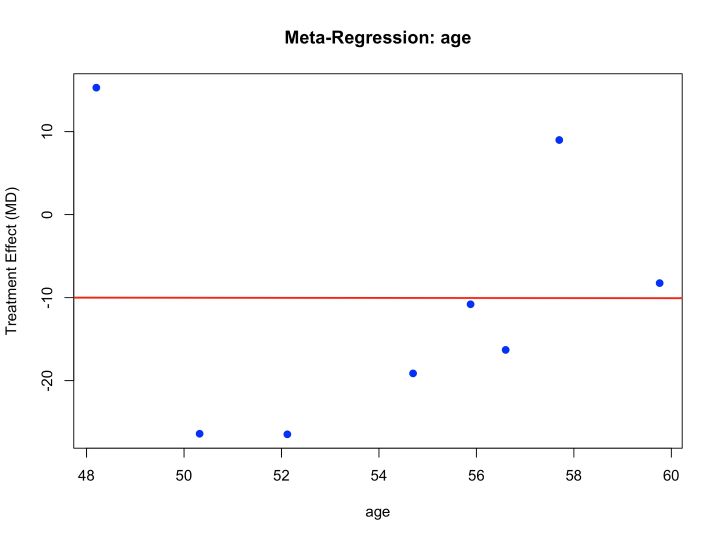


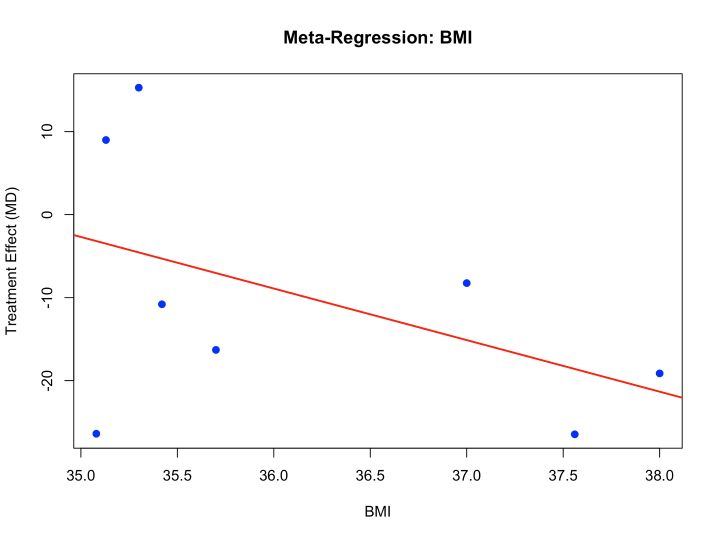


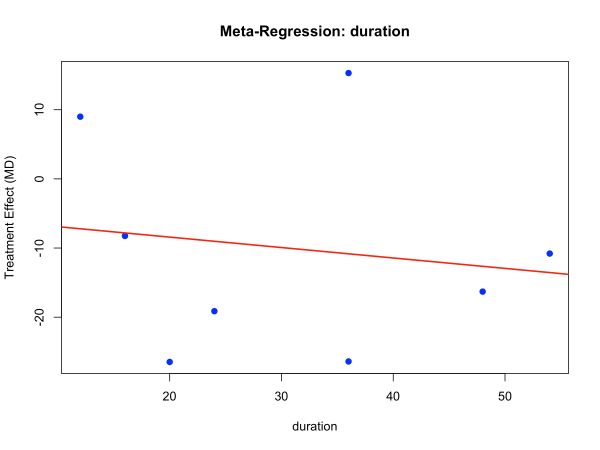


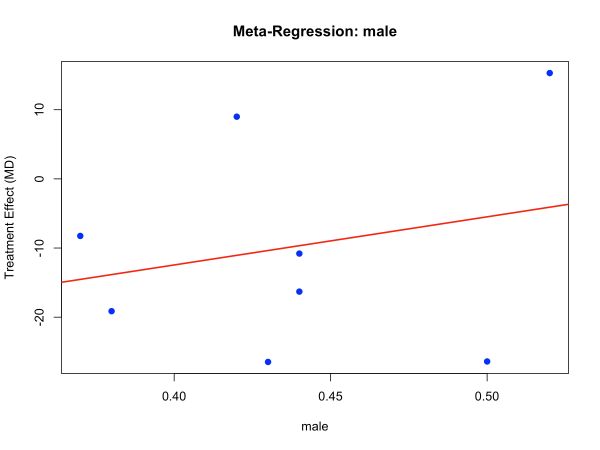


## Figure S10.4 Change in AST


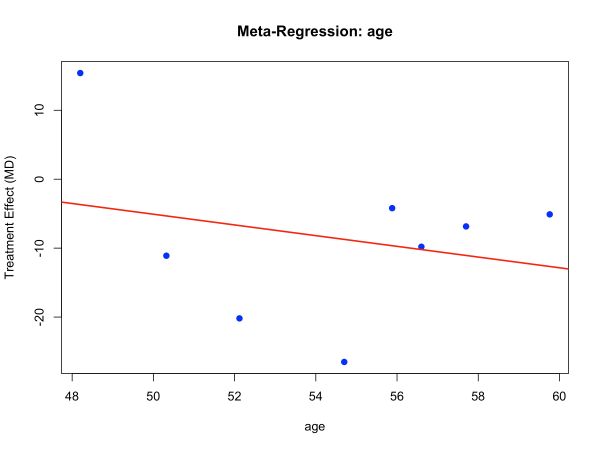


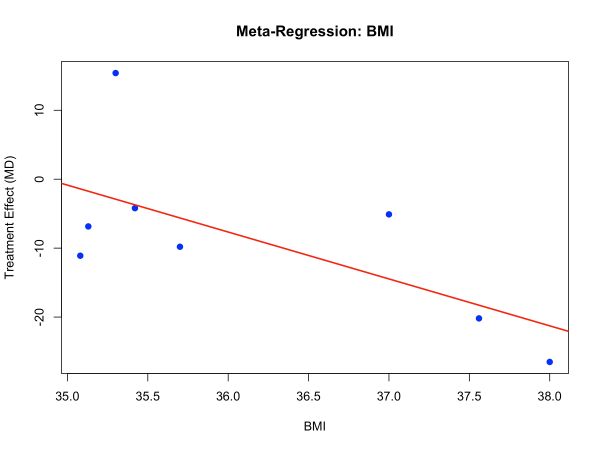


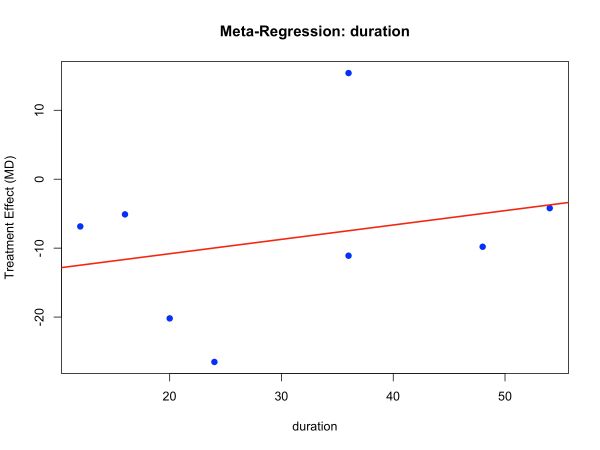


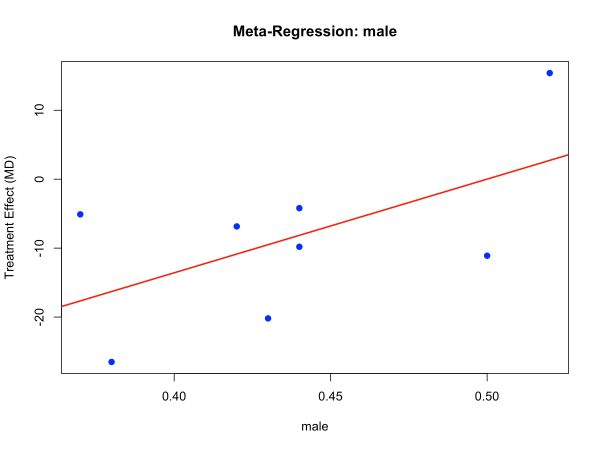


## Figure S10.5 Percentage Change in Triglyceride


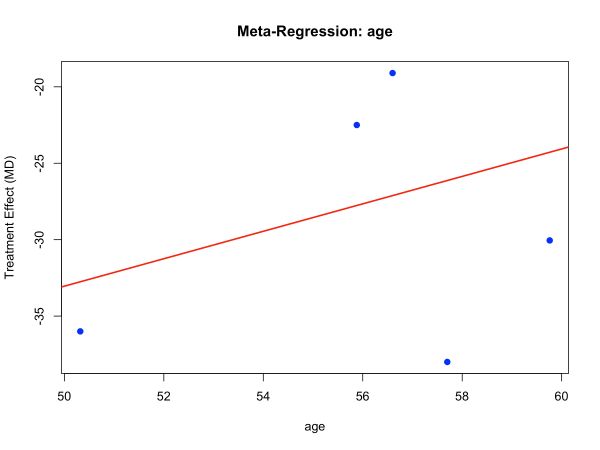


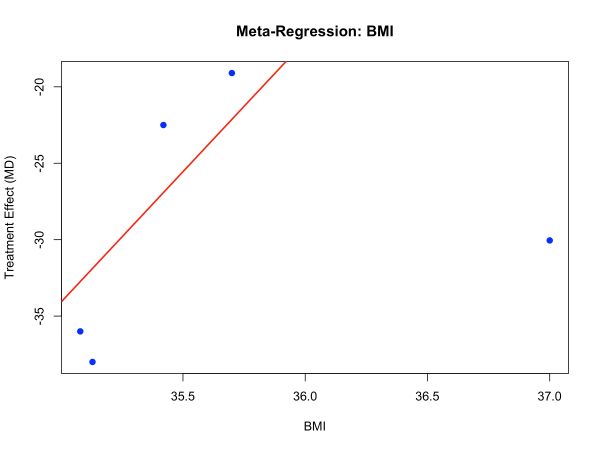


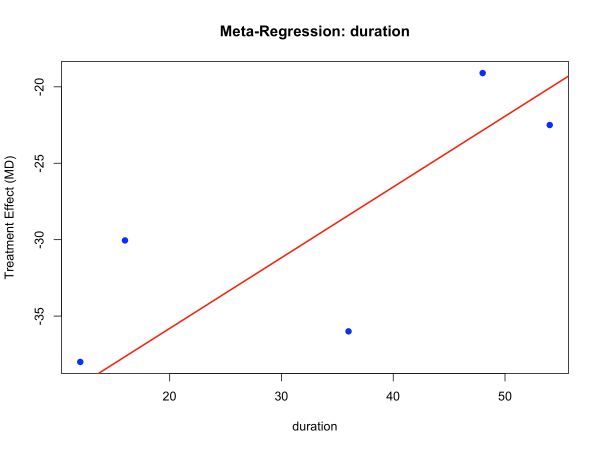


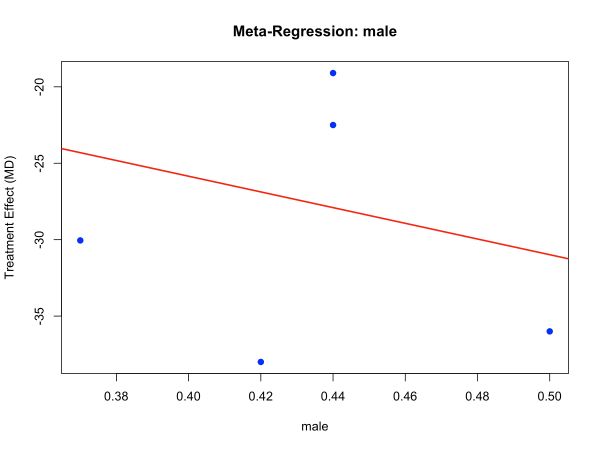


## Figure S10.6 Percentage Change in LDL


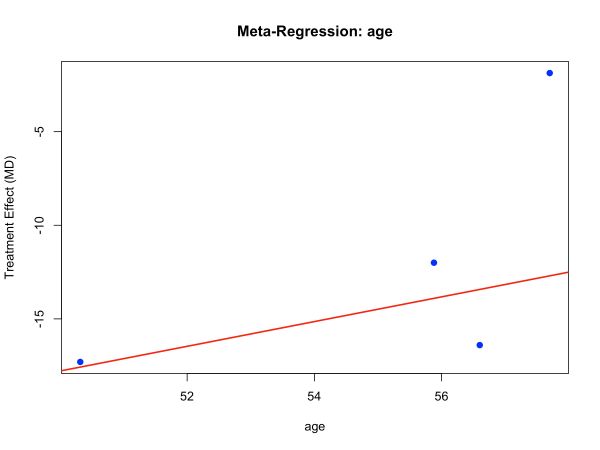


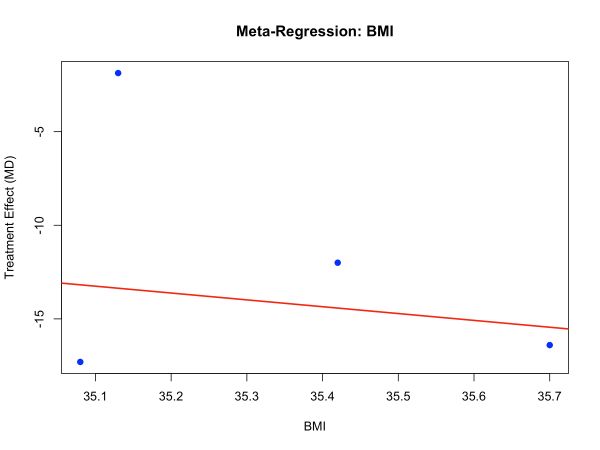


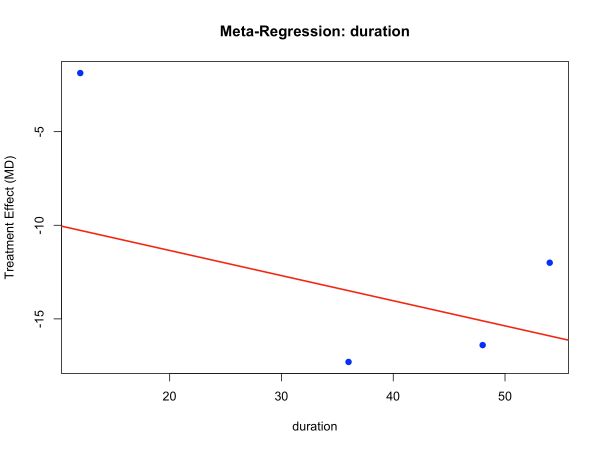


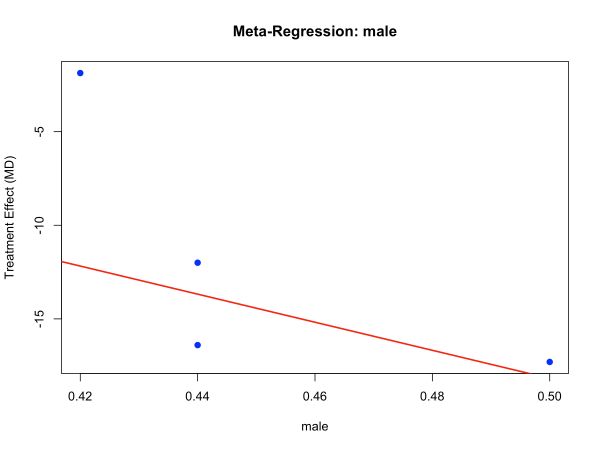


## Figure S10.7 Percentage Change in HDL


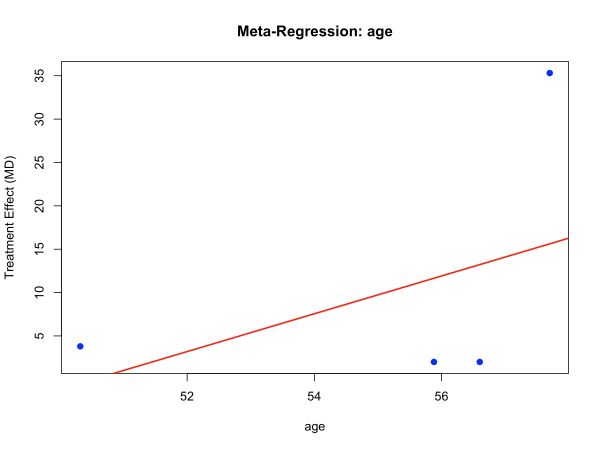


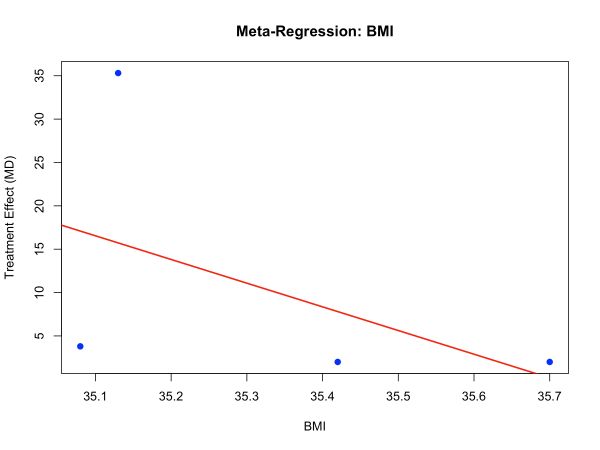


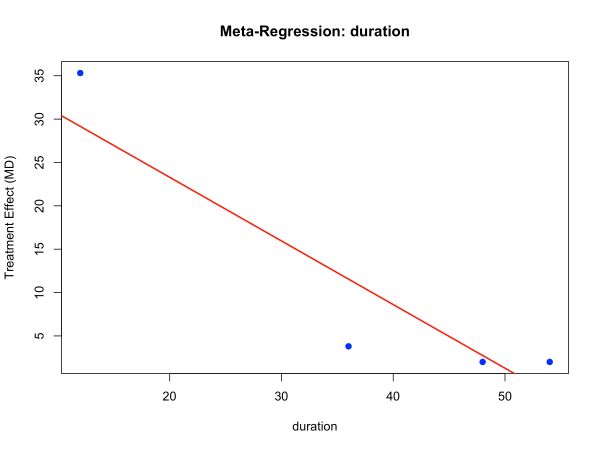


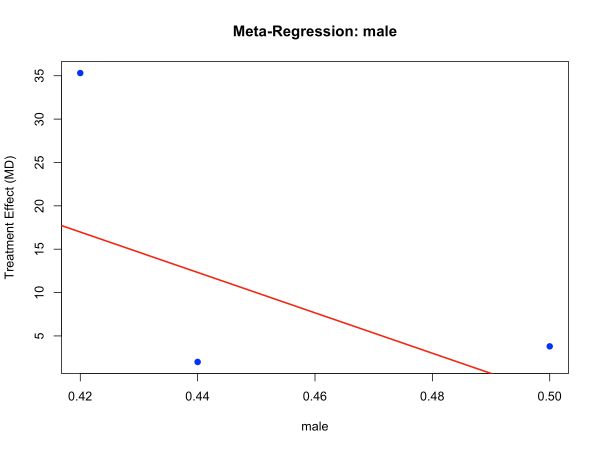


## Figure S10.8 Serious adverse events


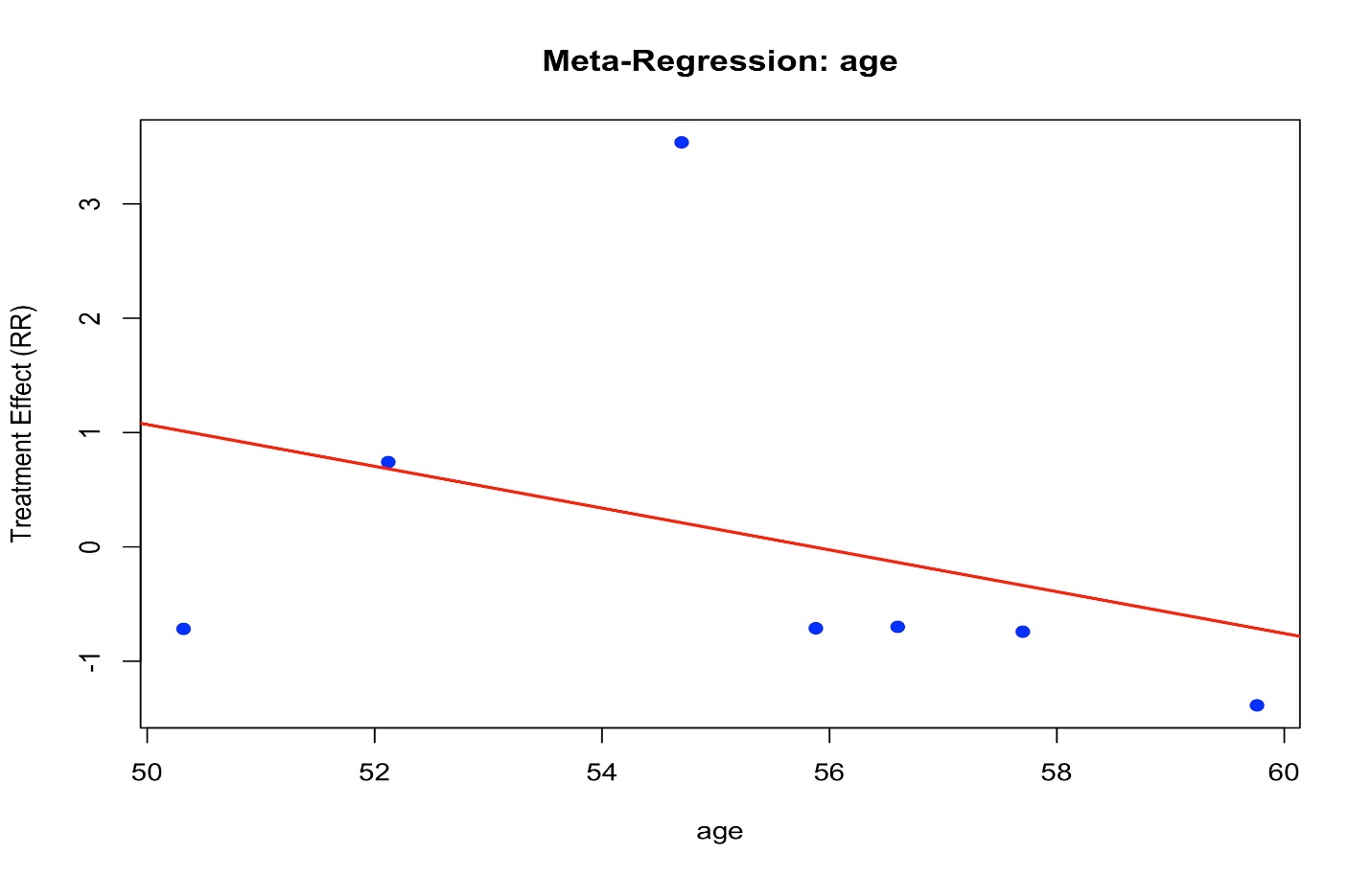


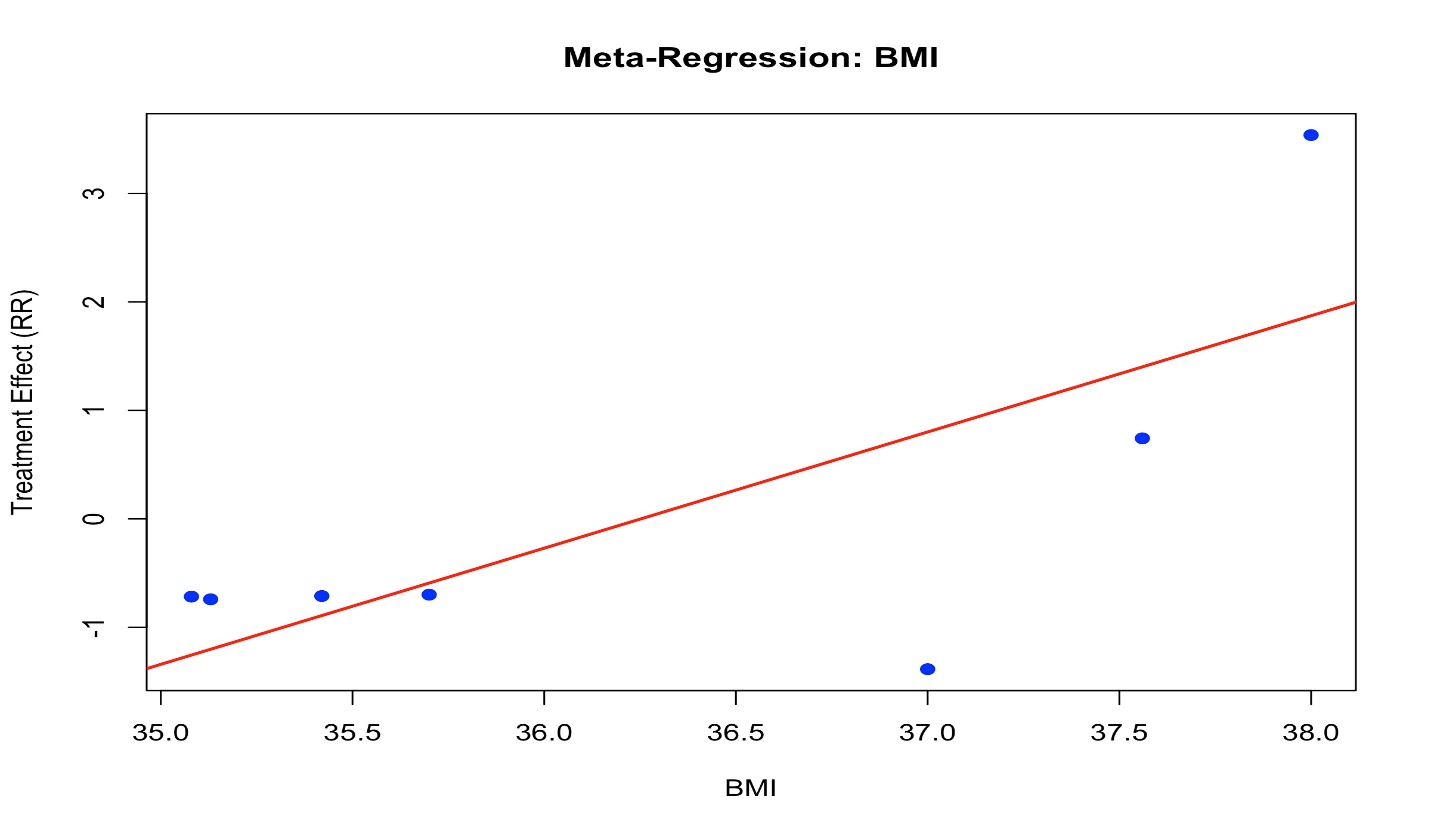


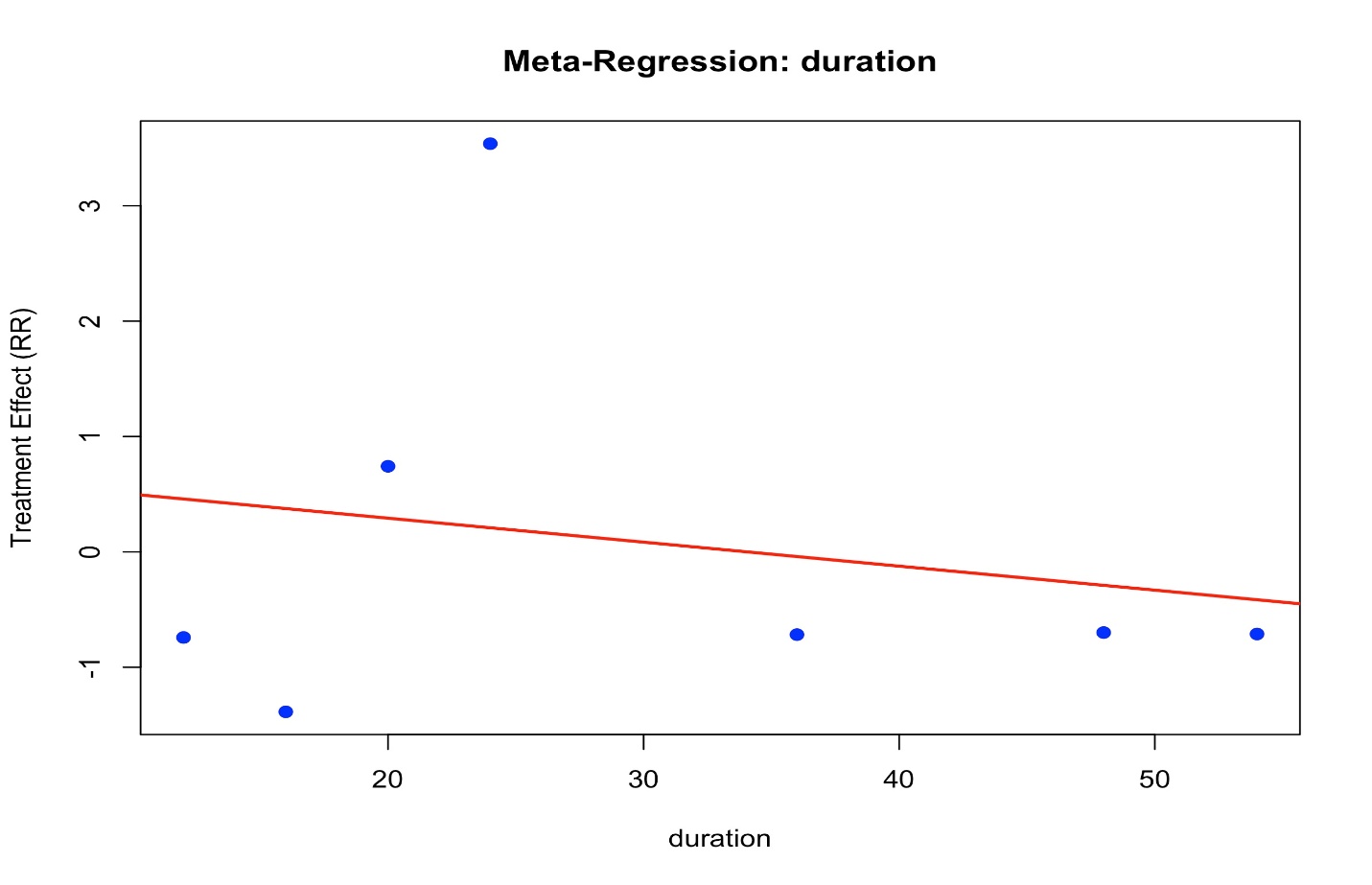


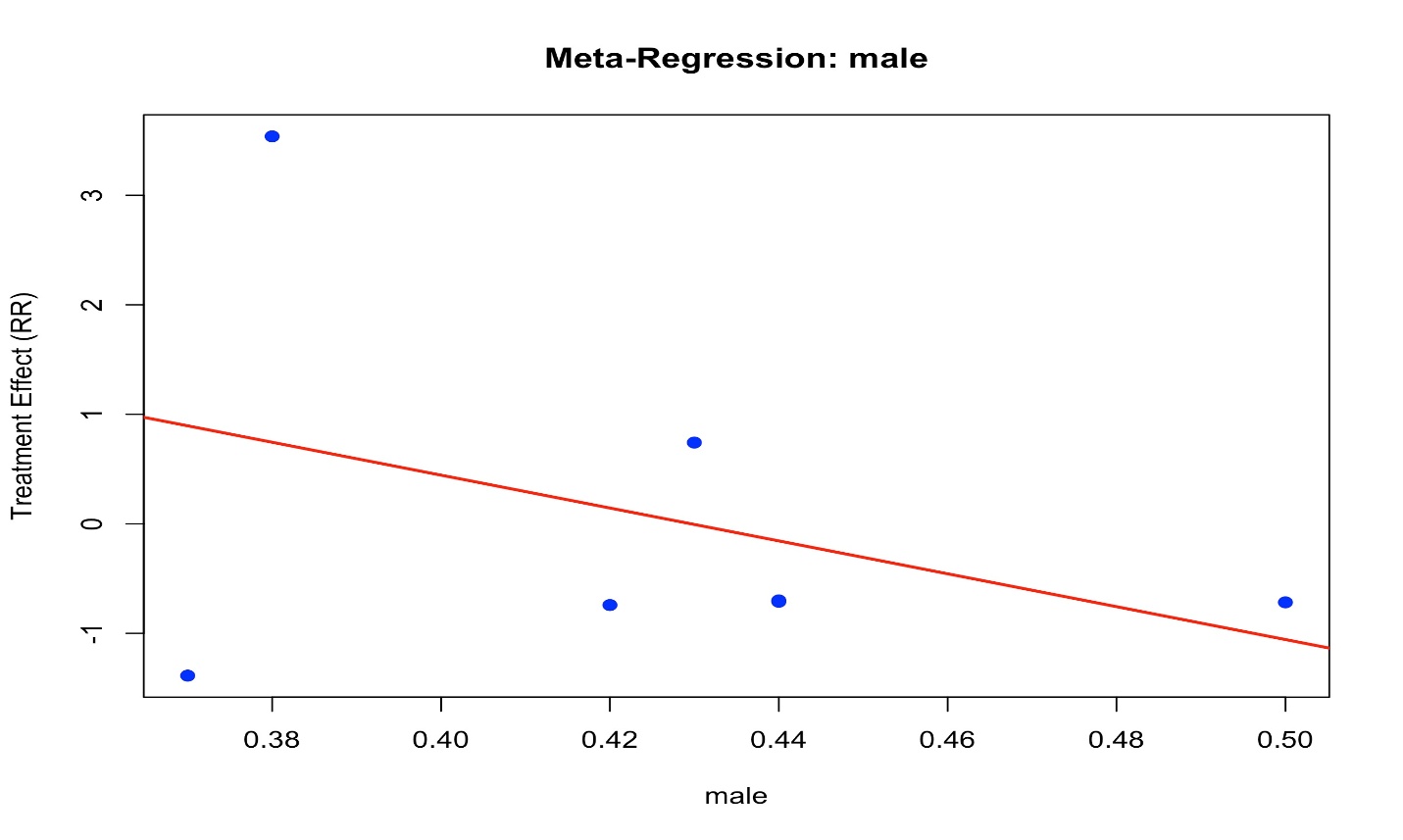


## Figure S10.9 Treatment Discontinuation


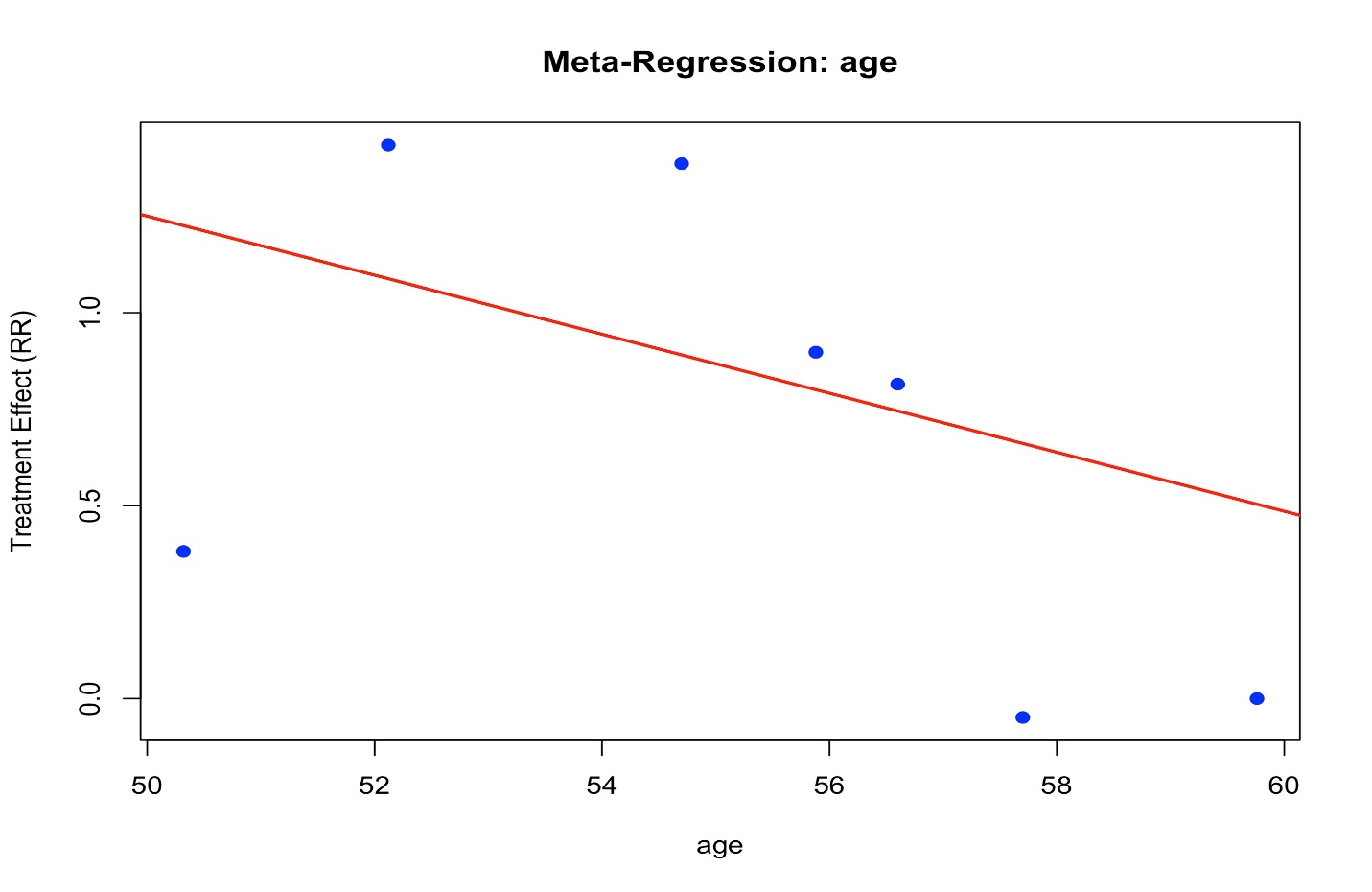


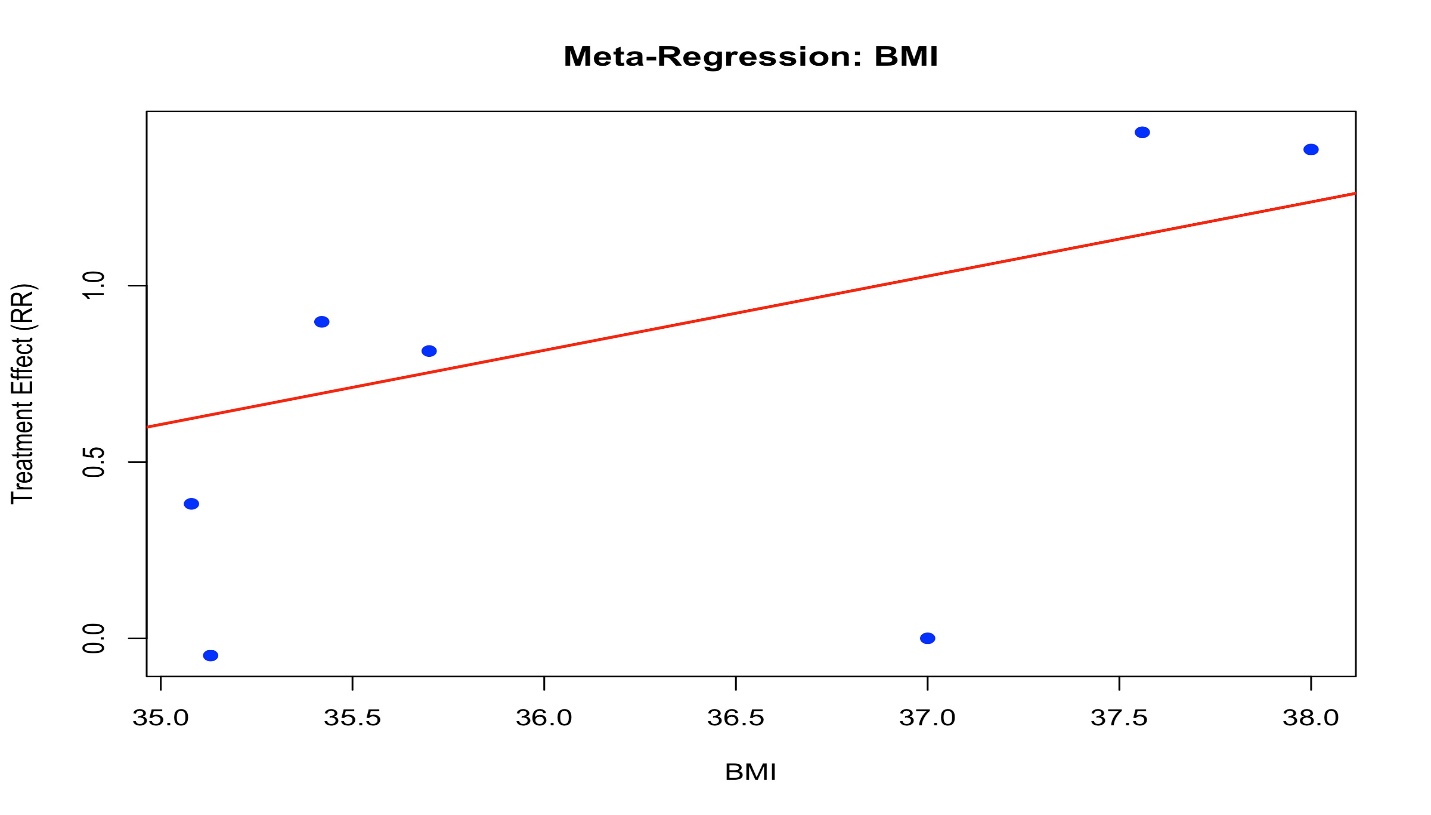


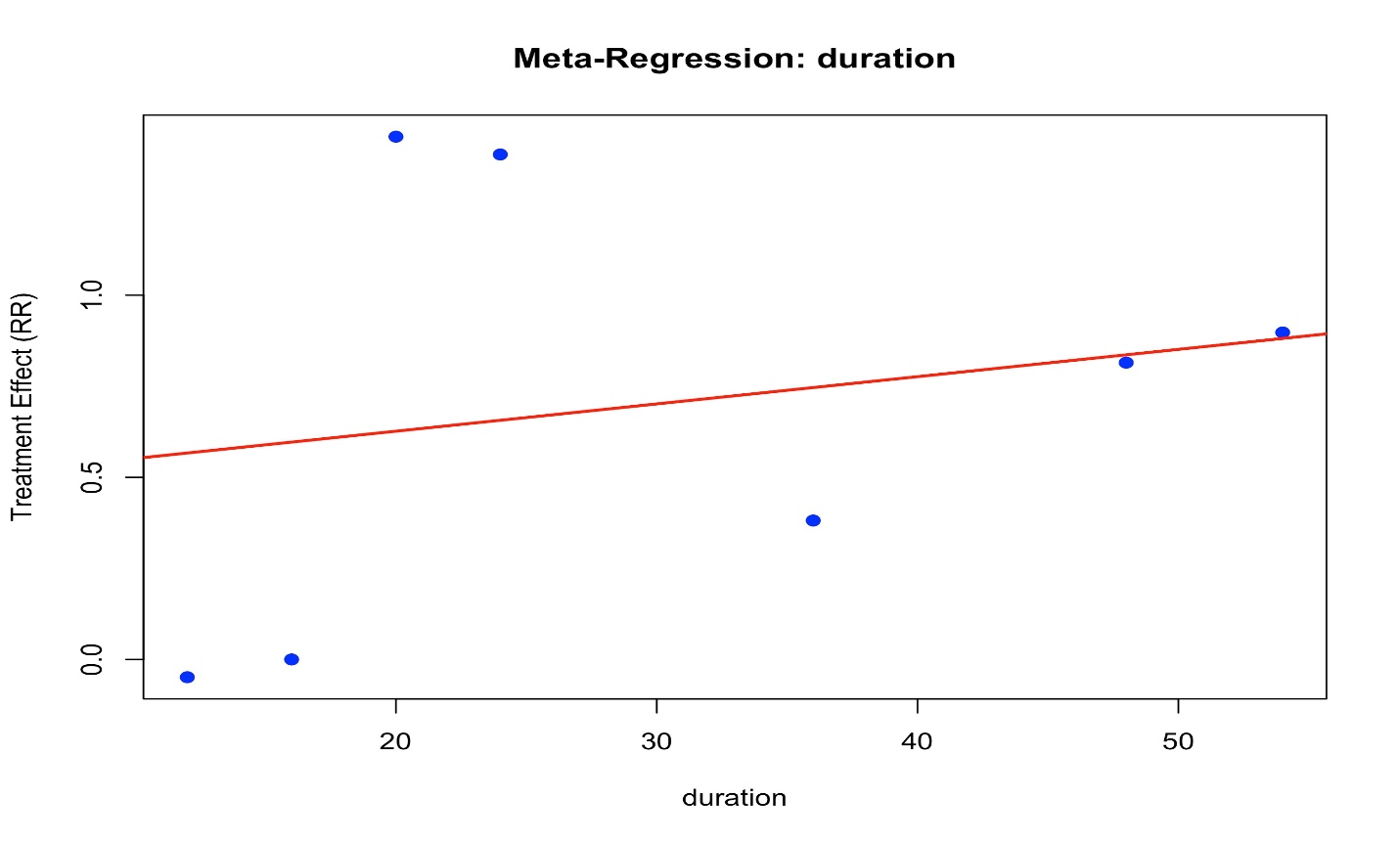


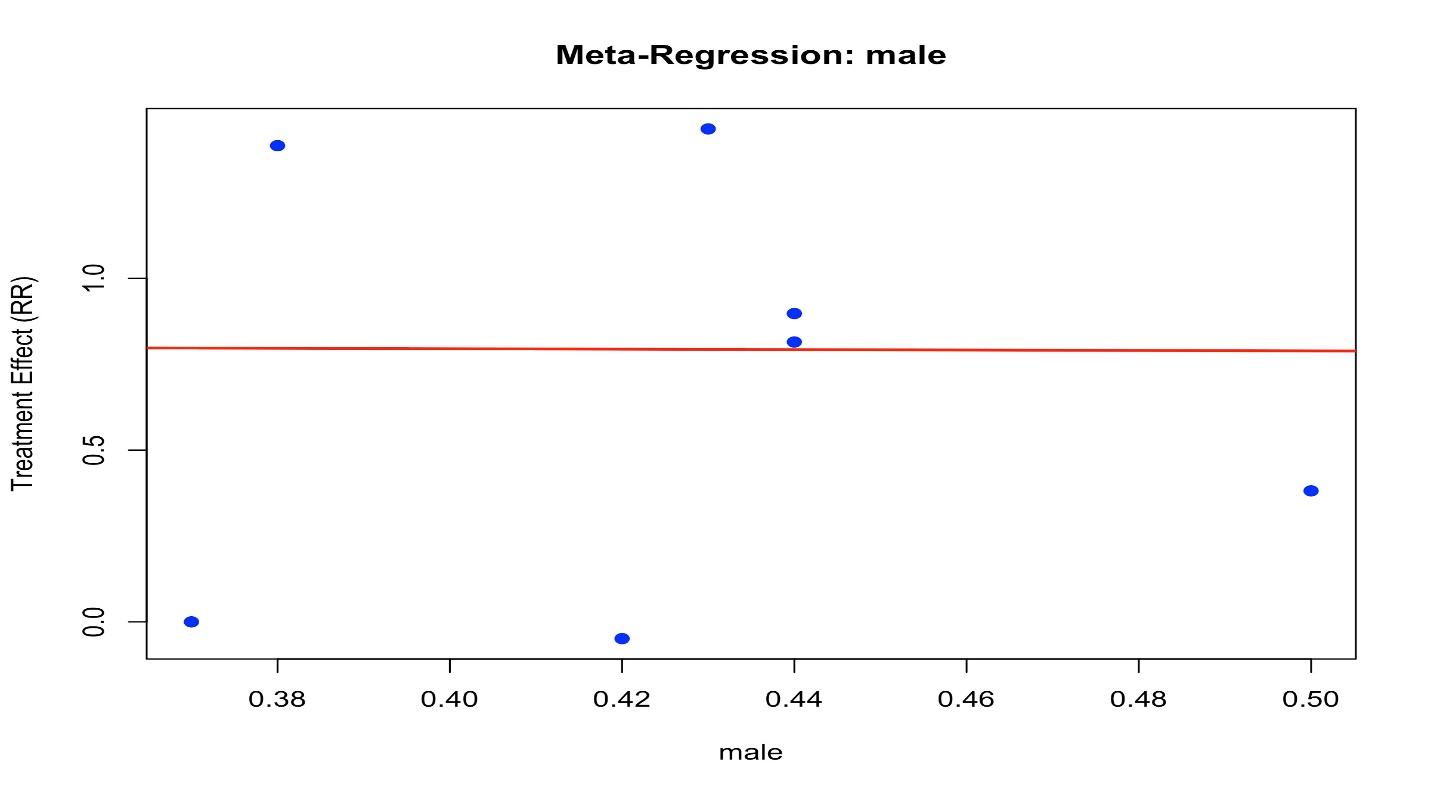


## Figure S10.10 Nausea


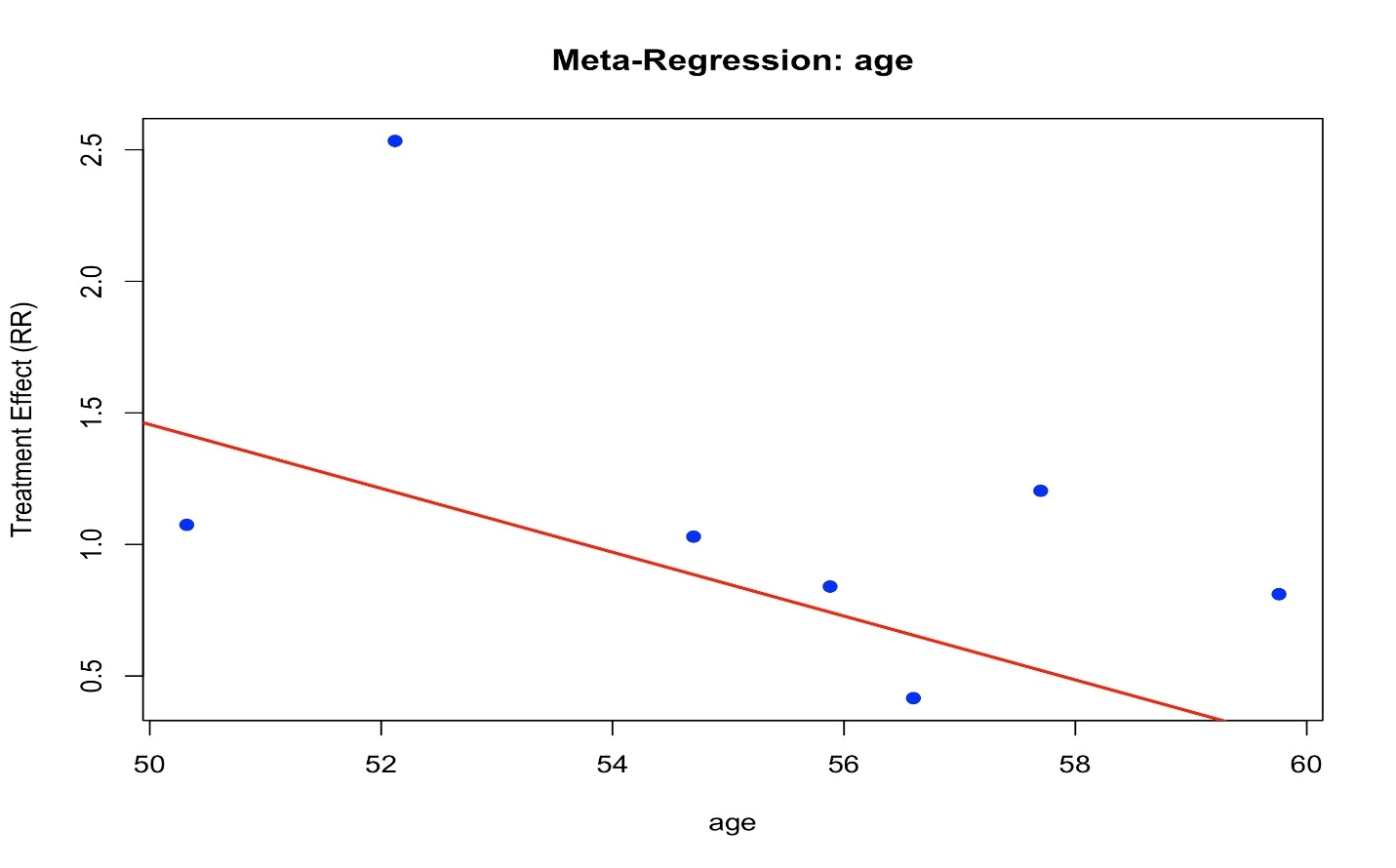


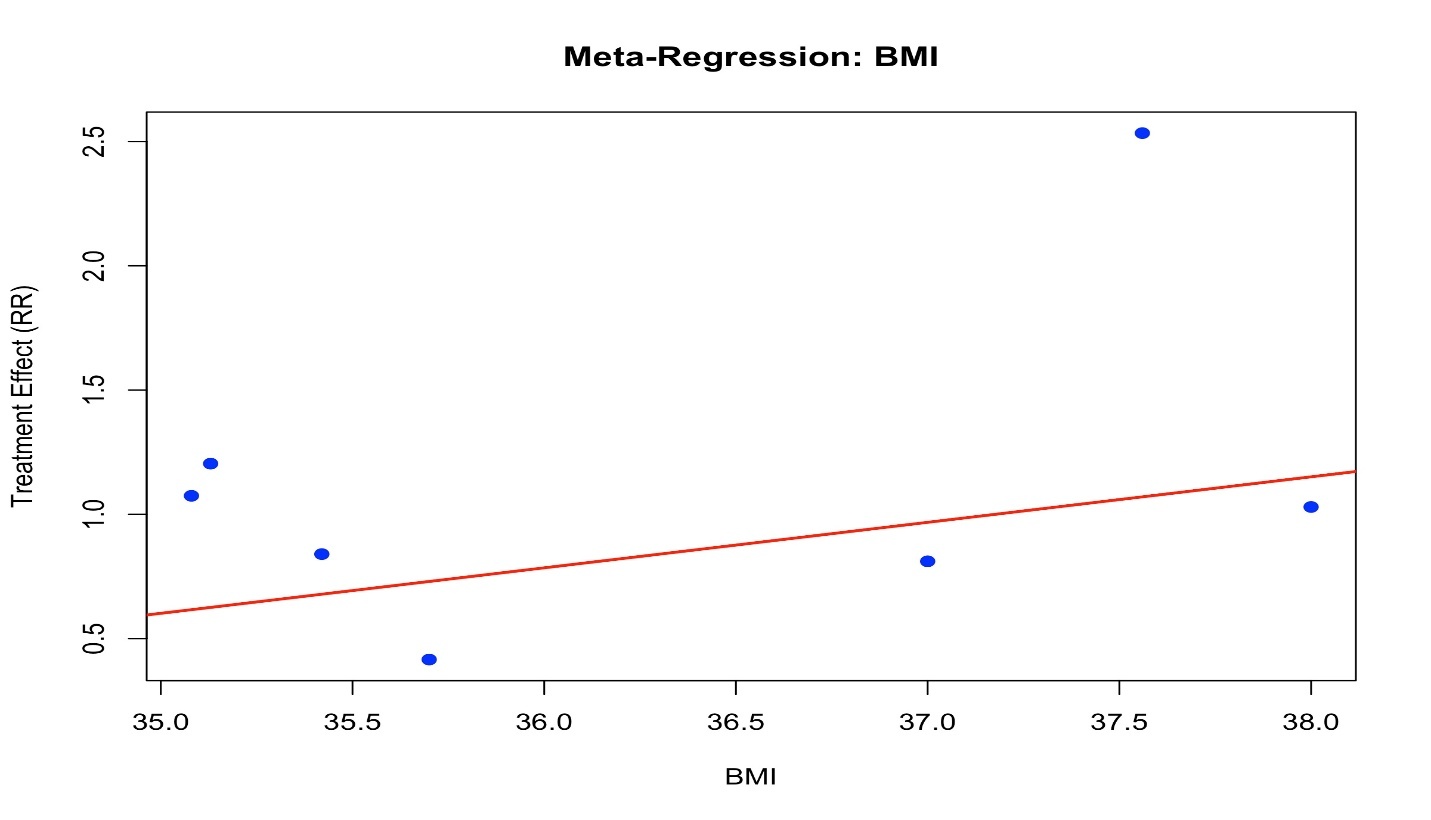


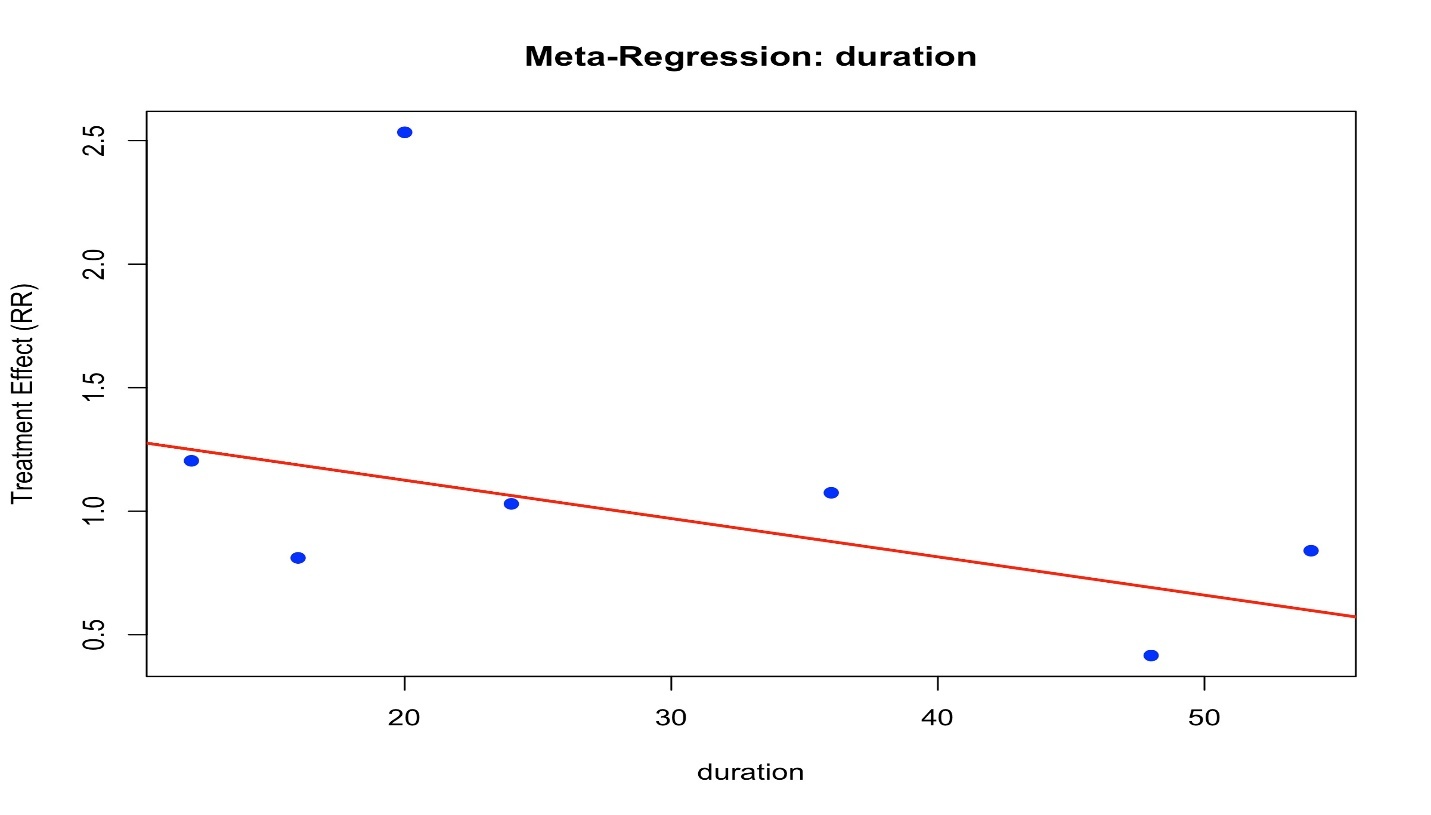


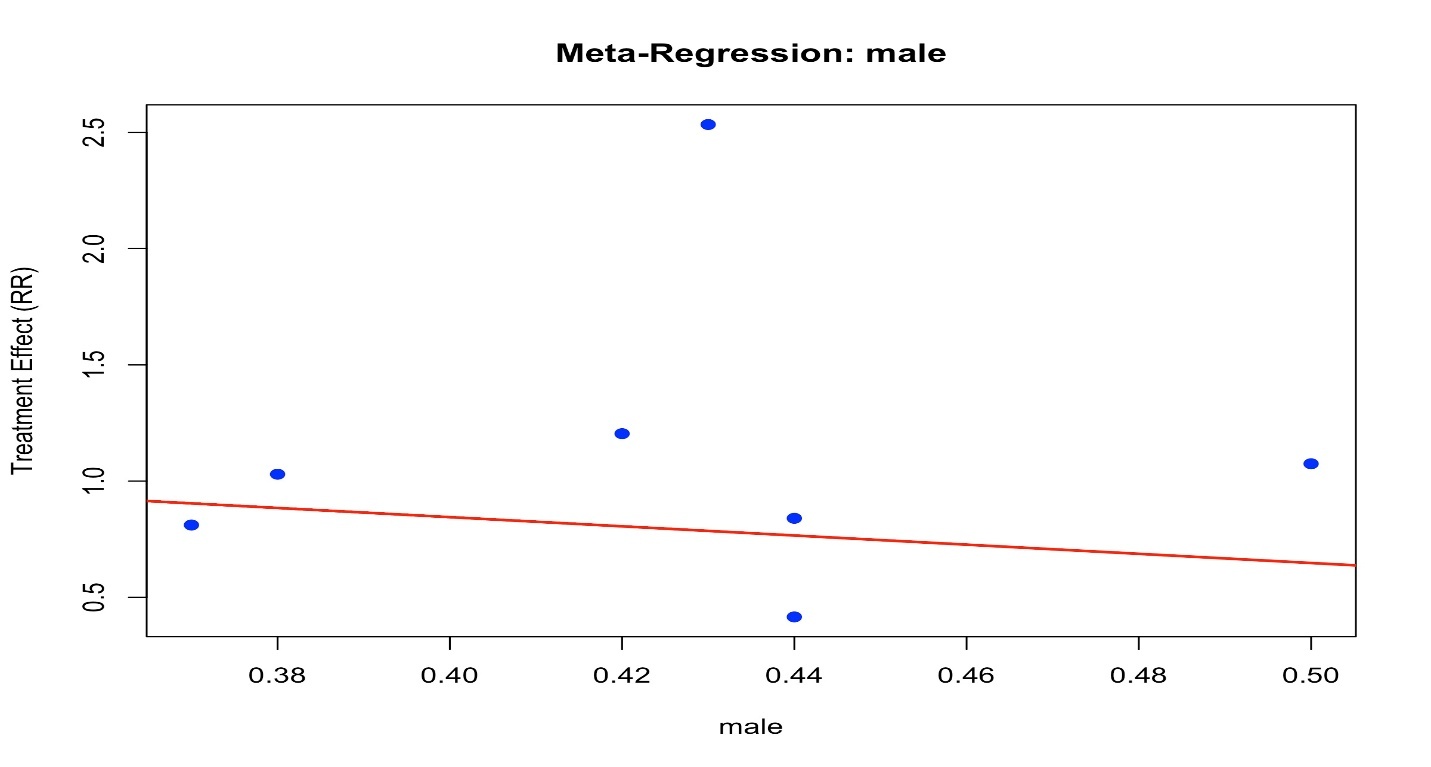


## Figure S10.11 Diarrhea


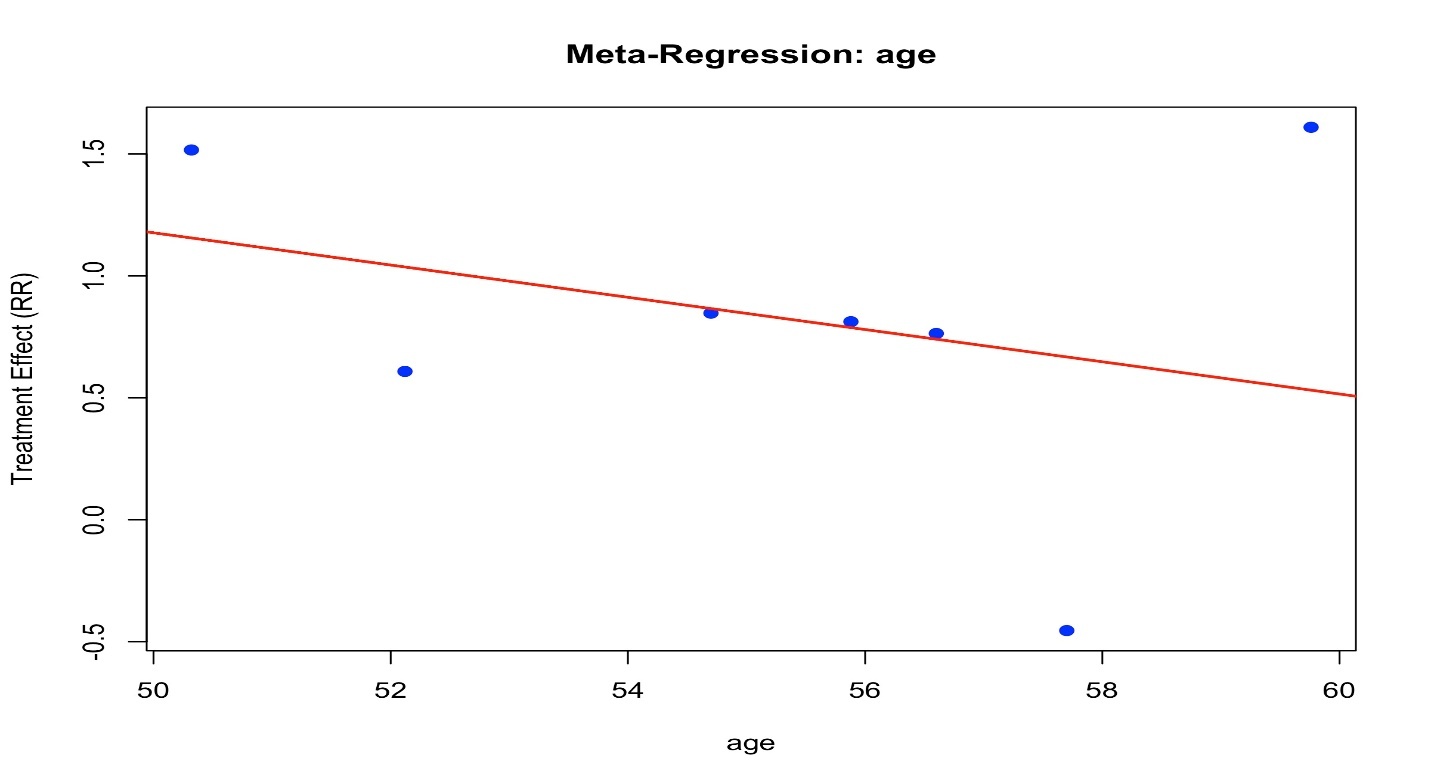


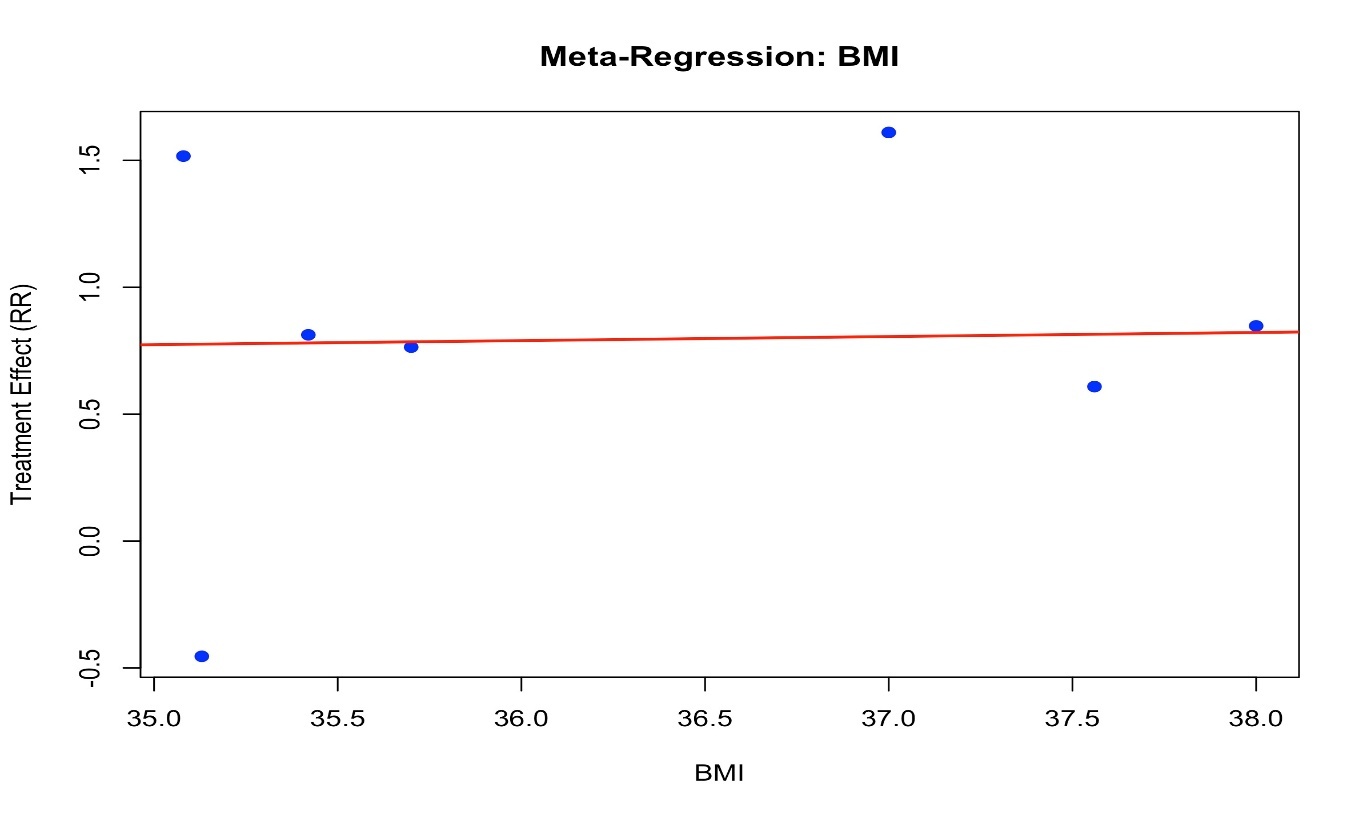


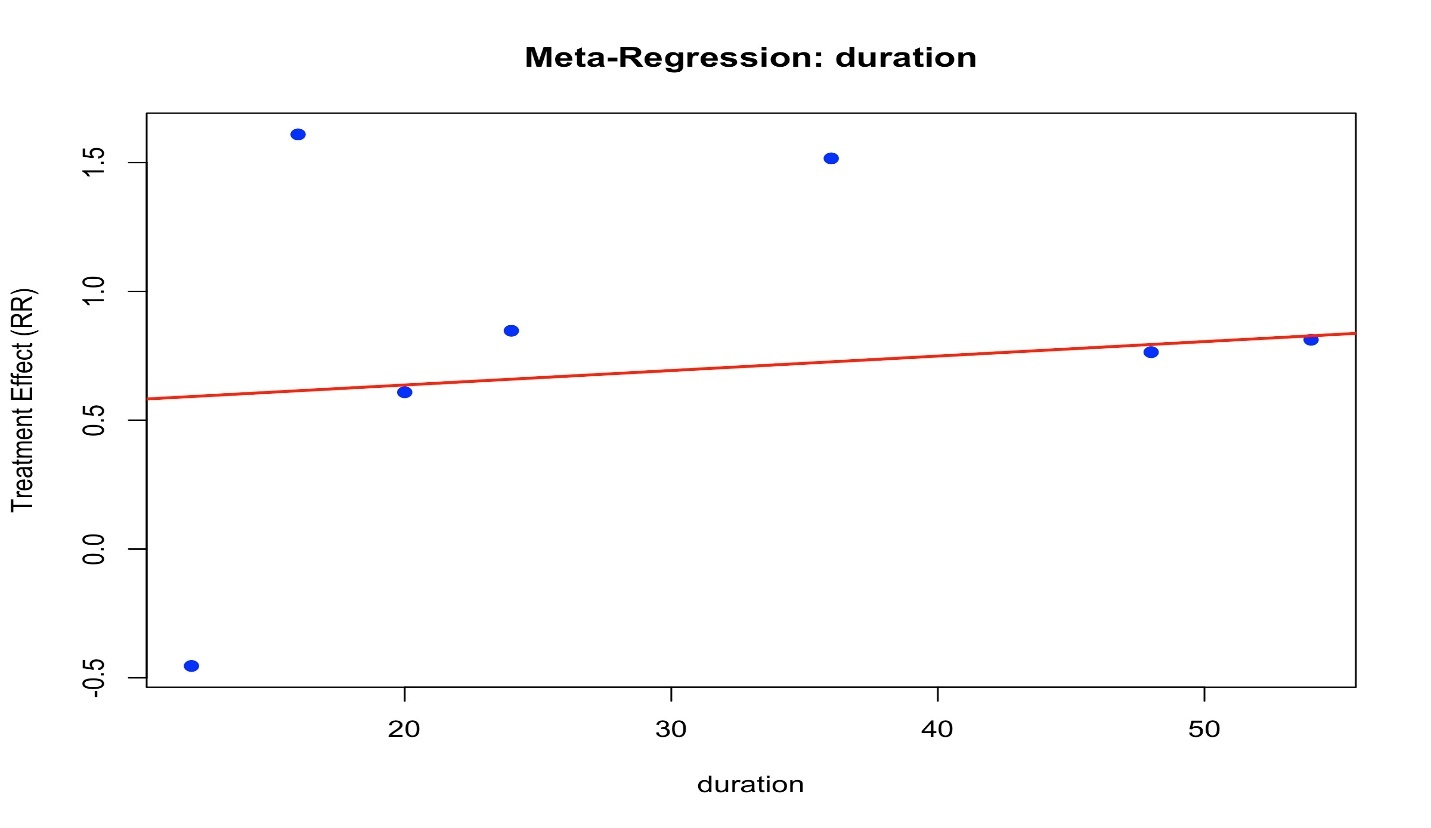


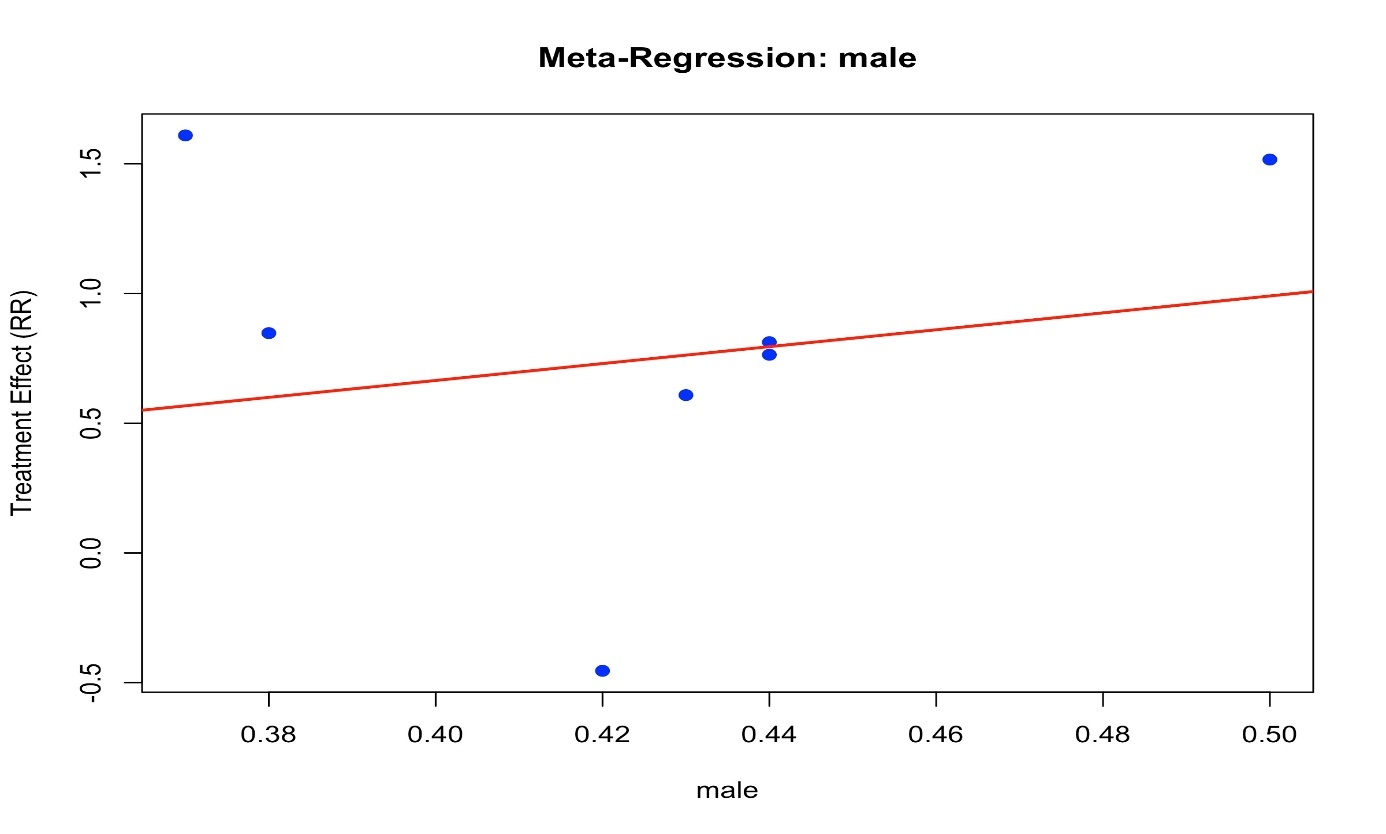


## Figure S10.12 Abdominal pain


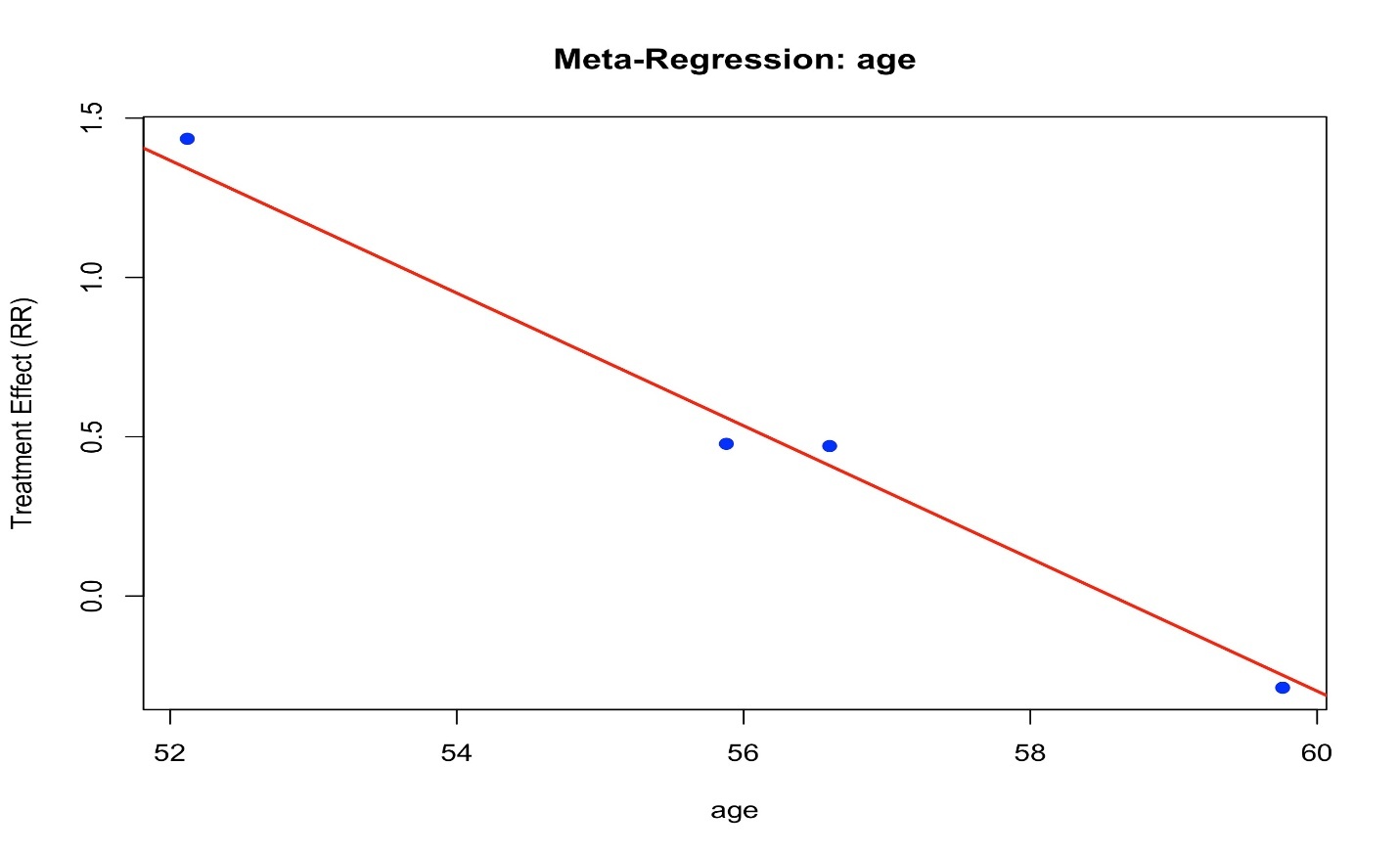


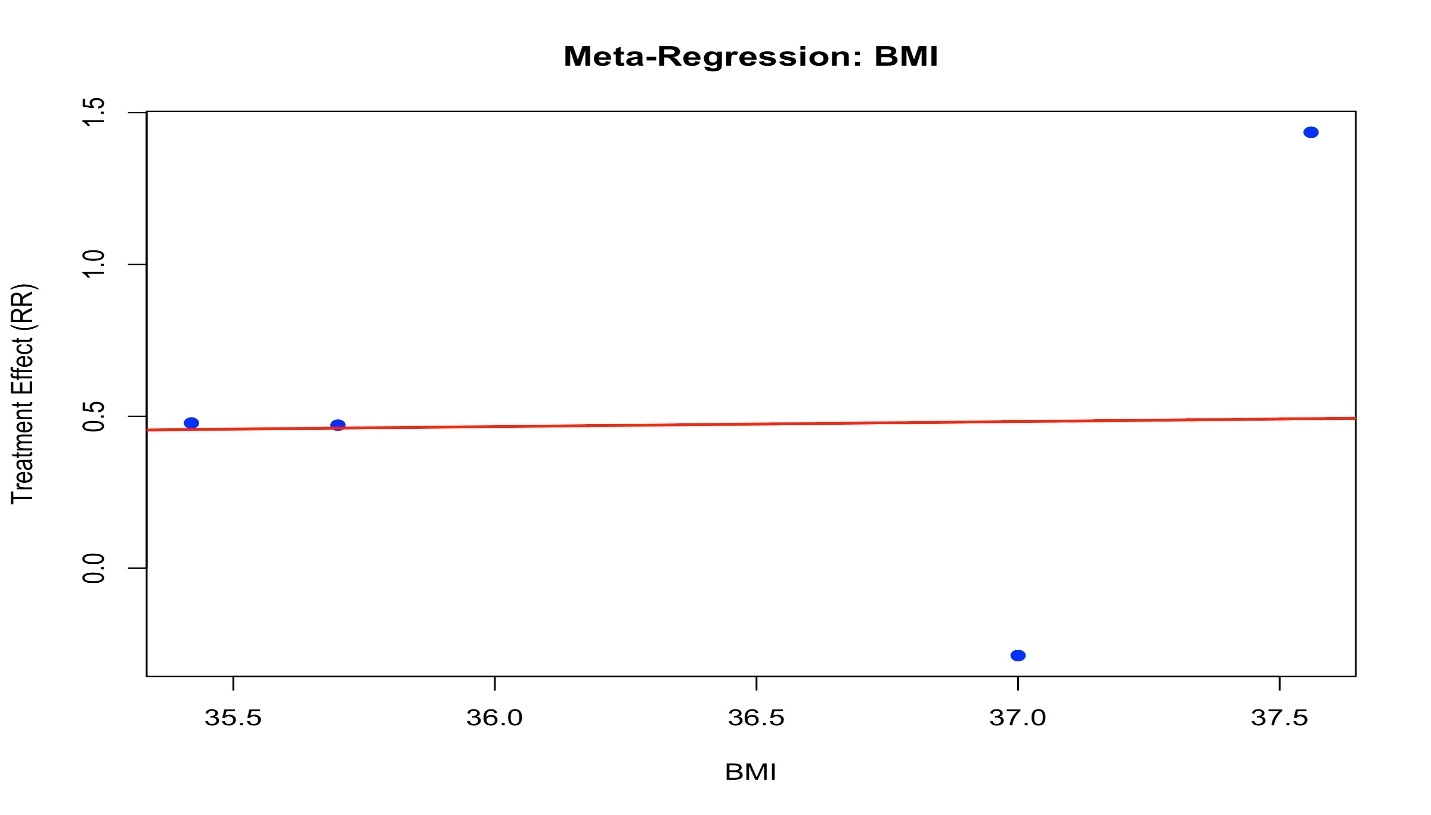


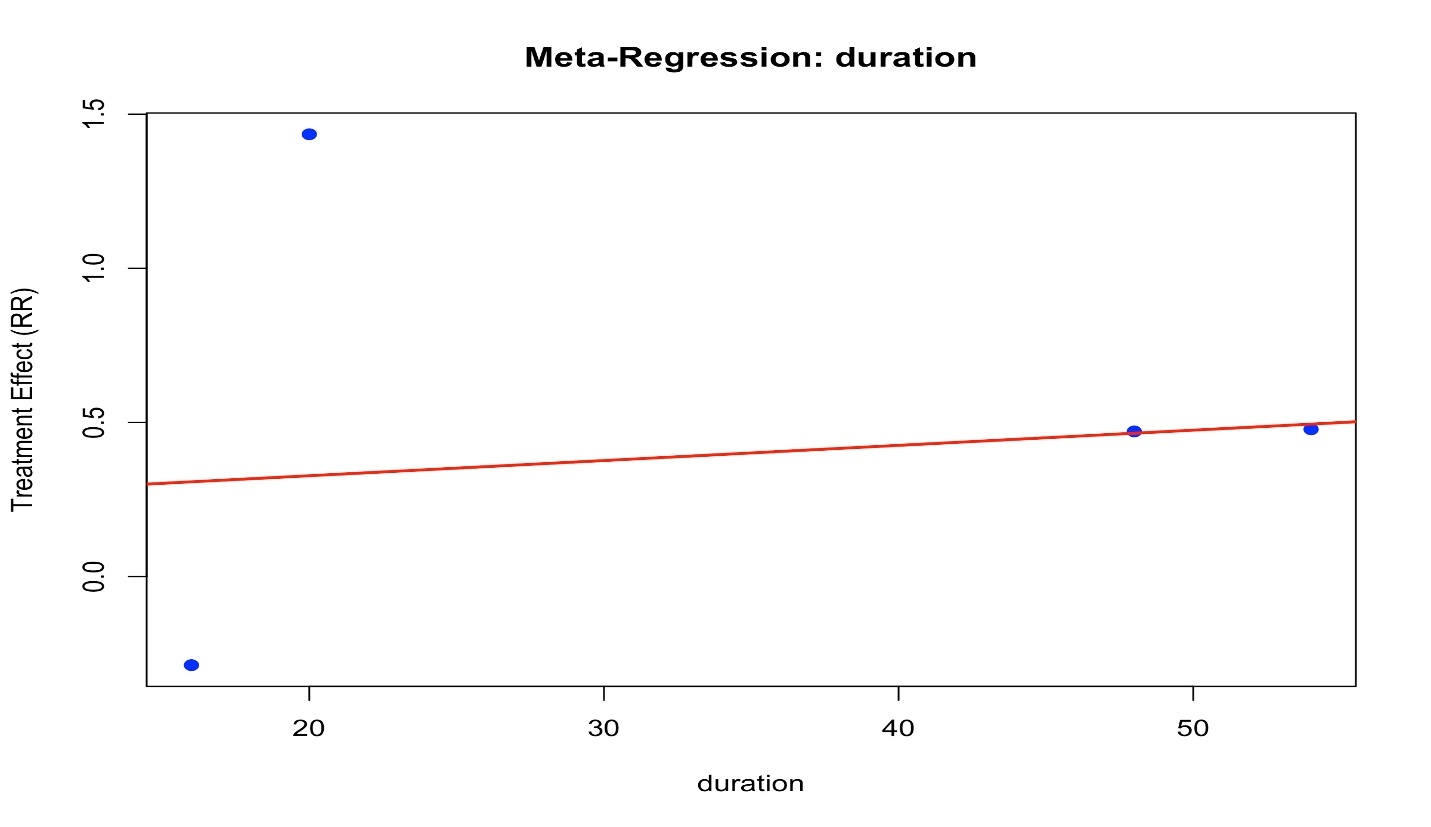


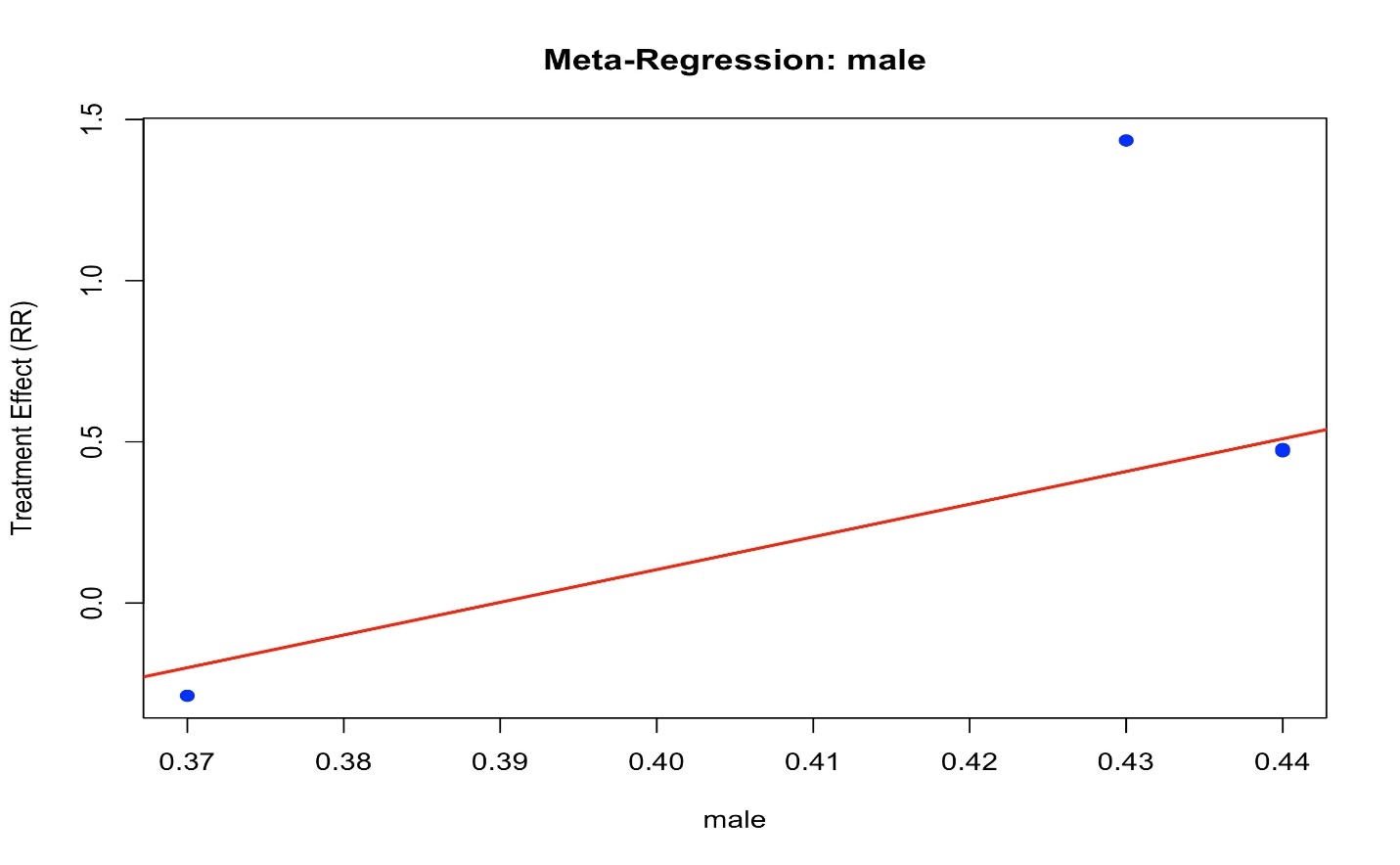


# Supplement S11: PRISMA Checklist

| **Section and Topic** | **Item #** | **Checklist item** | **Location where item is reported** |
| --- | --- | --- | --- |
| **TITLE** | | |  |
| Title | 1 | Identify the report as a systematic review. | 1 |
| **ABSTRACT** | | |  |
| Abstract | 2 | See the PRISMA 2020 for Abstracts checklist. | 2 |
| **INTRODUCTION** | | |  |
| Rationale | 3 | Describe the rationale for the review in the context of existing knowledge. | 3 |
| Objectives | 4 | Provide an explicit statement of the objective(s) or question(s) the review addresses. | 3,4 |
| **METHODS** | | |  |
| Eligibility criteria | 5 | Specify the inclusion and exclusion criteria for the review and how studies were grouped for the syntheses. | 6,7 |
| Information sources | 6 | Specify all databases, registers, websites, organisations, reference lists and other sources searched or consulted to identify studies. Specify the date when each source was last searched or consulted. | 5,6 |
| Search strategy | 7 | Present the full search strategies for all databases, registers and websites, including any filters and limits used. | 6, S1 |
| Selection process | 8 | Specify the methods used to decide whether a study met the inclusion criteria of the review, including how many reviewers screened each record and each report retrieved, whether they worked independently, and if applicable, details of automation tools used in the process. | 6,7 |
| Data collection process | 9 | Specify the methods used to collect data from reports, including how many reviewers collected data from each report, whether they worked independently, any processes for obtaining or confirming data from study investigators, and if applicable, details of automation tools used in the process. | 6,7 |
| Data items | 10a | List and define all outcomes for which data were sought. Specify whether all results that were compatible with each outcome domain in each study were sought (e.g. for all measures, time points, analyses), and if not, the methods used to decide which results to collect. | 6,7,8 |
|  | 10b | List and define all other variables for which data were sought (e.g. participant and intervention characteristics, funding sources). Describe any assumptions made about any missing or unclear information. | 6,7 |
| Study risk of bias assessment | 11 | Specify the methods used to assess risk of bias in the included studies, including details of the tool(s) used, how many reviewers assessed each study and whether they worked independently, and if applicable, details of automation tools used in the process. | 8,9 |
| Effect measures | 12 | Specify for each outcome the effect measure(s) (e.g. risk ratio, mean difference) used in the synthesis or presentation of results. | 7,8 |
| Synthesis methods | 13a | Describe the processes used to decide which studies were eligible for each synthesis (e.g. tabulating the study intervention characteristics and comparing against the planned groups for each synthesis (item #5)). | 8 |
|  | 13b | Describe any methods required to prepare the data for presentation or synthesis, such as handling of missing summary statistics, or data conversions. | 8 |
|  | 13c | Describe any methods used to tabulate or visually display results of individual studies and syntheses. | 8 |
|  | 13d | Describe any methods used to synthesize results and provide a rationale for the choice(s). If meta-analysis was performed, describe the model(s), method(s) to identify the presence and extent of statistical heterogeneity, and software package(s) used. | 8 |
|  | 13e | Describe any methods used to explore possible causes of heterogeneity among study results (e.g. subgroup analysis, meta-regression). | 8 |
|  | 13f | Describe any sensitivity analyses conducted to assess robustness of the synthesized results. | 8 |
| Reporting bias assessment | 14 | Describe any methods used to assess risk of bias due to missing results in a synthesis (arising from reporting biases). | 8,9 |
| Certainty assessment | 15 | Describe any methods used to assess certainty (or confidence) in the body of evidence for an outcome. | 8 |
| **RESULTS** | | |  |
| Study selection | 16a | Describe the results of the search and selection process, from the number of records identified in the search to the number of studies included in the review, ideally using a flow diagram. | 9 |
|  | 16b | Cite studies that might appear to meet the inclusion criteria, but which were excluded, and explain why they were excluded. | 9 |
| Study characteristics | 17 | Cite each included study and present its characteristics. | 9, s2 |
| Risk of bias in studies | 18 | Present assessments of risk of bias for each included study. | 10-15, s12 |
| Results of individual studies | 19 | For all outcomes, present, for each study: (a) summary statistics for each group (where appropriate) and (b) an effect estimate and its precision (e.g. confidence/credible interval), ideally using structured tables or plots. | 10-15 |
| Results of syntheses | 20a | For each synthesis, briefly summarise the characteristics and risk of bias among contributing studies. | 10-15, S4 |
|  | 20b | Present results of all statistical syntheses conducted. If meta-analysis was done, present for each the summary estimate and its precision (e.g. confidence/credible interval) and measures of statistical heterogeneity. If comparing groups, describe the direction of the effect. | 10-15, S6,7 |
|  | 20c | Present results of all investigations of possible causes of heterogeneity among study results. | 10-15 |
|  | 20d | Present results of all sensitivity analyses conducted to assess the robustness of the synthesized results. | 10-15 |
| Reporting biases | 21 | Present assessments of risk of bias due to missing results (arising from reporting biases) for each synthesis assessed. | 10-15, S5 |
| Certainty of evidence | 22 | Present assessments of certainty (or confidence) in the body of evidence for each outcome assessed. | 10-15, S4 |
| **DISCUSSION** | | |  |
| Discussion | 23a | Provide a general interpretation of the results in the context of other evidence. | 18,20 |
|  | 23b | Discuss any limitations of the evidence included in the review. | 20,21 |
|  | 23c | Discuss any limitations of the review processes used. | 20,21 |
|  | 23d | Discuss implications of the results for practice, policy, and future research. | 20,21 |
| **OTHER INFORMATION** | | |  |
| Registration and protocol | 24a | Provide registration information for the review, including register name and registration number, or state that the review was not registered. | 5 |
|  | 24b | Indicate where the review protocol can be accessed, or state that a protocol was not prepared. | 5 |
|  | 24c | Describe and explain any amendments to information provided at registration or in the protocol. | 5,6 |
| Support | 25 | Describe sources of financial or non-financial support for the review, and the role of the funders or sponsors in the review. | 23 |
| Competing interests | 26 | Declare any competing interests of review authors. | 23 |
| Availability of data, code and other materials | 27 | Report which of the following are publicly available and where they can be found: template data collection forms; data extracted from included studies; data used for all analyses; analytic code; any other materials used in the review. | 23 |
